# Molecular and functional analysis of anchorage independent, treatment-evasive neuroblastoma tumorspheres with enhanced malignant properties: A possible explanation for radio-therapy resistance

**DOI:** 10.1371/journal.pone.0189711

**Published:** 2018-01-03

**Authors:** Tamara J. Abou-Antoun, Javad Nazarian, Anthony Ghanem, Stanislav Vukmanovic, Anthony D. Sandler

**Affiliations:** 1 Department of Pharmaceutical Sciences, the School of Pharmacy, Lebanese American University, Byblos, Lebanon; 2 The Sheikh Zayed Institute for Pediatric Surgical Innovation, Children's National Health System, Washington, D.C., United States of America; 3 Department of Integrative Systems Biology, George Washington University School of Medicine and Health Sciences, Washington, D.C., United States of America; 4 Center for Genetic Medicine Research, Children’s National Medical Center, Washington, D.C., United States of America; 5 The School of Medicine, Lebanese American University, Byblos, Lebanon; 6 The Joseph E. Robert Center for Surgical Care, Children's National Health System, Washington, D.C., United States of America; Northwestren University, UNITED STATES

## Abstract

Despite significant advances in cancer treatment and management, more than 60% of patients with neuroblastoma present with very poor prognosis in the form of metastatic and aggressive disease. Solid tumors including neuroblastoma are thought to be heterogeneous with a sub-population of stem-like cells that are treatment-evasive with highly malignant characteristics. We previously identified a phenomenon of reversible adaptive plasticity (RAP) between anchorage dependent (AD) cells and anchorage independent (AI) tumorspheres in neuroblastoma cell cultures. To expand our molecular characterization of the AI tumorspheres, we sought to define the comprehensive proteomic profile of murine AD and AI neuroblastoma cells. The proteomic profiles of the two phenotypic cell populations were compared to each other to determine the differential protein expression and molecular pathways of interest. We report exclusive or significant up-regulation of tumorigenic pathways expressed by the AI tumorspheres compared to the AD cancer cells. These pathways govern metastatic potential, enhanced malignancy and epithelial to mesenchymal transition. Furthermore, radio-therapy induced significant up-regulation of specific tumorigenic and proliferative proteins, namely survivin, CDC2 and the enzyme Poly [ADP-ribose] polymerase 1. Bio-functional characteristics of the AI tumorspheres were resistant to sutent inhibition of receptor tyrosine kinases (RTKs) as well as to 2.5 Gy radio-therapy as assessed by cell survival, proliferation, apoptosis and migration. Interestingly, PDGF-BB stimulation of the PDGFRβ led to transactivation of EGFR and VEGFR in AI tumorspheres more potently than in AD cells. Sutent inhibition of PDGFRβ abrogated this transactivation in both cell types. In addition, 48 h sutent treatment significantly down-regulated the protein expression of PDGFRβ, MYCN, SOX2 and Survivin in the AI tumorspheres and inhibited tumorsphere self-renewal. Radio-sensitivity in AI tumorspheres was enhanced when sutent treatment was combined with survivin knock-down. We conclude that AI tumorspheres have a differential protein expression compared to AD cancer cells that contribute to their malignant phenotype and radio-resistance. Specific targeting of both cellular phenotypes is needed to improve outcomes in neuroblastoma patients.

## Introduction

Despite continuous advancement in cancer therapy approaches, neuroblastoma (NB) recurrence with metastatic disease remains a major concern with poor prognostic outcomes [1]. NB is the most common extra-cranial solid tumor in children. NBs occurring in early childhood have a more favorable outcome compared to the late-onset disease that carries the poorest prognosis [1]. The clinical course of NB is often variable, ranging from spontaneous regression to inevitable progression and mortality [2]. Most NB patients respond to treatment protocols and undergo regression and a state of minimal residual disease. High-risk cases present with a very aggressive form of the disease with treatment-evasive properties and malignant recurrence that is usually fatal. Extensive research has focused on the cancer stem-cell theory in an attempt to elucidate the mechanisms utilized by aggressive cancers to evade therapy and lead to lethal recurrence. It is considered that a sub-population of malignant, treatment-resistant cells reside within the bulk of many solid and hematologic tumors [3]. These cells do not undergo apoptosis with therapeutic intervention, but rather remain dormant for a period of time, ranging from months to years, after which they repopulate themselves giving rise to the original tumor with a highly aggressive phenotype and malignant properties including treatment-resistance [4].

The aggressive nature of this NB sub-population led us to investigate the presence of cancer stem-like cells within the bulk of these cancers. We recently showed that neuroblastoma is a stem like disease [2] in the sense that cancer cells exhibit plasticity in their ability to adapt to certain physiological stressors by undergoing phenotypic transformations that allow them to withstand such stressors [2]. These cells underwent AI tumorsphere transformation when grown under serum free, stressed conditions, and reverted back to AD adhered cells after returning to their serum rich, physiologically-friendly environment. We also observed variations in gene expression between the stressed versus non-stressed cells [2]. Moreover, Chakrabarti et al further reported a 20 fold overexpression of the inhibitor of differentiation 2 (Id2) in the AD compared to the AI and demonstrated crosstalk between Id2-TGFβ pathways in AD phenotypes [5]. In order to validate the transcriptome finding, we aimed at determining the proteomic profile of the two distinct (AD and AI) phenotypes of murine NB cells. Moreover, we tested and compared the effects of radiation and receptor tyrosine kinase (RTK) inhibition using Sunitinib malate (Sutent^®^) on AI and AD cells. Finally, we examined the protein expression of tumorigenic markers at baseline and after radio-therapy of the phenotypically-distinct cells. We found AI tumorspheres to express up-regulation of highly tumorigenic pathways compared to the AD counterparts. These pathways are reported to govern metastatic potential and increased malignancy, epithelial to mesenchymal transition and to be associated with poor prognosis (PDGFRβ, EGFR, HDGFR, MIA3, PARP1, SOX2, VEGFR2 and RhoA). Of particular interest was the up-regulation of tumorigenic proteins survivin, CDC2 as well as the DNA repair enzyme PARP after radio-therapy. AI tumorspheres were resistant to 2.5 Gy radio-therapy and 0.2 μM sutent treatment, as determined by assessing the rate of cell proliferation, cytotoxicity, apoptosis, and migration after treatment. The PDGFRβ was phosphorylated in the AI tumorspheres by exogenous PDGF-BB stimulation that was not observed in the AD cells, which was abrogated with sutent treatment. Interestingly, both AD cells and more potently AI tumorspheres exhibited a PDGF-BB induced trans-activation of EGFR and VEGFR, which was abrogated with sutent treatment. Furthermore, the long-term effect (48 h) of sutent treatment in AI tumorsphere revealed significant down-regulation of the tumorigenic proteins PDGFRβ and survivin as well as the stemness drivers MYCN and SOX2 and inhibited tumorsphere self-renewal. Combining survivin knock-down with sutent treatment enhanced radio-sensitivity in neuro2a AI tumorspheres. We speculate that the over-expressed tumorigenic proteins identified in the AI tumorspheres may play a critical role in the treatment-evasive and malignant nature of these phenotypically and molecularly distinct cells.

## Materials and methods

### Murine cell lines

Neuro2a is the murine neuroblastoma cell line which is derived from AJ mice, purchased from the American Type Culture Collection (Manassas, NJ) where cells were characterized and authenticated using standardized methods at ATCC. Cells were cultured in DMEM containing 10% fetal calf serum, 0.5% penicillin/streptomycin, 10% L-glutamine, 10% nonessential amino acid and 10% pyruvate. Cells were sub-cultured for less than 6 months after acquiring them from ATCC, the time during which experiments were conducted.

### Derivation of stem-like cells and culture media

Neuro2a cells were cultured in NeuroCult complete media supplemented with factors that enrich for the neural stem-like cells within the bulk population of neuroblastoma cells. NeuroCult complete consists of NeuroCult Neural Stem Cell (NSC) Basal medium (Mouse) (Stem Cell technologies, cat # 05700), 1/10 NeuroCult NSC Proliferation supplements (Mouse) (Stem Cell Technologies, Cat # 05701), 20 ng/ml rh EGF, 10 ng/ml rh FGF-b and 2 μg/ml Heparin.

### Tumorsphere self-renewal assay

A single cell suspension was derived from dissociated AI tumorspheres and seeded into a 96-well plate in a limited-dilution assay. Tumorsphere formation was assessed by microscopically visualizing the wells of the 96-well plate over the course of 15 days after seeding and scored as (+) or (–) for presence of a tumorsphere or not, respectively. A well is considered to contain a tumorsphere if a single cell yielded a tumorsphere of approximately ≥ 5 cells after 15 days of seeding.

### siRNA transfection

Four survivin (BIRC) siRNA oligonucleotides were purchased from Qiagen and used as a cocktail to knock-down survivin protein expression in the AD cells and AI tumorspheres using OptiMem (Lonza, Bassel Switzerland) and HiperFect (Qiagen, Germany) transfection reagent. Cells were grown in a 6-well plate until they reached 70–80% confluency after which they were transfected with either survivin siRNA at a concentration of 2 μM for 6 h or with mock-transfection reagent. The transfection media was then removed and cells were cultured in their respective media for the length of the experiment.

### Cytotoxicity assay

Control and sutent- or radiation-treated cells were seeded in a 96-well plate at a density of 5 x 10^3^. The cells were seeded in six replicates per group and analyzed for cytotoxicity at 24, 48 and 72 h after radiation or sutent treatment. At each time point, an XTT assay was used to determine the percent of cell death in the cells. One hundred microliters of electron coupling reagent was mixed with 5 ml of XTT labeling reagent and 50 μl of the mixture added to the wells of the 96-well plate and left at 37°C for 3 h until a colorimetric change was observed in the wells. The absorbance of the colorimetric change in the treated versus the non-treated cells was measured on a plate reader at 450 nm. The percentage of dead cells was calculated using the following formula: 1- (absorbance of treated cells / absorbance of control cells) X 100. Percentages were plotted on a graph using Microsoft Excel. Experiments were conducted in six replicates per group and the results represent the mean ± the standard error of the mean of multiple experiments.

### Cell proliferation assay

Proliferation rate was determined by the colorimetric absorbance of the WST-1 assay at 24, 48, 72 and 96 h post treatment that measures the cleavage of tetrazolium salt in metabolically-active cells. The average absorbance at 450nm of each group, run in six replicates, was plotted on a line graph using Microsoft Excel. The mean ± the standard error of the mean of multiple experiments was graphed and a Student’s t test used to determine statistical significance of the means between groups. When comparing the AD and AI cells in the same experiments, due to the different culture condition, we derived the average ratio of treatment/control of each cell phenotype (AD or AI) of 4 independent experiments ± SEM and derived the statistical significance using a Students T-test.

### Ki-67 staining

Tumorspheres and adhered cells were collected and pelleted by centrifuging at 400 rpm for 5 mins at 4°C. Tumorspheres were dissociated into a single-cell suspension using the NeuroCult Chemical Dissociation Kit (Stem Cell Technologies, Canada) following the manufacturer’s protocol. The dissociated tumorspheres were filtered through a 40μm cell strainer and cell viability determined on a hemocytometer using trypan blue. While vortexing, 5 ml cold 70%–80% ethanol was added drop-wise into the cell pellet (1–5 x 10^7^ cells) and incubated at -20°C for at least 2 h. Fixed cells were washed twice with 30–40 ml staining buffer (PBS with 1% FBS, 0.09% NaN_3_), centrifuged for 10 minutes at 200 x g and resuspended in PBS to a concentration of 1 x 10^7^/ml. Around 1 x 10^6^ cells (100 μl) were transferred into each sample tube and 20 μl of properly diluted anti-Ki-67 antibody (clone B56) according to the manufacturer’s protocol was added into the tubes above. Tubes were mixed gently and incubated at room temperature for 20–30 minutes in the dark, then washed with 2 ml of staining buffer at 200 x g for 5 minutes and Ki-67 staining determined using FACS Calibur analysis. These experiments were conducted 4 independent times and the average ratio of the percent ki-67 positive staining before and after radiotherapy for the AD and AI groups was calculated and a Student’s T-test used to determine the statistical significance between the average ratios of AD and AI cells.

### Cell viability assay

Treated and un-treated cells were manually counted on a hemocytometer to determine the percent cell viability at 24, 48, 72 and 96 h post treatment after staining with trypan blue. This method was used in support of the 7AAD/Annexin V and XTT assays.

### Apoptosis (7AAD/Annexin-V) assay

Irradiated and non-irradiated AI tumorspheres or AD adhered cells were reseeded in triplicate T 25 cm^2^ flasks and incubated overnight at 37°C. The next day, fresh media was added to the cells and they were incubated for an additional 24 h at 37°C. After the incubation, cells were harvested, tumorspheres dissociated, and all cells counted on a hemocytometer using trypan blue. Approximately 1 x 10^6^ cells were washed 2x with 1X Annexin binding buffer and then stained with 7-AAD and Annexin V at a 1:40 dilution in Annexin binding buffer for ~ 20 min. Cells were analyzed for Annexin V/7-AAD expression within the hour using FACS Calibur.

### Cell migration and invasion assays

Serum-starved (24 h) AI tumorspheres, that required dissociation into a single-cell suspension, and AD cells, were counted on a hemocytometer and a suspension of 1 x 10^6^ cells per ml was made in serum-free media. The QCM-Cell migration or invasion assay (Millipore) was used to determine the rate of migration or invasion between AI tumorspheres and AD cells after drug-treatment (0.2 μM sutent) or radio-therapy (2 Gy). Collagen-coated, 8μm trans-well migration chambers were placed in each well of a 24-well plate. Three hundred micro-litters of the prepared cell suspension were added to the upper side of the migration chamber. Five hundred micro-litters of serum-free media, with or without chemo-attractant (10 ng/ml PDGF-BB), was added to the lower chamber. The plates were covered and stored at 37°C for 16 h. Non-migrating cells were removed with a cotton swab and the migrated cells stuck to the lower side of the membrane were stained using a cell stain solution (Chemicon, part no. 20294) for 10mins. The stain was extracted using stain extraction buffer (Chemicon, part no. 20295) and 100μL of the dye was loaded into wells of a micro-plate and quantitated using a colorimetric plate reader at an absorbance of 570OD. These experiments were conducted in triplicates and repeated 4 independent times. The average absorbance measured represents the mean ± SEM of the 4 independent experiments. Results were graphed as a ‘Fold-Change’, where the control groups (AD or AI) were normalized to 1 and the treatment groups presented as ‘Fold-Change’ compared to the control group of each phenotypic cell type. An ANOVA test was used to determine statistical significance between the three conditions (control, control + PDGF-BB and sutent pre-treatment + PDGF-BB). Statistical significance was set at 0.05.

### PDGFR stimulation

Cells were serum starved (1x10^6^) for 24 h in 6 ml serum-free growth media in T-75cm^2^ flasks. The next day cells were washed (3x) with serum-free media to remove endogenous growth factor contamination, replenished with 6 ml serum-free media and then treated with PDGF-BB (10 ng/ml) or PBS for 5 mins at 37°C prior to cell harvesting for immediate cellular protein expression using Western blot analysis as detailed below.

### Immunoblot analysis

Western blot analysis was used to determine the level of PDGFRβ, EGFR, VEGFR, AKT, ERK, survivin, SOX2, PARP1, HDGFR, and CDC2 protein expression between the two types of NB cells. Whole cell lysates were prepared from NB AI tumorspheres and AD adhered cells in 1X RIPA Cell Lysis Buffer (Cell Signaling Technology). The protein concentration was determined using the BCA assay (Thermo Fisher). An aliquot of the lysate was mixed with an equal volume of 2x Laemli Sample buffer and heated at 97°C for 10 mins. Between 20 and 40 μg of total protein concentration was electrophoresed on a 4–12% SDS-PAGE and transferred onto a PVDF membrane (Perkin Elmer, Waltham, MA). Target proteins were detected using primary antibodies for total or phosphorylated forms of the PDGFRβ (rabbit pAb; Abcam 1:500; Cat# ab32570; rabbit pAb; Abcam 1:250; Cat# ab16868), Akt (rabbit pAb; Abcam; Cat#ab38449; Cat#ab8805, respectively) (1:500; 1:250) and Erk1/2 (rabbit pAb;1:500 Cell Signaling Technologies; Cat# 9102; rabbit pAb; 1:250 Santa Cruz Technologies; Cat# sc-101761) (for S2 Fig we used the sampler kit rabbit mAb; Cell Signaling Technologies Cat# 12651), and to total and phosphorylated expression of EGFR (Abcam rabbit mAb; 1:500 Cat#ab52894; 1:250 Cat#ab40815), MIA3 (Abcam rabbit pAb; 1:250 Cat#ab97881), RhoA (Abcam; rabbit pAb 1:500 Cat#ab86297; HeLa cell lysates used as positive control), cleaved PARP1 (1:500; Cell Signaling Technologies Cat# 5625), HDGFR (Abcam; rabbit pAb 1:1000 Cat#210683; HeLa cell lysates used as positive control), VEGFR2 (rabbit pAb; Abcam Cat# ab39256) survivin (rabbit mAb; 1:500, Cell Signaling Technologies Cat#2808), Sox2 (Abcam; rabbit pAb; 1:500; Cat#137385) and CDC2 (Cell Signaling Technology; rabbit pAb; 1:1000 Cat# 77055) using the above dilutions. The blots were incubated with the primary antibody overnight at 4°C, washed (3x) 5 mins each and incubated with secondary antibody 1 h at room temperature using the anti-rabbit IgG HRP-conjugated (Cell Signaling Technology) antibody at a dilution of 1:2000. The blots were then washed (4x) 15 mins each with 1 x TBS-Tween (0.1%) and incubated in SuperSignal Dura chemiluminescent substrate (Pierce, Rockford, IL) ~2 mins and then exposed. To control for protein loading variations, a primary antibody for GAPDH (Cell Signaling Technology) was used, and the total protein for PDGFRB, Akt and Erk1/2 was used to measure the ratio of total:phosphorylated protein, and quantitated by densitometric analysis using ImageLab 5.2 software Bio-Rad.

### Proteomic data and Statistical analysis

Total protein was extracted from neuroblastoma AI tumorspheres and AD adhered cells, using RIPPA buffer (Sigma-Aldrich, St. Louis, MO). Whole cell lysates (100ug) were reduced with 5mM DTT in Laemmli buffer (Bio-Rad, Hercules, CA) and resolved on a 4–12% SDS polyacrylamide gel. The gel was fixed in acetic acid:methanoal (1:1 v/v) for 30 minutes and stained with Commassie blue (1 h) and destained in water over night. Each protein lane was then cut into 36 bands and each band was subjected to in-gel digestion as described before [6]. Briefly, proteins from each band were digested with trypsin and sequenced by Liquid Chromatography Mass Spectrometry/ Mass Spectrometry (LC-MS/MS) and identified by searching against murine databases. We used ProteoIQ software (Bioinquire, Atlanta GA), for our analysis as previously described [7, 8]. The advantage of ProteoIQ is its ability to extract the spectral counts (SCs) for each peptide and compare it across several samples. Our initial analysis showed a linear expression of the majority of proteins. A total of 1,069 proteins were identified using the following stringent criteria: Protein quantification was performed using ProteoIQ software (Bioinquire) with spectral count data. The following stringent filtration criteria were used: 2 or more peptides/protein, 10 or more spectra/protein, minimum peptide probability-based score with *P* ≤ .05, and a variable threshold of *Xcorr* versus charge state (*Xcorr* = 1.9 for *z* = 1, *Xcorr* = 2.5 for *z* = 2, and *Xcorr* = 3.5 for *z* = 3). Protein expression analysis was performed using Partek Genomics Suite (Partek). The raw data has been deposited in the Open Science Framework repository and can be accessed at: osf.io/8y2wv.

### Pathway analysis and data interpretation

Pathway analysis was performed using Ingenuity Pathway Analysis software, version 1.0 (Ingenuity Systems). Accession numbers of the 1069 identified neuroblastoma AI tumorpshere and AD cell proteins were uploaded, and protein-protein interaction networks were generated using fold-change protein expression data. Comparison analysis of fold-change expression data was performed to reveal top molecules and pathways of interaction in the two cell types. Pathways of protein expression and interaction were explored using tumor specific protein sets and known pathways with biological relevance to cancer, cell-to-cell signaling, small molecule biochemistry, RNA post translational modification and energy production and metabolism.

String-functional protein association networks was used to illustrate protein-protein interactions between our identified proteins and other proteins with relevance to cancer, mitogenic and DNA-binding activity, histone methylation, cell proliferation, differentiation, adhesion, migration and invasion.

### Validation of proteomic analysis

Tumorigenic proteins of interest specific to the cell types that were identified by proteomic analysis were selected and their expression was confirmed by immunoblot (Western Blot) to validate specificity as described above.

### Statistical analysis

A two-tailed Student’s t test was used to determine statistical significance between groups. Results of proteomics profile between AI tumorspheres and AD cells were filtered on the basis of a variable threshold of *Xcorr* versus charge state and peptide probability with *P* < .05. An ANOVA test was used to compare multiple groups in single experiments. Statistical significance was set at a P value ≤ 0.05.

## Results

### Differential proteomic profile expression between neuroblastoma AI tumorspheres and AD cells

**Fig 1:**
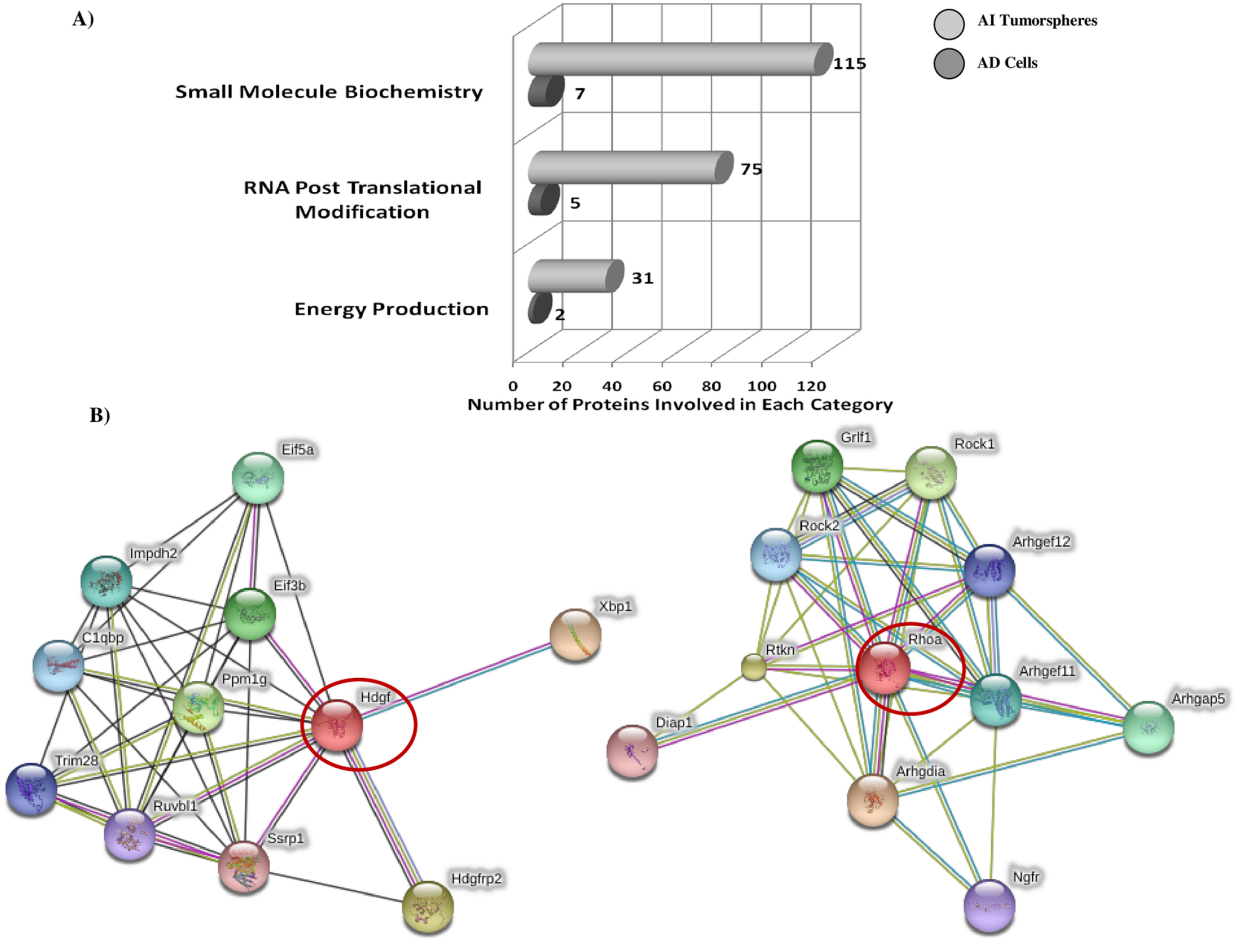
Proteomic profile analysis reveals functional categories of proteins differentially expressed between AI tumorspheres and AD neuro2a cells. (A) Compared to NB AD adhered cells, AI tumorspheres are comprised of proteins that are highly involved in small molecule biochemistry, RNA post translational modification and energy production and metabolism. (B) String Functional Protein pathway analysis revealed affiliations between the tumorigenic proteins in the AI tumorspheres, specifically highlighted are HDGFR and RhoA pathways and their affiliated interactions with other tumorigenic proteins. The HDGFR affiliated proteins are involved in mitogenic and DNA-binding activity and may play roles in cellular proliferation and differentiation and histone methylation; whereas the RhoA affiliated proteins play key roles in the GDP-GTP exchange and activation of Rho-GTPs involved in cell adhesion, migration and invasion.

In order to define specific characteristics and molecular components of the AD and AI cell phenotypes, we generated the comprehensive protein profile of these cells and compared them to each other. Total protein was extracted from each sample and analyzed using label-free LC-MS/MS as described in the *Method*s section. Stringent proteomic filtration identified a total of 1,069 proteins expressed between AD and AI cells (S1 Table). Of these, a total of 177 proteins were shared between the AD and AI cells (S2 Table). Functional analysis of differentially expressed proteins indicated specific upregulation in proteins involved in small molecule biochemistry, RNA post translational modification, and energy metabolism in AI cells compared to AD cells (Fig 1A).

Among the significantly-higher expressed proteins in the NB AI tumorspheres that we validated using Western blot analysis, was the Poly (ADP-ribose) polymerase 1 (PARP1), melanoma inhibitory activity 3 (MIA3), RhoA and hepatoma derived growth factor-related protein (HDGFR), which are associated with increased metastases, stemness, invasion and malignancy [9, 10, 11, 12]. Furthermore, other proteins involved in tumorigenic pathways expressed either exclusively or significantly higher (Table 1) in the AI tumorspheres compared to the AD cells were also identified by proteomics analysis. Most notably were the proteins involved in epithelial-to-mesenchymal transition (EMT) including: ROCK II, Anxa2, Tpi1, Pgk1, Hspa8, Hnrnpk, Nampt, FASN and Rho A (validated with WB), and stem-ness maintenance (Supt16h or FACT) which may explain the phenotypic transformation induced by the AI tumorspheres upon stressful culture conditions. Although this tumor is not epithelial, the AD to AI transition may represent a phenomenon similar to EMT. In addition, proteins associated with poor prognosis, such as the DNA polymerase delta catalytic subunit 1 (POLD1) and radio-resistance, such as PARP1 (validated with WB) may also explain the aggressive nature and radio-resistance of AI tumorspheres compared to AD cells. Finally, proteins involved in tumor migration, invasion, malignancy and cell adhesion (Ctsd or Cathepsin D [13], MTCO2, Tpd52l2 [14], Rab5c [15], Smarcc1 [16]) were also found to be exclusively expressed or significantly up-regulated by the AI tumorspheres compared to the AD cells (Table 1). Specific molecules were detected that are indicative of high proliferative rate, including cell division control protein 2 (CDC2) (validated with WB), a cell cycle progression protein, that was up-regulated in AD cells.

**Table 1:** Highlighting proteins exclusively or significantly over-expressed in the AI tumorspheres compared to the AD cells and their tumorigenic role.

Protein Expression	Proteins	Tumorigenic Role	Validated with WB
**Exclusive to AI**	ROCK II Anxa2, Annexin A2, Sdpr, PACSIN2	EMT marker associated with TGF-β	✓
	MIA3	Malignancy, Validated by WB	✓
	MTCO2, cytochrome C Oxidase subunit 2; Ctsd (Cathepsin D)	Invasion and malignancy	
	Camk2d	Anti-apoptotic	
	HDGFR	Malignancy	✓
**> 2-fold in the AI**	PARP1	DNA repair, Radio-resistance and Poor prognosis	✓
	Rho A Rab5c, Ras-related protein Rab-5C Tpi1, Triosephosphate isomerase Pgk1, Phosphoglycerate kinase 1 Hspa8, Heat shock cognate 71 kDa protein Hnrnpk, Heterogeneous nuclear ribonucleoprotein K Nampt, Nicotinamide phosphoribosyltransferase	EMT marker, invasion and malignancy	✓
	Hsp70 ERBB Smarcc1, SWI/SNF complex subunit SMARCC1	Malignancy	✓
	Smc4 and Supt16h	Stem-ness maintenance	

Murine AI tumorspheres, and AD cell lysates were processed for protein profiling using mass spectrometry and resulting data underwent stringent proteomic filtration following comparative analysis. Some of the exclusively, or significantly (≥ 2-fold) over-expressed proteins in the AI tumorspheres compared to the AD cells are listed with the affiliated tumorigenic roles indicated and whether Western blot analysis (✓) validated the proteomic findings.

Pathway analysis showed that the majority of up-regulated proteins in AI tumorspheres constituted four general pathways: i) cellular function and maintenance and carbohydrate metabolism, ii) lipid metabolism, and energy metabolism, iii) tissue morphology, and iv) cellular assembly and organization (Table 2). More importantly, compared to AD cells, AI cells showed up-regulation of proteins involved in small molecule biochemistry (indicating high metabolic activity), RNA post translational modification, and energy production. String protein pathway analysis revealed interactions between HDGFR and RhoA and other tumorigenic proteins such as Ssrp1 and Ruvbl1 in connection with HDGFR as well as ROCKI and ROCK II in association with RhoA that may be fueling the malignant, invasive and metastatic potential of these cells (Fig 1B). Comparative analysis of metabolic markers between AD and AI cells revealed that Glucose-6-P-1 dehydrogenase was down-regulated while glutathione-S-transferase was up-regulated in the AI cells suggesting diminished glucose metabolism. Simultaneously, Acyl-CoA binding protein and Acetyl-CoA acetyl transferase were both up-regulated in AI cells signifying increased Fatty acid metabolism.

**Table 2:** Upregulated proteins involved in four major functions associated with AI tumorspheres. Gene IDs are provided in parenthesis.

Function	Proteins Involved (Gene ID)
**Cellular Function and Maintenance; Carbohydrate Metabolism**	SERPINH1 (12406); AIFM1 (26926); LAMP2 (16784); APOE (11816); CAMK2B (12323); CAT (12359); CLIP2 (269713); CTTN (13043); DOCK1 (330662); GCLC (14629); GLRX (93692); GSN (227753); HIP1 (215114); HIP1R (29816); KTN1 (16709); LRP1 (16971); MYO6 (17920); NCALD (52589); NEDD4 (17999); PEA15 (18611); PRKAR2A (19087); PSAP (19156); TNC (21923)
**Lipid Metabolism; Small Molecule Biochemistry; Energy Production**	LNPEP (240028); ACAA1 (113868); ACAA2 (52538); ACOX1 (11430); ACSBG1 (94180); ANXA3 (11745); ASRGL1 (66514); CA2 (12349); DDAH1 (69219); EPDR1 (105298); GM2A (14667); HEXA (15211); NQO1 (18104); PECI (239860); PPIC (19038)
**Tissue Morphology**	ALDH1L1 (107747); ANK2 (109676); ASPH (65973); CYB5A (109672); EPHX1 (13849); ESD(13885); GLOD4 (67201); GSTO1 (14873); HIBCH (227095); ILF3 (16201); PBXIP1 (229534); PPP1R12A (17931); SLC1A3 (20512)
**Cellular Assembly and Organization**	ACADM (11364); ACADS (11409); COX6C (621837); GLB1 (12091); HRSP12 (15473); NDUFA5 (68202); NDUFA6 (67130); NDUFA12 (66414); NDUFA13 (67184); NDUFB4 (68194); NDUFV2 (72900); NES (18008); RAB31 (106572); STAT3 (20848)

The AI tumorspheres exhibited up-regulated expression of protein involved in four major functions including, i) cellular function and maintenance and carbohydrate metabolism, ii) lipid metabolism, and energy metabolism, iii) tissue morphology, and iv) cellular assembly and organization, compared to AD cells. The p-value calculated by IPA based on the number of proteins present and dysregulated per pathway < 0.05.

Loss of matrix attachment results in metabolic defects with ATP deficiency that is rescued by the activation of oncogene ERBB2 pathway [17]. Although ERBB2 protein was not detected by mass spectrometry, functional pathway analysis showed downstream targets of ERBB2 were indeed up-regulated in AI tumorspheres (S1 Fig) including tumor protein D52-like 2 (TPD52L2), POLD1 catalytic subunit, and pre-mRNA processing factor 40 (PRPF40A). In addition another protein that showed up-regulation (2 fold, *P < 0*.*05*) in AI tumorspheres compared to AD cells is Serum-Deprivation Response Protein (SDRP), a known substrate for protein kinase C phosphorylation which becomes up-regulated in serum-starved cells. Moreover, protein kinase C and casein kinase substrate in neuron (PACSIN2) was also found to be exclusively expressed in our AI tumorspheres compared to the AD cells.

### Validation of specific proteins identified in the proteomic analysis

**Fig 2:**
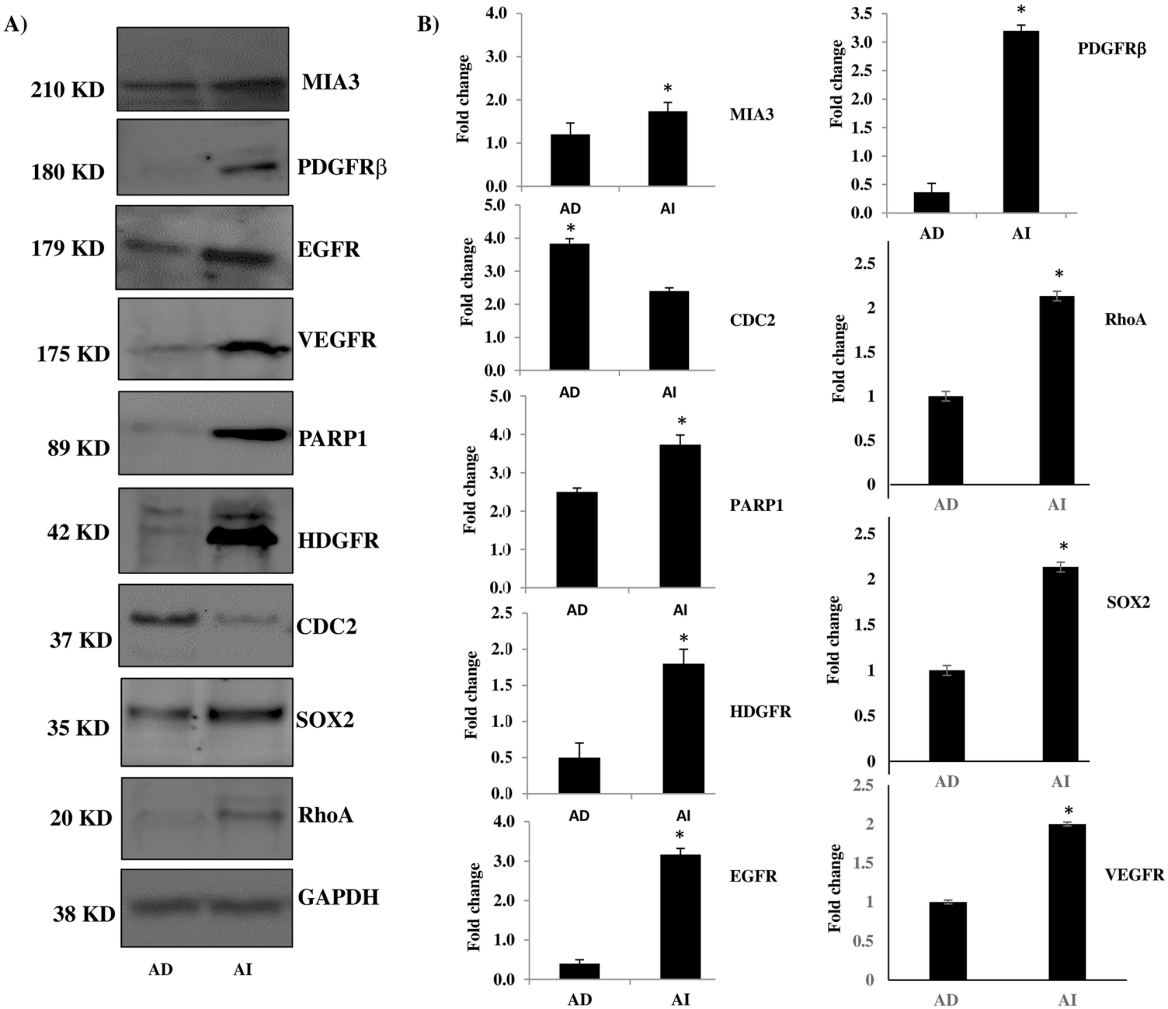
Western blot validation of specific proteins identified in the proteomic analysis. Western blot analysis was used to validate some of the targets identified on proteomic profiling. The protein expression of CDC2 was 2-fold higher in the AD cells compared to the AI tumorspheres, and PARP1, SOX2 and HDGFR were expressed > 1.5-fold higher in the AI tumorspheres compared to the AD cells. In addition, the expression levels of MIA3, RhoA, PDGFRβ, VEGFR and EGFR was also significantly (*P < 0*.*05*) higher in the AI tumorspheres, as determined with Western blot analysis. * indicates a *P value < 0*.*05*. Results represent the mean ± SEM of multiple experiments.

To further validate target molecules that were identified by proteomic analysis, Western blotting assays were used on NB AI and AD lysates that were extracted, quantified, and used for analysis. Of the vast list of proteins to validate from the proteomics data, we chose to focus on the proteins that were of most interest to our area of research in terms of epithelial to mesenchymal transition (EMT), stemness, radio-resistance and malignancy based on current literature findings. As such, we selected a number of proteins to validate using Western blot analysis that were reported from the literature to induce proliferation, migration, EMT, radio-resistance and to inhibit apoptosis. Western blot analysis concurred with the protein profiling results confirming CDC2, a proliferative marker, was expressed 2-fold higher in AD compared to AI cells (Fig 2). PARP 1 (DNA repair enzyme), SOX2 (stem marker), HDGFR, MIA3, PDGFRβ, VEGFR and EGFR (tumorigenic proteins affiliated with poor prognosis) as well as RhoA (EMT-driver and metastatic/invasive marker) were all up-regulated (fold change ≥ 1.5) in the AI tumorspheres (Fig 2); an indication of higher malignancy, radio-resistance and EMT in the AI tumorspheres compared to the AD cells.

### AI tumorspheres exhibit significant radio-resistance compared to AD cells

**Fig 3:**
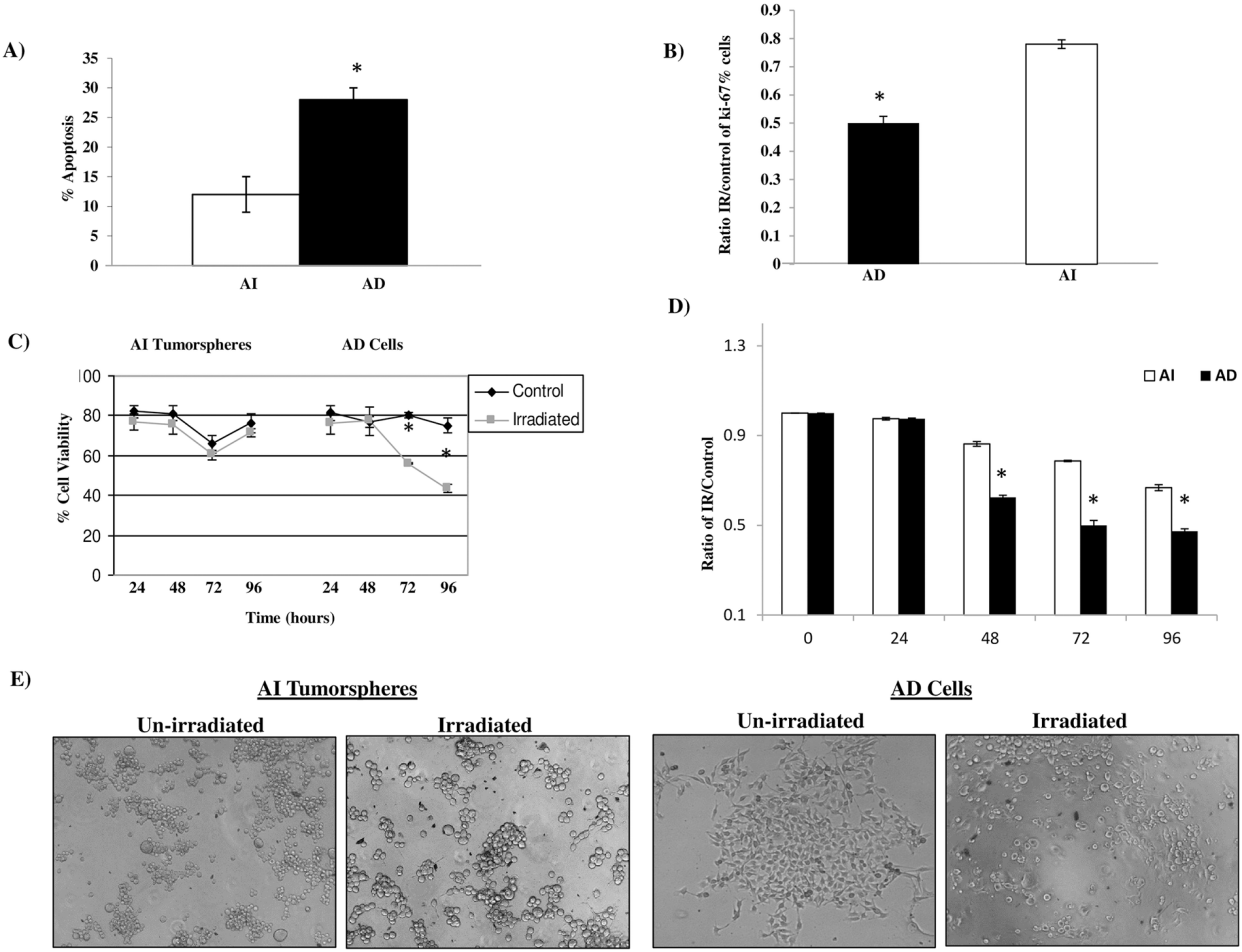
AI tumorspheres exhibit resistance to radio-therapy compared to AD cancer cells. (A) AD cells (black bars) had a significantly (*P < 0.*05*) higher rate of apoptosis 2 days after a single dose (2.5 Gy) of radiation compared to AI tumorspheres (white bars) as assessed using 7AAD/Annexin V staining. (B) AD cells had a more potent reduction (81% to 38%) in the proliferation marker Ki-67 expression than AI tumorspheres (72% to 52%) at 72 h post radiation compared to control cells. The average ratio of Ki-67% irradiated/control cells in the AI tumorspheres was 0.75 whereas that of the AD cells was 0.45, as derived from the mean of 4 independent experiments run in triplicates and presented ±SEM of multiple experiments. (C) AI tumorspheres (left side) exhibited a higher number of viable cells over 96 h post radiation compared to AD cells (right side) as determined by counting viable (non-trypan blue stained) and non-viable (trypan blue stained) cells, used in support of the XTT cell proliferation assay. (D) The average ratio of irradiated/control neuroblastoma AD cells (black bars) and that of AI tumorspheres (white bars) was derived from the absorbance of 4 independent experiments run in triplicates and graphed. The ratio was significantly different (*P < 0.*05*) between the AD cells and AI tumorspheres at 48, 72 and 96 h post radio-therapy(2.5 Gy) indicating the radio-resistant nature of the AI tumorspheres compared to the AD cells. (E) AD cells exhibit increased cell death after radio-therapy compared to AI tumorspheres as shown in the photographic representation of the AI tumorspheres and AD Cells 72 h post radio-therapy revealing more dead cells and cellular debris in the AD cells compared to the AI tumorspheres. * indicates a *P value < 0*.*05*. Results represent the mean ± SEM of multiple experiments.

To test the functional difference between AI tumorspheres and AD cells in terms of response to radio-therapy, we subjected the phenotypically distinct cells to 2.5 Gy radio-therapy and tested their rate of apoptosis, Ki-67 expression, percent viability and proliferation after radio-therapy. Cell apoptosis was measured 48 h post-radio-therapy and revealed AD cells (~26%) to be more sensitive to radio-therapy than AI tumorspheres (~12%) as assessed using a 7AAD/Annexin-V assay which revealed a higher rate of apoptosis in the AD cells compared to AI tumorspheres (Fig 3A). In addition, we observed a more potent decrease in Ki-67 staining in the AD cells (81% to 38%) compared to the AI tumorspheres (72% to 52%) 72 h post radio-therapy (Fig 3B). Cell viability after radio-therapy remained constant in the AI tumorspheres over 96 h whereas there was a decline in percentage of viable cells in the AD population over 96 h post radio-therapy (Fig 3C). Irradiated AD cells were more sensitive to radio-therapy compared to AI tumorspheres as assessed by a greater decrease in the rate of cell proliferation over 96 h compared to their un-irradiated counterparts (Fig 3D). Fig 3E is a representative image of the morphology and cell density in the AI and AD cell populations taken at 72 h post radio-therapy showing less confluent, dying cells in the AD compared to the AI population.

### Significant up-regulation of survivin, PARP1 and CDC2 post radio-therapy

**Fig 4:**
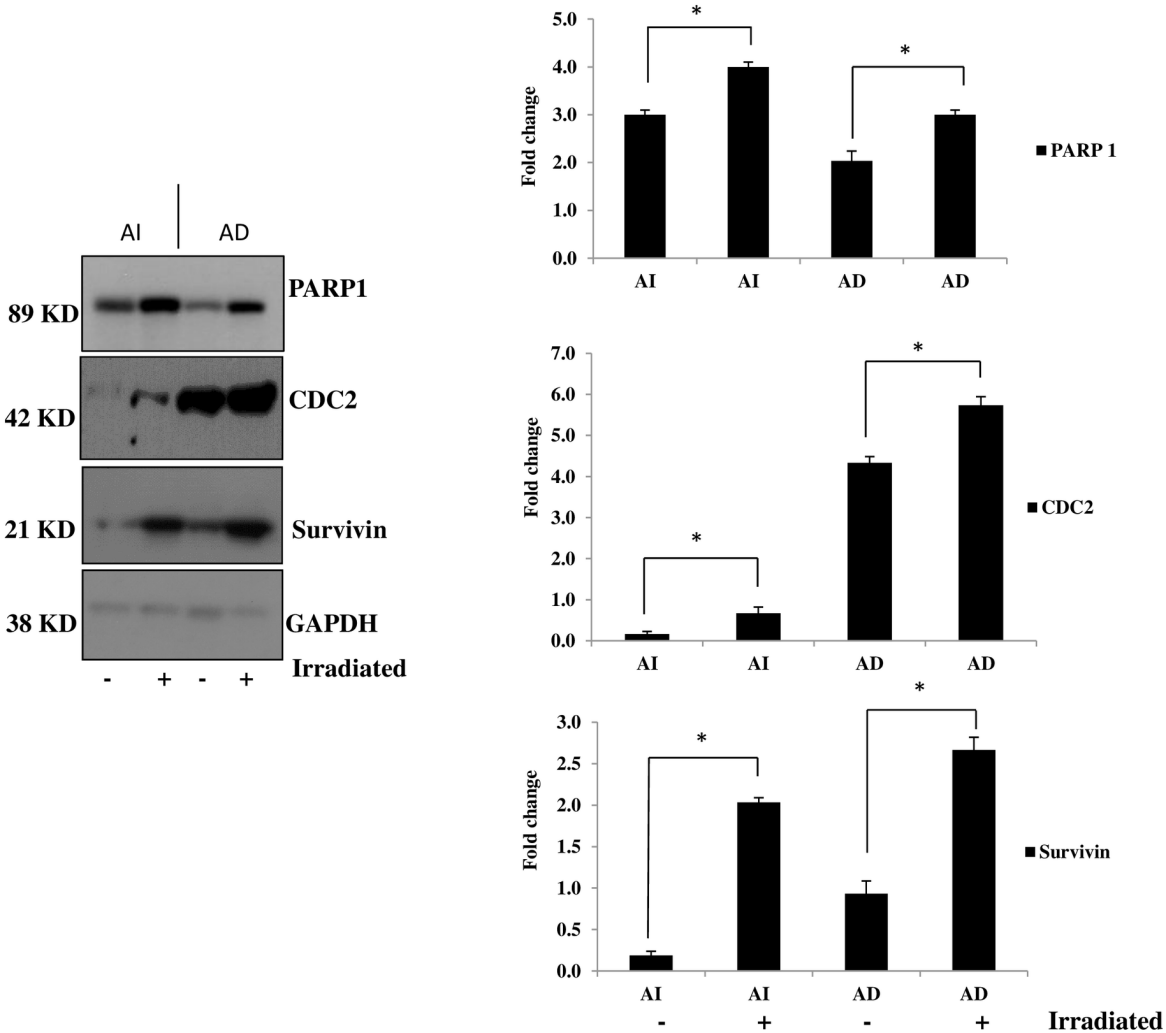
AI tumorspheres and AD cells exhibit significant up-regulation of survivin, PARP 1 and CDC2 post radiation. AI tumorspheres or AD cells were either irradiated (+) (2.5 Gy) or left un-treated (-) and cells were lysed after 48 h of irradiation and proteins separated on a SDS-page gel for Western blot analysis. Survivin was significantly up-regulated after irradiation of both AI tumorspheres and AD cells compared to untreated controls. Furthermore, PARP 1 was significantly up-regulated after irradiation of both AI tumorspheres and AD cells compared to untreated controls. In addition, CDC2, a proliferative marker, which was significantly over-expressed by the AD cells compared to the AI tumorspheres, showed significant up-regulation in both AI tumorspheres and AD cells 48 h post irradiation compared to untreated cells. * indicates a *P value < 0*.*05*. Results represent the mean ± SEM of multiple experiments.

Our next focus was on the radiation-induced protein expression in the AI tumorspheres and AD cells. We chose to focus on the proliferation markers, survivin, CDC2 and the DNA-repair enzyme PARP1 as our targets to examine after radio-therapy in both AI and AD cells. Survivin is a marker for malignancy and is a poor prognostic marker in NB, whereas CDC2 and PARP1 may be providing radio-therapy protection to our cells via DNA repair mechanisms and cell cycle progression. AI tumorspheres and AD cells exposed to a single cycle of 2.5 Gy radiation exhibited a significant up-regulation of survivin, PARP1 and CDC2 48 h post-radio-therapy as determined using Western blot analysis (Fig 4).

### AI tumorspheres exhibit resistance to PDGFRβ inhibition compared to AD cells

**Fig 5:**
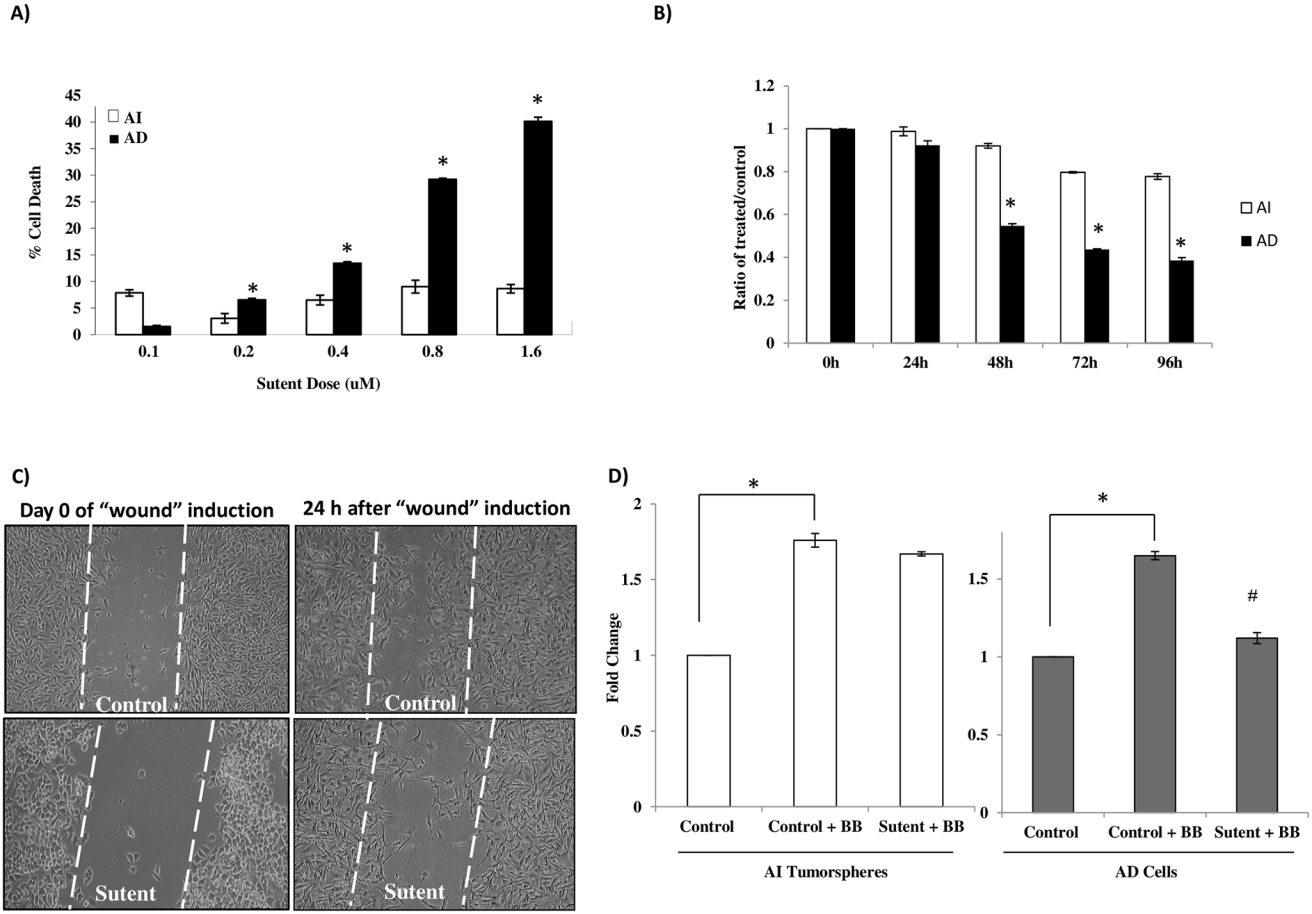
Small molecule inhibition of the PDGFRβ using sutent significantly increases cell death and inhibits cell proliferation and migration in AD cells. (A) NB AI tumorspheres (white bars) exhibited significant resistance to increasing concentrations of sutent treatment compared to un-treated counterparts, as assessed using a cytotoxicity assay after 48 h of treatment, whereas AD cells (black bars) showed a dose-dependent increase in cell death, compared to un-treated counterparts. (B) The average ratio of treated/control absorbance of NB AI tumorspheres and AD cells from four independent experiments run in triplicates was derived and graphed ± SEM. The AI tumorspheres (white bars) were significantly resistant to sutent-induced reduction in cell proliferation compared to their un-treated counterparts over 24, 48, 72 and 96 h post treatment, whereas AD cells (Black bars) showed a marked reduction in cell proliferation after sutent treatment compared to their un-treated counterparts as determined using a WST-1 cell proliferation assay. (C) A “wound-healing” assay was used to assess the PDGF-BB (10 ng) induced cell migration of NB AD cells, treated with sutent for one hour before ‘wound-induction’. Sutent significantly inhibited the AD cells’ ability to migrate into and close the wound after 24 h. (D) A trans-well chemotactic migration chamber was used to measure the PDGF-BB induced cell migration of AD and AI cells that migrated to the underside of the chamber with or without sutent treatment. The average absorbance was represented as a ‘fold-change’ between the groups; unstimulated, untreated cells (Control) were normalized to 1, whereas PDGF-BB induced migration (control + BB) and sutent-induced inhibition of PDGF-BB stimulated migration (sutent + BB) was measured as a fold-change. PDGF-BB stimulation led to a significant (*) increase in AI (white bars) and AD (Grey bars) cell migration compared to their un-stimulated controls. Sutent treatment significantly (#) abrogated (*P < 0*.*05*) the PDGF-BB induced AD cells’ (grey bars) ability to migrate to the lower side of the chamber (Fold-change from 1.65 to 1.2), but had only a slight, but not significant (fold-change from 1.75 to 1.65) effect on the trans-well migration capacity of AI tumorspheres (white bars). * indicates a *P value < 0*.*05*. Results represent the mean ± SEM of multiple experiments.

We next decided to examine the effect of PDGFRβ inhibition of phosphorylation in our AI tumorspheres compared to the AD cells considering the significant over-expression of this receptor as well as EGFR and VEGFR in the AI tumorspheres compared to the AD cells. We tested the effect of PDGFRβ inhibition on cellular cytotoxicity, proliferation, wound-healing and trans-well migration after 0.2μM sutent treatment versus control, vehicle-treated cells. We observed a dose-dependent increase in cytotoxicity in AD cells after sutent treatment that was not observed in the AI tumorspheres (Fig 5A). Similarly, the rate of cell proliferation over 96 h was significantly inhibited in the AD cells compared to their un-treated counterparts, (Fig 5B), whereas AI tumorspheres only exhibited a subtle decrease in the rate of cell proliferation with sutent treatment, compared to their sutent-treated counterparts. Cell migration was assessed using a “wound-healing” scratch assay in the AD cells, which was inhibited after sutent treatment compared to un-treated controls (Fig 5C). The floating AI tumorspheres as well as the AD cells were tested for migration using the trans-well chemotactic cell migration chamber. Sutent treatment inhibited the trans-well chemotactic migration of the AD cells (grey bars), compared to their un-treated counterparts, but had no effect on the AI tumorspheres, compared to their un-treated counterparts (white bars) (Fig 5D).

### Sutent inhibits the PFDGRβ phosphorylation-induced transactivation of EGFR and VEGFR

To determine whether the PDGFRβ was active in the AI tumorspheres versus the AD cells, we stimulated the receptor with the PDGF-BB ligand (5 mins) and assayed the phosphorylation of PDGFRβ, Akt and Erk in the AI tumorspheres compared to the AD cells. PDGFRβ stimulation indeed led to phosphorylation of PDGFRβ, Akt, and Erk in the AI tumorspheres (S2 Fig). No significant activity of the PDGFRβ was detected in AD cells, however, we observed a slight increase in AKT and ERK1/2 phosphorylation in them. Up-regulation of the PDGFRβ expression in the AI tumorspheres and its robust PDGF-BB induced activity in its downstream signaling cascade is another indication of a more malignant phenotype of these cells.

**Fig 6:**
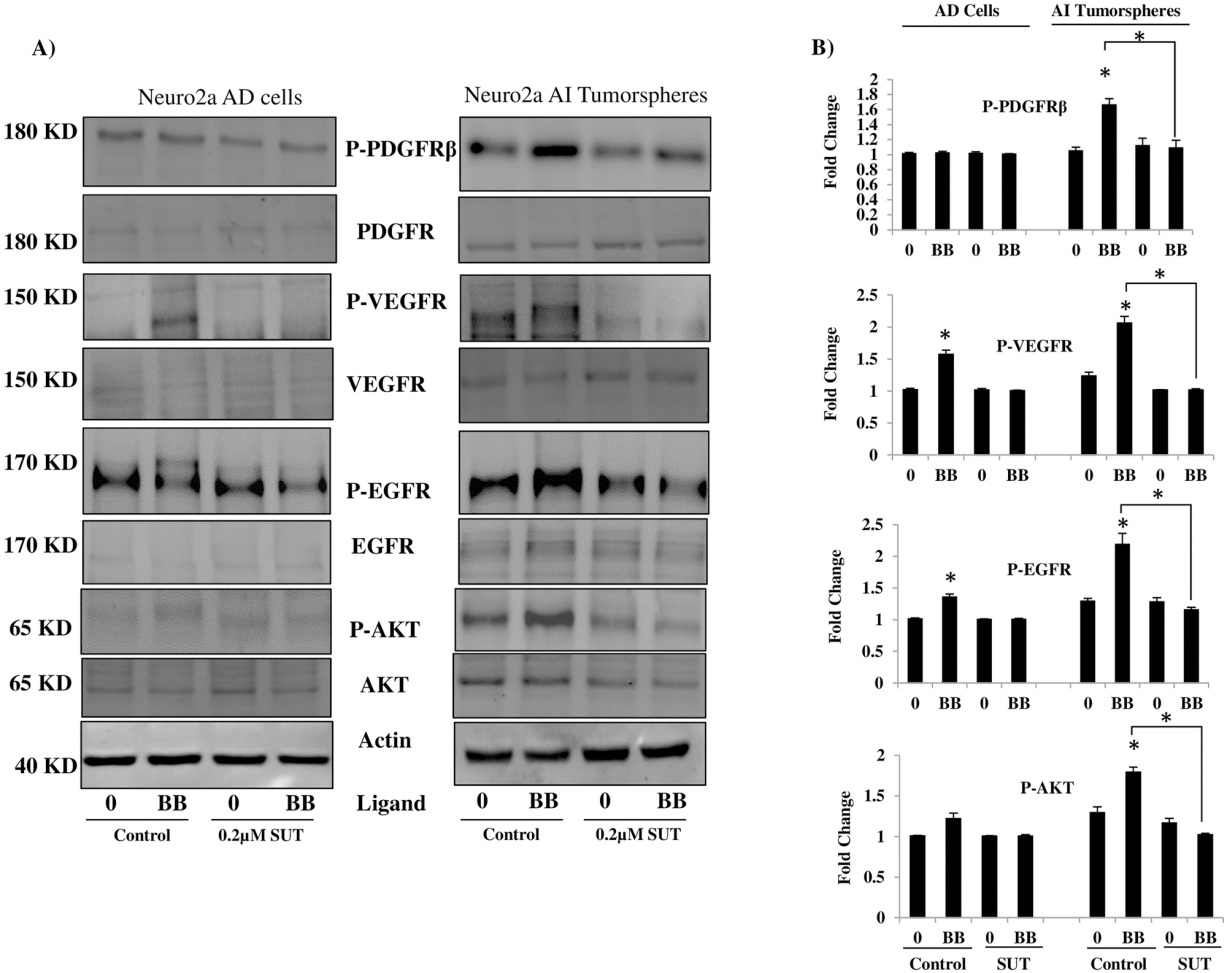
Sutent inhibits the phosphorylation of the PDGFRβ and EGFR/VEGFR trans-activation. (A) Western blot analysis of AI tumorspheres compared to NB AD cells showed significant phosphorylation (B) of PDGFRβ and the downstream signaling cascade (Akt) after 5 mins stimulation with 10 ng PDGF-BB in AI tumorspheres, which was significantly inhibited after 1 h of 0.2 μM sutent pre-treatment. AD cells were less-affected by PDGF-BB stimulation compared to the AI tumorspheres (A and B). In addition, AD and more potently AI cells stimulated with PDGF-BB for 5 minutes exhibited trans-activation of both EGFR and VEGFR that was abolished with sutent pre-treatment for 1 h (A and B). * indicates a *P value < 0*.*05*. Results represent the mean ± SEM of multiple experiments.

We next aimed to test the effect of sutent on inhibition of the PDGFRβ signaling cascade. Sutent treatment (1 h) profoundly inhibited the PDGFRβ signaling cascade as indicated by reduced levels of Akt, Erk and PDGFRβ phosphorylation in AI tumorspheres (S2 Fig). Interestingly, very little phosphorylation of Erk and even less of Akt was observed in the AD cells with PDGF-BB stimulation despite the absence of PDGFRβ expression and the lack of PDGFRβ phosphorylation in these cells. This Akt and Erk phosphorylation was abrogated after sutent treatment (S2 Fig) in the AD cells. Surprisingly, cell survival was inhibited in the AD cells following sutent treatment, but AI tumorsphere survival was not affected despite the robust expression of PDGFRβ. We speculate that the activation of the downstream targets of PDGFRβ in the AD cells is due to cross-talk between this receptor and the neighboring EGFR, a finding we previously reported to occur in childhood medulloblastoma cells. As expected, we observed this cross-talk to occur in our Neuro2a AD cells, where a PDGF-BB induced trans-activation of EGFR was abrogated after sutent treatment (Fig 6A). In the AI tumorspheres, this trans-activation was also observed to occur even more potently compared to the AD cells, which was abrogated with sutent inhibition of PDGFRβ. We next asked whether there is more cross-talk between the PDGFRβ and other neighboring RTKs such as VEGFR and whether this cross-talk is significantly different between the AD cells and AI tumorspheres and if sutent is capable of abrogating this cross-talk and the downstream signaling cascade. To our surprise, we observed a PDGF-BB induced trans-activation of VEGFR in both AD cells and more potently the AI tumorspheres, which was also abrogated with sutent treatment.

### Survivin knock-down in addition to RTK inhibition significantly enhances radio-resistance and inhibits cell proliferation in AI tumorspheres

**Fig 7:**
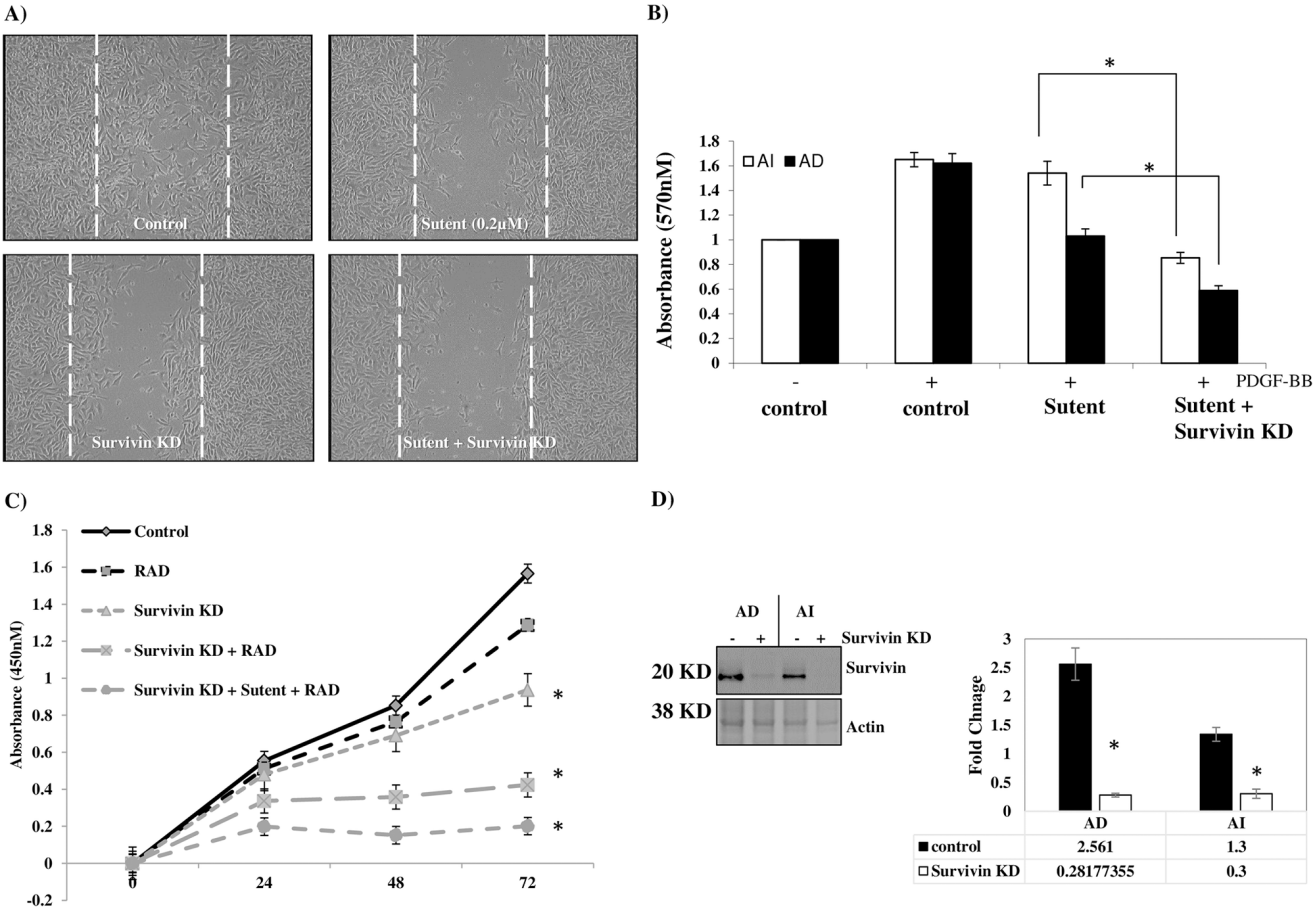
Sutent combined with survivin knock-down exhibits added effect on inhibition of cell migration and invasion and enhanced AI radio-sensitivity. (A) NB AD cells treated with 0.2 μM sutent plus survivin knock-down exhibited an added effect on the inhibition of cell migration as assessed with a ‘wound-healing’ assay compared to either treatment alone. (B) AI tumorspheres (white bars) treated with a combination of sutent (0.2 μM) and survivin knock-down exhibited a significant reduction in PDGF-BB (+) mediated cell invasion capacity compared to sutent treatment alone or untreated controls. AD cells (black bars) treated with sutent alone exhibited inhibition in PDGF-BB (+) mediated cell invasion, which was also enhanced in combination with survivin knock-down. (C) Survivin knock-down in AI tumorspheres increased radio-sensitivity when combined with sutent treatment by exhibiting an added effect on inhibition of cell proliferation after radio-therapy compared to survivin knock-down alone. (D) Western blot analysis confirms significant knock-down of survivin protein expression in neuro2a AD cells and AI tumorspheres measured after 48 h of siRNA transfection. * indicates a *P value < 0*.*05*. Results represent the mean ± SEM of multiple experiments.

As previously reported, the AI tumorspheres were resistant to radio-therapy compared to the AD cells. After noticing the significant up-regulation of survivin, PARP and CDC2 in both cell types after radio-therapy, we were curious about the roles played by these proteins in radio-resistance of the AI tumorspheres. Especially interesting was the significant up-regulation of the tumorigenic and proliferative marker survivin, which has been previously reported to confer poor clinical outcomes in children with neuroblastoma [18]. We believe that the AI tumorspheres use survivin as well as the other tumorigenic proteins that they over-express compared to AD cells to evade radio-therapy. We therefor used siRNA to knock-down the protein expression (Fig 7D) of survivin in the AI tumorspheres and AD cells and subsequently investigated the added effect of combining survivin knock-down with sutent treatment on cell migration and invasion as well as sensitivity to radio-therapy.

The capacity of AD cells to migrate into and close a wound in the ‘wound-healing’ scratch assay was exaggerated when survivin knock-down was combined with sutent treatment in these cells (Fig 7A). Cell invasion in the presence of PDGF-BB chemotactic factor was assessed in both AI and AD populations with either sutent treatment alone or in combination with survivin knock-down. While sutent treatment alone did not significantly inhibit cell invasion in the AI tumorspheres, when combined with survivin knock-down, we observed a significant reduction in PDBG-BB induced cell invasion of the AI tumorspheres compared to sutent treatment alone (Fig 7B).

To determine the effect of dual sutent treatment and survivin knock-down in the AI tumorspheres on radio-therapy sensitivity, we assessed the rate of cell proliferation in the AI tumorspheres over 72 h post radio-therapy in cells treated with sutent alone or in combination with survivin knock-down. Survivin knock-down led to significant inhibition in cell proliferation of AI tumorspheres starting from 48 h post knock-down, but this effect was exaggerated and evidenced as early as 24 h when combined with radio-therapy (2.5 Gy) and even more exaggerated in combination with sutent (0.2 μM) treatment (Fig 7C).

### Long-term Sutent treatment (48 h) inhibits the protein expression of PFDGRβ, MYCN, SOX2 and Survivin and abrogates tumorsphere self-renewal potential in AI tumorspheres

**Fig 8:**
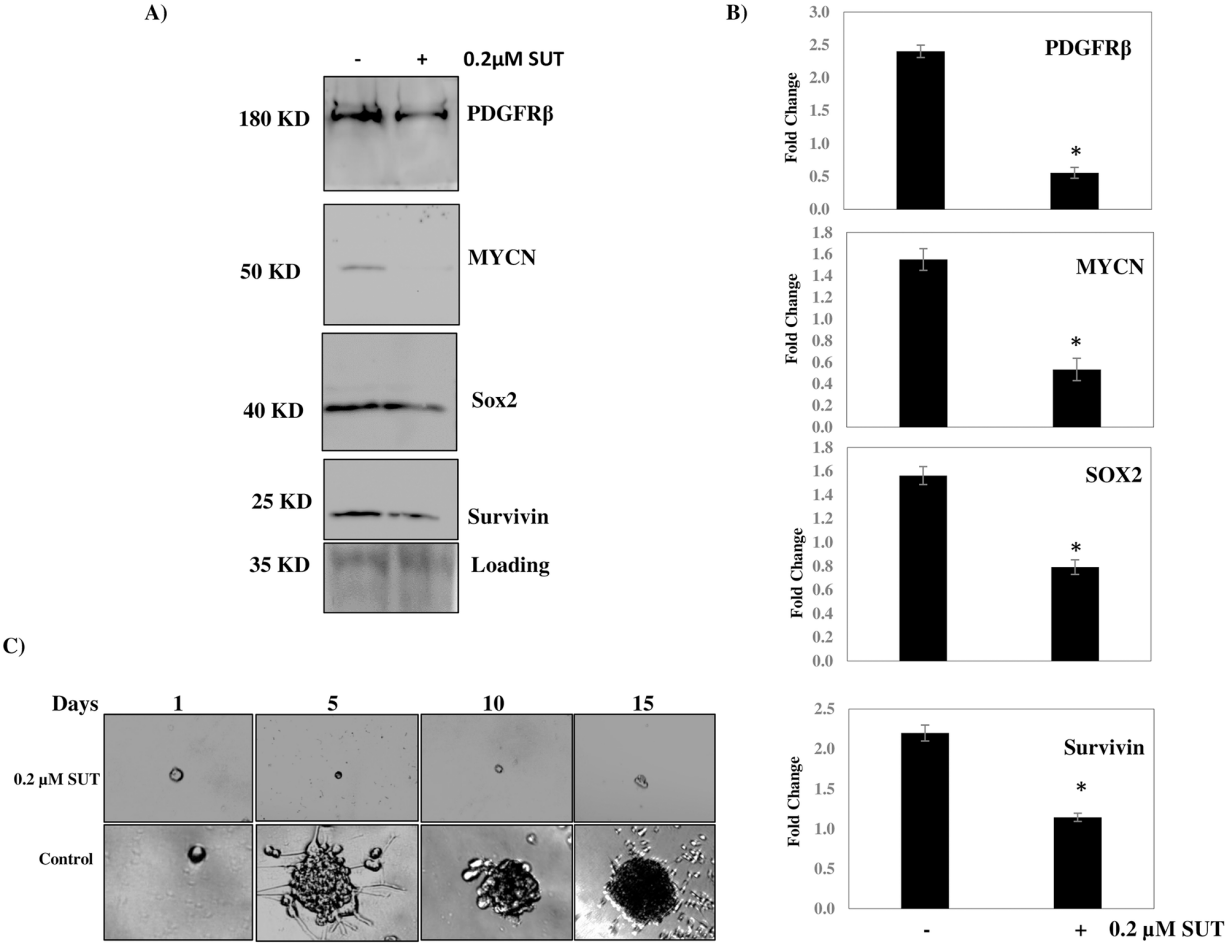
Long-term sutent treatment in AI tumorspheres inhibits tumorigenic protein expression and tumorsphere self-renewal. AI tumorspheres were treated with 0.2 μM sutent for 48 h and then lysed for Western blot analysis. A) Western blot images of representative experiments illustrating the reduced expression of PDGFRβ, MYCN, SOX2 and Survivin after 48 h sutent treatment. B) Densitometric analysis of multiple Western blot experiments showing statistically significant reduction in the protein expression of the above mentioned proteins. C) Micro-images of tumorsphere self-renewal capacity over 15-days, inhibited with 0.2 μM sutent treatment. * indicates a *P value < 0*.*05*. Results represent the mean ± SEM of multiple experiments.

To determine the effect of long-term sutent treatment in our AI tumorspheres, we treated the tumorspheres with either vehicle or 0.2 μM sutent for 48 h and then lysed the cells for Western blot analysis. We looked at the long-term effect of sutent treatment on the protein expression of PDGFRβ, MYCN, SOX2 and survivin. These proteins are reported to drive tumorigenicity and stemness maintenance in various tumors. There was a significant *(P < 0*.*05)* reduction in the expression of these proteins after 48 h sutent treatment compared to vehicle treated AI tumorspheres (Fig 8A and 8B). In addition, a tumorsphere self-renewal assay was used to determine whether sutent inhibited self-renewal capacity in our AI tumorspheres. Tumorsphere self-renewal over 15-days was significantly inhibited in the AI tumorspheres with 0.2 μM sutent treatment compared to vehicle-treated counterparts (Fig 8C).

## Discussion

Recurrent neuroblastoma, regardless of advances in treatment options, remains one of the deadliest childhood tumors with poor prognosis and resistance to therapy [19]. Identifying the specific proteins and/or pathways responsible for treatment evasion and enhanced malignancy of recurrent disease is of utmost importance in order to tailor targeted therapies that eradicate the most malignant, treatment resistant cells within the bulk tumor. In order to define specific characteristics and molecular components of the adhered, AD cells and non-adhered AI tumorspheres, we characterized the proteomic profile of these cells and compared them to each other. The neuroblastoma AI tumorspheres exhibited significant up-regulation or exclusive expression of proteins that are associated with anchorage-independent growth, malignancy and epithelial-to-mesenchymal transition compared to the AD cells.

Most notable was the significant over-expression of RhoA, MIA3, PARP1 [20] and HDGF [21] in the NB AI tumorspheres compared to the AD cells, that are associated with increased metastases, epithelial to mesenchymal transition, and malignancy. Furthermore, AI tumorspheres exhibited significant up-regulation of proteins, compared to AD cells, that constituted four general pathways: i) cellular function and maintenance, and carbohydrate metabolism, ii) lipid and energy metabolism, iii) tissue morphology, and iv) cellular assembly and organization. Of particular significance is the up-regulation of proteins involved in small molecule biochemistry (indicating high metabolic activity), RNA post translational modification, and energy production [22] in the AI tumorspheres compared to the AD cells.

Another very interesting protein that showed up-regulation (2 fold, *P < 0*.*05*) in AI compared to AD cells is Serum-Deprivation Response Protein (SDRP). SDRP is known to be a substrate for protein kinase C phosphorylation and becomes up-regulated in serum-starved cells [23]. Not only was SDRP up-regulated in the AI compared to the AD cells, but protein kinase C and casein kinase substrate in neuron (PACSIN2) was also found to be exclusively expressed in our AI compared to the AD cells. Protein kinase C [24] and PACSIN2 [25] are reported to be involved in epithelial to mesenchymal transition of cancer stem-like cells, proliferation and survival of cancer stem-like cells [26] and the aggressive nature of many cancers. These two proteins may be working together in our AI cells to induce their stem-like phenotype and treatment evasive nature compared to the AD counterparts.

Furthermore, our AI tumorspheres exclusively expressed proteins associated with anchorage-independent growth or stem-cell characteristics, including ROCK II and Anxa2, respectively. In addition, proteins associated with increased invasion and radio-resistance (Ctsd and MTCO2, respectively) were also exclusively expressed by the AI tumorspheres. Other reports have confirmed that down-regulation of ROCK 1 and ROCK 2 induced a significant inhibition in anchorage-independent growth and invasion of non-small cell lung carcinoma [27]. Moreover, MTCO2 and Anxa2 were reported to be associated with radio-resistance and as biomarkers of cancer stem cells, respectively [28, 29, 30]. These findings support the hypothesis that our cells exclusively express proteins that may govern their malignant, treatment-resistant, anchorage-independent phenotype when subjected to an unfavorable culture environment. Furthermore, our AI tumorspheres showed a > 2-fold up-regulation of other proteins that are associated with an epithelial-to-mesenchymal transition (Tpi1, Pgk1, Hspa8, Hnrnpk, Nampt and Rho A) compared to the AD cells which could further explain the anchorage-independent growth and malignant properties of this population [28, 31].

Along with the mentioned proteins, we also found with Western blot analysis the AI tumorspheres to significantly up-regulate other highly tumorigenic proteins involved in aggressive tumor behavior, including PDGFRβ, VEGFR2 and EGFR compared to the AD cells. The PDGFRβ signaling cascade is highly tumorigenic, contributing to pro-survival, pro-migratory and anti-apoptotic behaviors in a variety of tumors [32, 33, 34, 35]. To determine whether the PDGFRβ was active in the AI tumorspheres we investigated the PDGF-BB ligand-dependent phosphorylation of the receptor and also assayed the phosphorylation of its downstream signaling cascade, namely Akt and Erk in the AI tumorspheres compared to the AD cells. Indeed, PDGFRβ stimulation led to phosphorylation of PDGFRβ, Akt, and Erk in AI tumorspheres more potently than in the AD cells (S2 Fig). The significant up-regulation of the PDGFRβ cascade and its higher level of PDGF-BB induced activation in the AI tumorspheres compared to the AD cells is another indication of a more malignant molecular phenotype the AI tumorspheres possess. Sunitinib malate (sutent^®^), the small molecule inhibitor that targets receptor tyrosine kinases [36] profoundly inhibited PDGFRβ activation and downstream signaling cascade as indicated by inhibition of Akt and Erk phosphorylation in both the AI tumorspheres and the AD cells. Interestingly, cell survival, cytotoxicity, proliferation and migration were all significantly inhibited in the AD cells following sutent treatment, but AI tumorspheres were resistant to this drug despite the robust expression of PDGFRβ and the sutent-induced inhibition of its signaling cascade. This observation suggests that AI tumorspheres have redundant survival and proliferative/migratory pathways that do not rely solely on the expression or activation of the PDGFRβ for survival. Previously, we reported on the trans-activation of the EGFR by PDGF-BB in childhood medulloblastoma cells. This EGFR trans-activation was mediated through the PDGF-BB-induced activation of the PDGFRβ, which was abolished after siRNA knock-down of the PDGFRβ or its chemical inhibition with sutent [37, 38]. We investigated whether the NB AD cells and AI tumorspheres also exhibited this trans-activation of EGFR after PDGF-BB induced PDGFRβ phosphorylation. In fact, we also found that after 5 mins of PDGF-BB stimulation of PDGFRβ there was significant trans-activation of EGFR as well as VEGFR, which was significantly inhibited with sutent pre-treatment. The PDGF-BB induced trans-activation of EGFR and VEGFR was more potent in our NB AI tumorspheres compared to the AD cells. We speculate that the up-regulation of PDGFRβ, VEGR and EGFR as well as the more potent trans-activation of these receptors among the other tumorigenic proteins reported to be over-expressed here in the AI tumorspheres is an example of the cellular response to a “stressful”, sub-optimal culture environment in which the cells undergo a phenotypic transition and molecular adaptation rendering them highly tumorigenic with enhanced pro-survival, anti-apoptotic and pro-migratory behavior.

In support of this notion, various studies have recently reported on the factors that may render these phenotypic changes in cancer cells including stem-like cell maintenance, de-differentiation of non-stem cancer cells and angiogenic potential after exposure to current therapeutic modalities including radio- and chemo-therapy as well as small molecule, targeted therapy. It is hypothesized that hypoxia within the bulk tumors and disruption of the nutrient reservoirs along with changes in intra-tumoral pH, and cytotoxic insults all seem to drive these ‘stressed’ cancer cells into the phenotypic, malignant transformation of cancer stem-like cells [39].

Some of the players found to be affiliated with EMT and cancer stem-like cell maintenance include the PI3K/AKT pathway [40], RhoA [41], VEGFR [42], SOX2 [43] and Wnt/β-catenin and SHH pathways [44]. It is not surprising that these pathways present highly attractive targets for therapeutic intervention seeing how they may be involved in the EMT, treatment resistance and plasticity of cancer stem-like cells. We found the PI3K/AKT pathway over-activated and SOX2, RhoA and VEGFR over-expressed in our AI tumorspheres and thus we speculate that this may be one of the underlying mechanisms that is fueling this malignant, treatment-resistant, stem-like behavior in our AI tumorspheres.

To determine the effect of long-term sutent treatment on our AI tumorspheres, we treated the cells with 0.2 μM sutent for 48 h and observed a significant reduction in the protein expression of PDGFRβ, SOX2, Survivin and MYCN. In addition, we assessed tumorsphere self-renewal potential in the AI tumorspheres with and without sutent treatment over 15 days and found a significant reduction in the tumorsphere self-renewal potential of these cells with sutent treatment. We therefor believe that sutent may play anti-self-renewal and anti-malignancy roles in neuroblastoma tumorspheres by inhibiting the protein expression of the stemness-maintenance drivers, SOX2 and PDGFRβ as well as the malignant, proliferative markers MYCN and survivin.

Care needs to be taken, though, when targeting some of these pathways. As we have seen from various reports, targeting the SHH pathway for inhibition using vismodegib led to increased frequency of quiescent Sox2^+^ medulloblastoma cells in the Ptch^+/−^ mouse models [45]. Furthermore, Hambardzumyan *et al*. identified a stem cell population with enhanced Nestin^+^ expression in the perivascular niche of different post-irradiated Shh-medulloblastoma mouse models. The authors reported that these cells may re-enter the cell cycle 72 h after radio-therapy via a radiation-induced loss of PTEN expression. The loss of PTEN was followed by subsequent activation of the PI3K/Akt pathway, which was reversed using the PI3K/AKT inhibitor perifosine [41], thereby implicating the PI3K/AKT pathway as a key player in the radio-resistance observed in this model of cancer stem-like cells. This phenomenon highlights the dynamic processes that drive cancer stem-like cell propagation particularly after standard therapeutic applications that may enhance micro-environmental factors within the bulk tumors that drive this plasticity of cancer cells towards an unfavorable, malignant and treatment resistant phenotype.

To determine whether our AI tumorspheres were resistant to radio-therapy and small molecule inhibition of the PDGFRβ activity, compared to the AD cells, we subjected them to 2.5 Gy of radiation or 0.2 μM sutent treatment and measured their bio-function activity (proliferation, apoptosis, cytotoxicity, migration) after treatment. While AI tumorspheres showed significant resistance to these therapies compared to AD cells, both AD cells and AI tumorspheres exhibited significant up-regulation of the proliferative markers, survivin and CDC2 and the DNA repair enzyme, PARP1 48 h after radio-therapy. Survivin is an anti-apoptotic protein, whose expression in neuroblastoma has been associated with poor prognosis and mortality [46]. PARP 1 is involved in repair of single-stranded DNA (ssDNA) breaks and assures that these breaks are repaired before the cells can undergo replication. Previous reports have implicated the role of PARP1 in cancer generally [47, 48] and neuroblastoma specifically [49, 50]. The observed significant up-regulation of survivin, CDC2 and PARP 1 in our cells after radio-therapy indicates that the cells may be attempting to evade this treatment modality and remain viable. However, since both the AI tumorspheres and AD cells significantly up-regulated these markers after radio-therapy, whereas only the AI tumorspheres were resistant to this therapy, we conclude that these factors are not the sole mediators responsible for radio-resistance in the AI tumorspheres. We speculate that the significant or exclusive over-expression of the other tumorigenic proteins identified by the proteomics analysis in the AI tumorspheres compared to the AD cells may be what equips them with that added malignant property of radio-resistance. It is of utmost importance to investigate the pathways governing these proteins and their interrelationships in order to devise a sound therapeutic approach that would radio-sensitize the AI tumorspheres and hinder their ability to withstand this conventional therapy.

Considering the negative correlation between survivin expression and the prognosis of children with neuroblastoma [51], we decided to target it for transcriptional knock-down and determine whether this would affect the radio-therapy response in the AI tumorspheres. We found that survivin knock-down not only enhanced the sutent-induced inhibition in cell migration and invasion in the AD cells, but also inhibited cell invasion in the AI tumorspheres when combined with sutent treatment. In addition, survivin knock-down led to a significant inhibition of cell proliferation in the AI tumorspheres and when combined with radio-therapy, an added effect was observed. Furthermore, survivin knock-down together with sutent treatment and radio-therapy exerted an even more potent inhibition on cell proliferation in the AI tumorspheres. This highlights the importance of a multimodality approach to eradicate the most aggressive, sub-population of therapy-resistant, stem-like cancer cells. Others have also reported on the importance of combining therapies that target various, multimodalities in cancer to eradicate the stubborn, treatment-resistant stem-like cells. We also observed a significant reduction in the protein expression of the tumorigenic (PDGFRβ and Survivin) and stemness-driving proteins (SOX2 and MYCN) after long-term sutent treatment in our AI tumorspheres. We therefor speculate that this may be part of the mechanism by which combining sutent treatment with radio-therapy or survivin knock-down leads to an added effect on inhibition of cell proliferation and migration.

We previously reported on the radio-sensitivity effect and inhibition of tumorsphere self-renewal [52] of targeting a stem marker, L1-CAM [53], for knock-down in the human MycN-amplified IMR-32 neuroblastoma cells. Reported by others to be highly affiliated with stemness maintenance, invasive potential and malignancy of glioblastoma stem cells [54], L1-CAM is understandably an attractive target for therapeutic intervention. In our findings, combining L1-CAM knock-down with sutent treatment enhanced the radio-therapy response in our cells, further supporting the importance of combining therapies which target differing pathways that govern tumorigenic behavior in order to achieve better anti-cancer activity.

Other reports in pediatric brain cancers further confirm that combination therapies have shown great promise in eradicating aggressive cancers including, a synergistic anti-tumor effect of administering the histone deacetylase inhibitor, Panobinostat, together with GSKJ4 (a potent selective jumonji H3K27 demethylase inhibitor) in H3.3K27M mutant DIPG cells [55]. Yet others have demonstrated the synergistic effect on inhibition of cell proliferation and colony formation in medulloblastoma cells when treatment consisted of the pan-Aurora kinase inhibitor (AMG 900) together with the histone deacetylase inhibitor (SaHa) [56]. Such investigations further signify the possible clinical therapeutic efficacy of combinatorial therapy aimed at epigenetic modulators in pediatric brain cancers.

Our proteomics profile of the two phenotypically and molecularly distinct cell populations within the murine Neuro2a cells have expanded the possible molecular drivers that may be responsible for the aggressive nature of this deadly disease. The exclusively or significantly over-expressed tumorigenic proteins identified in the AI tumorspheres compared to the AD cells shed some insight on the possible mechanisms behind the treatment resistance, self-renewal, invasive and malignant potential of these cells. Future investigations targeting these pathways could further elucidate the treatment-evasive nature and malignant properties of the resistant neuroblastoma AI tumorsphere phenotype and provide opportunities for novel therapeutic approaches.

## Supporting information

S1 Fig Ingenuity Pathway Analysis identifies ERBB2 pathway up-regulation in AI Tumorspheres. Pathway exploring analysis [Ingenuity Pathway Analysis (IPA]) showed up-regulation (≥ 2-fold) of downstream targets of ERBB2 (including: TPD52L2, SDPR, ROCK2, POLD1, PRPF40A, NUP214, DDB1 and SH3BGRL3) in the AI tumorspheres compared to the AD cells.
(TIF)

S2 Fig Sutent inhibits PDGFRβ phosphorylation and down-stream signaling cascade in AI tumorspheres and AD cells. A) Western blot images illustrating the phosphorylation of the PDGFRβ and downstream signaling cascade and the inhibitory effect of Sutent treatment (0.2 μM) for 1 h in the AI tumorspheres and AD cells. B) Densitometric analysis reveals a significant reduction in the PDGFR-BB induced phosphorylation of PDGFRβ, AKT and ERK1/2 with sutent treatment in AI tumorspheres and a reduction of the slight PDGF-BB induced phosphorylation of AKT and ERK1/2 in the AD cells.
(TIF)

S3 Fig Western blot validations of Fig 2 including positive control. The targets validated in Fig 2 are shown here with HeLa whole cell lysates as a positive control for the antibody detection.
(TIF)

S1 Table Proteins differentially expressed between AD cells and AI tumorspheres. List of SP ID, short name and sequence name of the proteins differentially expressed between the AI tumorspheres and AD cells with the spectral counts in the AD cells and AI tumorspheres indicated in the last two columns.
(DOC)

S2 Table Proteins shared between AI and AD cells. List of SP ID, short name and sequence name of the proteins shared between the AI tumorspheres and AD cells.
(XLSX)

S3 Table List of proteins identified, detected spectral counts and calculated fold changes based on spectral counts. This table represents the raw data as derived from the proteomics analysis including the spectral counts (SC) for each peptide and the fold changes (FC) calculated based on these counts.
(XLS)
