# Supplementary figures and images for: Molecular and functional analysis of anchorage independent, treatment-evasive neuroblastoma tumorspheres with enhanced malignant properties: A possible explanation for radio-therapy resistance
Source: PLoS One. 2018 Jan 3;13(1):e0189711. doi: 10.1371/journal.pone.0189711 (PMC5751995; doi:10.1371/journal.pone.0189711)

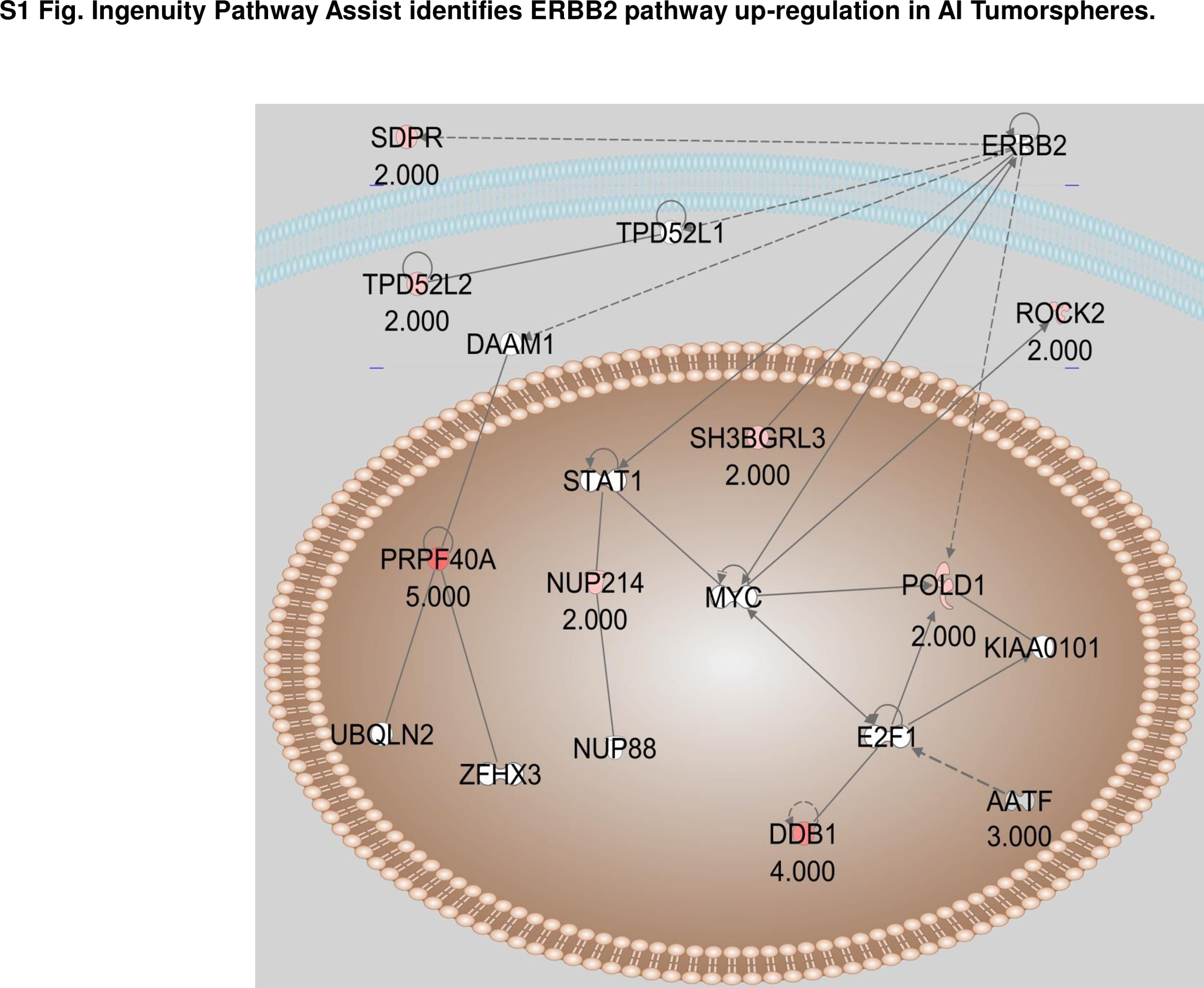

Supplement: S1 Fig — Pathway exploring analysis [Ingenuity Pathway Analysis (IPA]) showed up-regulation (≥ 2-fold) of downstream targets of ERBB2 (including: TPD52L2, SDPR, ROCK2, POLD1, PRPF40A, NUP214, DDB1 and SH3BGRL3) in the AI tumorspheres compared to the AD cells. (TIF) [file pone.0189711.s001.tif]

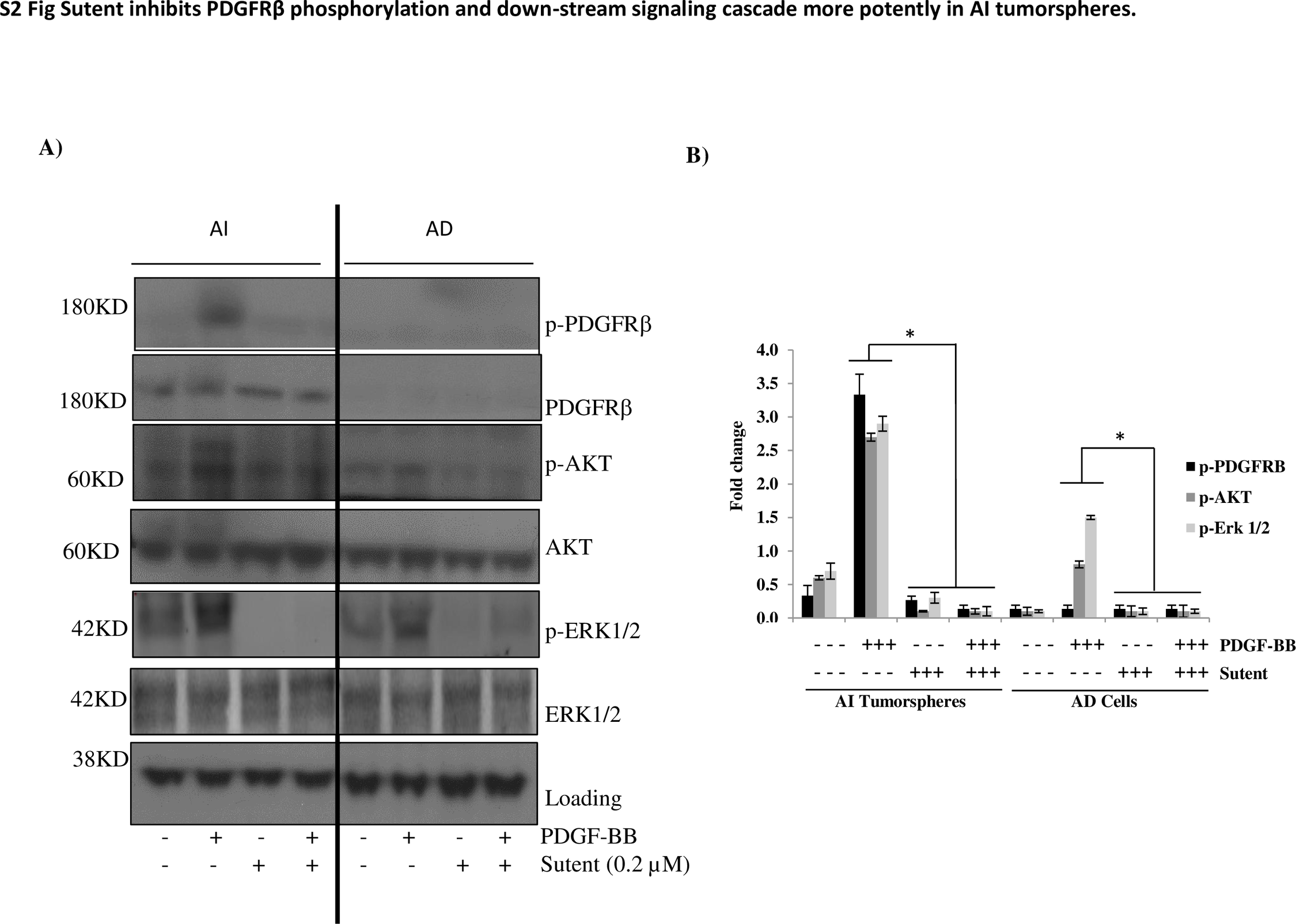

Supplement: S2 Fig — A) Western blot images illustrating the phosphorylation of the PDGFRβ and downstream signaling cascade and the inhibitory effect of Sutent treatment (0.2 μM) for 1 h in the AI tumorspheres and AD cells. B) Densitometric analysis reveals a significant reduction in the PDGFR-BB induced phosphorylation of PDGFRβ, AKT and ERK1/2 with sutent treatment in AI tumorspheres and a reduction of the slight PDGF-BB induced phosphorylation of AKT and ERK1/2 in the AD cells. (TIF) [file pone.0189711.s002.tif]

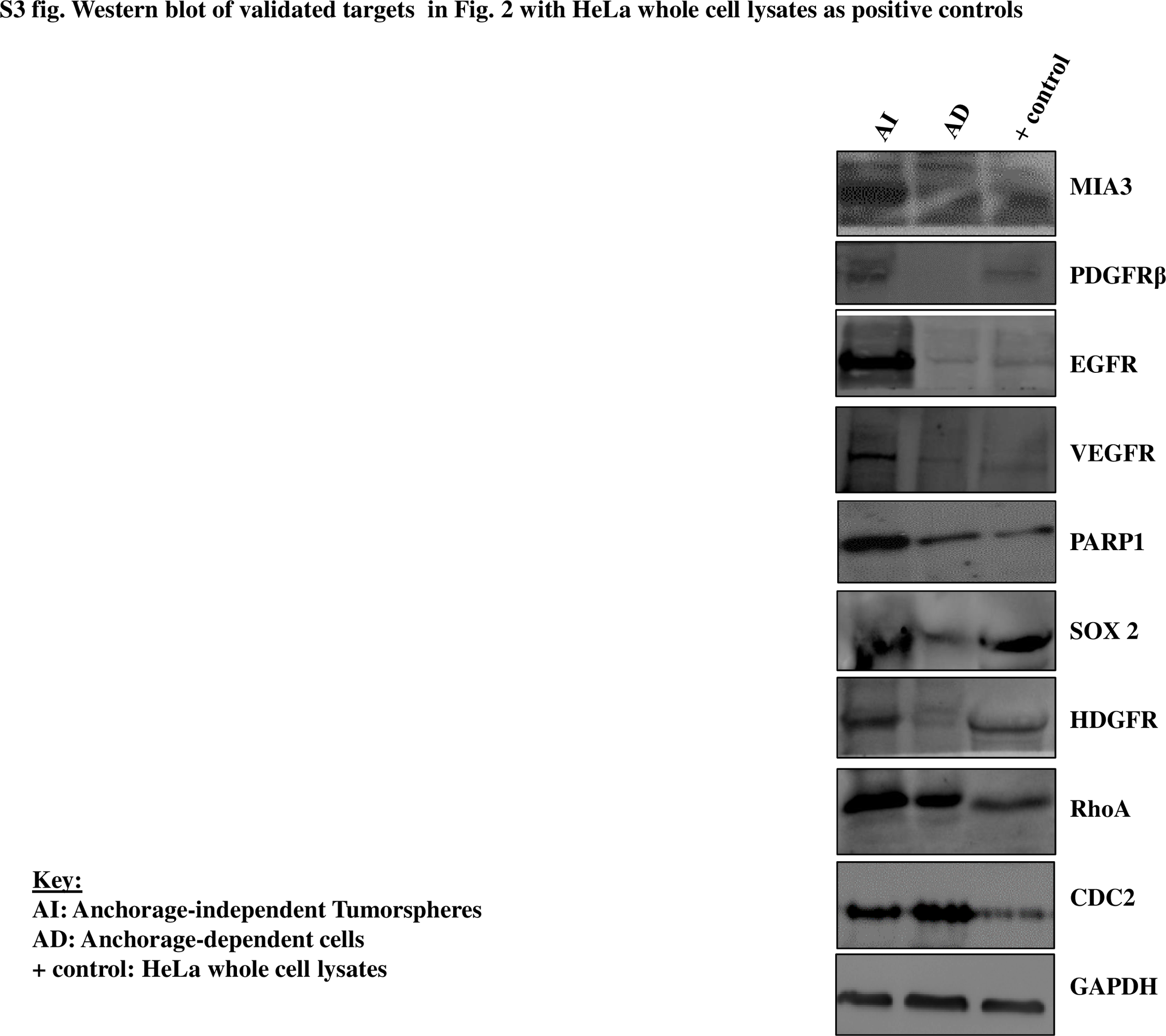

Supplement: S3 Fig — The targets validated in Fig 2 are shown here with HeLa whole cell lysates as a positive control for the antibody detection. (TIF) [file pone.0189711.s003.tif]
