# Supplementary material for: Molecular and functional analysis of anchorage independent, treatment-evasive neuroblastoma tumorspheres with enhanced malignant properties: A possible explanation for radio-therapy resistance
Source: PLoS One. 2018 Jan 3;13(1):e0189711. doi: 10.1371/journal.pone.0189711 (PMC5751995; doi:10.1371/journal.pone.0189711)
Supplement: S1 Table — List of SP ID, short name and sequence name of the proteins differentially expressed between the AI tumorspheres and AD cells with the spectral counts in the AD cells and AI tumorspheres indicated in the last two columns. (DOC) [file pone.0189711.s004.doc]

**S1 Table: Proteins differentially expressed between AI tumorspheres and AD cells**

|  |
| --- |

| **SP ID** | **Short Name** | **Sequence Name** | **AD Normalized SC** | **AI Normalized SC** |
| --- | --- | --- | --- | --- |
| P16546 | Sptan1 PE | Spectrin alpha chain, brain OS | 42.33 | 52 |
| Q8VDD5 | Myh9 PE | Myosin-9 OS | 63.5 | 77 |
| P19096 | Fasn PE | Fatty acid synthase OS | 78.62 | 104 |
| Q62261 | Sptbn1 PE | Spectrin beta chain, brain 1 OS | 31.25 | 35 |
| P20029 | Hspa5 PE | 78 kDa glucose-regulated protein OS | 57.45 | 53 |
| P20152 | Vim PE | Vimentin OS | 99.79 | 53 |
| P11499 | Hsp90ab1 PE | Heat shock protein HSP 90-beta OS | 249.97 | 188 |
| P63017 | Hspa8 PE | Heat shock cognate 71 kDa protein OS | 112.89 | 156 |
| Q9JHU4 | Dync1h1 PE | Cytoplasmic dynein 1 heavy chain 1 OS | 25.2 | 70 |
| P14873 | Map1b PE | Microtubule-associated protein 1B OS | 52.41 | 81 |
| P63038 | Hspd1 PE | 60 kDa heat shock protein, mitochondrial OS | 45.36 | 60 |
| P07901 | Hsp90aa1 PE | Heat shock protein HSP 90-alpha OS | 178.4 | 138 |
| P08113 | Hsp90b1 PE | Endoplasmin OS | 74.59 | 49 |
| Q61879 | Myh10 PE | Myosin-10 OS | 18.14 | 47 |
| P80314 | Cct2 PE | T-complex protein 1 subunit beta OS | 52.41 | 52 |
| P56480 | Atp5b PE | ATP synthase subunit beta, mitochondrial OS | 64.51 | 44 |
| P12894 | 3 SV | Probable Pol polyprotein OS | 70.56 | 58 |
| P11368 | pol PE | Putative Pol polyprotein OS | 38.3 | 26 |
| Q91V92 | Acly PE | ATP-citrate synthase OS | 40.32 | 38 |
| Q68FD5 | Cltc PE | Clathrin heavy chain 1 OS | 31.25 | 26 |
| P58252 | Eef2 PE | Elongation factor 2 OS | 111.88 | 65 |
| P99024 | Tubb5 PE | Tubulin beta-5 chain OS | 190.5 | 218 |
| P52480 | Pkm2 PE | Pyruvate kinase isozymes M1/M2 OS | 37.29 | 56 |
| P17182 | Eno1 PE | Alpha-enolase OS | 123.98 | 123 |
| Q8CGC7 | Eprs PE | Bifunctional aminoacyl-tRNA synthetase OS | 32.25 | 26 |
| Q9JKF1 | Iqgap1 PE | Ras GTPase-activating-like protein IQGAP1 OS | 18.14 | 18 |
| P27773 | Pdia3 PE | Protein disulfide-isomerase A3 OS | 24.19 | 31 |
| Q03265 | Atp5a1 PE | ATP synthase subunit alpha, mitochondrial OS | 44.35 | 44 |
| Q6P5E4 | Ugcgl1 PE | UDP-glucose:glycoprotein glucosyltransferase 1 OS | 16.13 | 16 |
| P38647 | Hspa9 PE | Stress-70 protein, mitochondrial OS | 29.23 | 37 |
| Q9ERD7 | Tubb3 PE | Tubulin beta-3 chain OS | 130.02 | 163 |
| Q99KI0 | Aco2 PE | Aconitate hydratase, mitochondrial OS | 29.23 | 11 |
| Q7TPV4 | Mybbp1a PE | Myb-binding protein 1A OS | 34.27 | 30 |
| P15331 | Prph PE | Peripherin OS | 40.32 | 36 |
| P23116 | Eif3a PE | Eukaryotic translation initiation factor 3 subunit A OS | 19.15 | 17 |
| P14824 | Anxa6 PE | Annexin A6 OS | 12.1 | 15 |
| Q01853 | Vcp PE | Transitional endoplasmic reticulum ATPase OS | 32.25 | 19 |
| Q61316 | Hspa4 PE | Heat shock 70 kDa protein 4 OS | 22.17 | 12 |
| Q02053 | Uba1 PE | Ubiquitin-like modifier-activating enzyme 1 OS | 28.22 | 24 |
| Q6PB66 | Lrpprc PE | Leucine-rich PPR motif-containing protein, mitochondrial OS | 23.18 | 30 |
| Q7TPR4 | Actn1 PE | Alpha-actinin-1 OS | 11.09 | 28 |
| Q78PY7 | Snd1 PE | Staphylococcal nuclease domain-containing protein 1 OS | 20.16 | 14 |
| Q9JKR6 | Hyou1 PE | Hypoxia up-regulated protein 1 OS | 21.17 | 18 |
| P09405 | Ncl PE | Nucleolin OS | 59.47 | 39 |
| Q9Z1Q9 | Vars PE | Valyl-tRNA synthetase OS | 23.18 | 26 |
| P68372 | Tubb2c PE | Tubulin beta-2C chain OS | 138.09 | 158 |
| Q7TMM9 | Tubb2a PE | Tubulin beta-2A chain OS | 119.94 | 140 |
| Q9CWF2 | Tubb2b PE | Tubulin beta-2B chain OS | 118.94 | 140 |
| P29341 | Pabpc1 PE | Polyadenylate-binding protein 1 OS | 18.14 | 21 |
| P11983 | Cct1 PE | T-complex protein 1 subunit alpha B OS | 30.24 | 31 |
| P11984 | Cct1 PE | T-complex protein 1 subunit alpha A OS | 24.19 | 26 |
| Q04447 | Ckb PE | Creatine kinase B-type OS | 0 | 3 |
| P40142 | Tkt PE | Transketolase OS | 15.12 | 21 |
| P68369 | Tuba1a PE | Tubulin alpha-1A chain OS | 197.55 | 177 |
| Q8BMS1 | Hadha PE | Trifunctional enzyme subunit alpha, mitochondrial OS | 11.09 | 3 |
| Q9JIK5 | Ddx21 PE | Nucleolar RNA helicase 2 OS | 31.25 | 13 |
| P70168 | Kpnb1 PE | Importin subunit beta-1 OS | 34.27 | 13 |
| P09411 | Pgk1 PE | Phosphoglycerate kinase 1 OS | 15.12 | 19 |
| Q8BHN3 | Ganab PE | Neutral alpha-glucosidase AB OS | 11.09 | 9 |
| P80317 | Cct6a PE | T-complex protein 1 subunit zeta OS | 29.23 | 30 |
| Q8JZQ9 | Eif3b PE | Eukaryotic translation initiation factor 3 subunit B OS | 20.16 | 13 |
| Q8BGQ7 | Aars PE | Alanyl-tRNA synthetase, cytoplasmic OS | 25.2 | 16 |
| P03975 | Iap PE | IgE-binding protein OS | 42.33 | 68 |
| Q64727 | Vcl PE | Vinculin OS | 8.06 | 14 |
| P60843 | Eif4a1 PE | Eukaryotic initiation factor 4A-I OS | 25.2 | 21 |
| P26041 | Msn PE | Moesin OS | 8.06 | 8 |
| O35685 | Nudc PE | Nuclear migration protein nudC OS | 14.11 | 24 |
| O70133 | Dhx9 PE | ATP-dependent RNA helicase A OS | 23.18 | 21 |
| Q01320 | Top2a PE | DNA topoisomerase 2-alpha OS | 13.1 | 33 |
| P42932 | Cct8 PE | T-complex protein 1 subunit theta OS | 21.17 | 37 |
| Q8BTM8 | Flna PE | Filamin-A OS | 7.06 | 2 |
| Q9D6F9 | Tubb4 PE | Tubulin beta-4 chain OS | 97.77 | 124 |
| Q8BMJ2 | Lars PE | Leucyl-tRNA synthetase, cytoplasmic OS | 18.14 | 20 |
| O08810 | Eftud2 PE | 116 kDa U5 small nuclear ribonucleoprotein component OS | 16.13 | 14 |
| Q9QUR6 | Prep PE | Prolyl endopeptidase OS | 21.17 | 14 |
| Q922D8 | Mthfd1 PE | C-1-tetrahydrofolate synthase, cytoplasmic OS | 15.12 | 9 |
| P60710 | Actb PE | Actin, cytoplasmic 1 OS | 174.37 | 227 |
| P63260 | Actg1 PE | Actin, cytoplasmic 2 OS | 174.37 | 227 |
| P35564 | Canx PE | Calnexin OS | 30.24 | 17 |
| P80315 | Cct4 PE | T-complex protein 1 subunit delta OS | 24.19 | 26 |
| P06151 | Ldha PE | L-lactate dehydrogenase A chain OS | 58.46 | 60 |
| P09103 | P4hb PE | Protein disulfide-isomerase OS | 26.21 | 18 |
| Q9D0E1 | Hnrnpm PE | Heterogeneous nuclear ribonucleoprotein M OS | 14.11 | 16 |
| Q99PV0 | Prpf8 PE | Pre-mRNA-processing-splicing factor 8 OS | 14.11 | 17 |
| P05213 | Tuba1b PE | Tubulin alpha-1B chain OS | 175.38 | 169 |
| P68373 | Tuba1c PE | Tubulin alpha-1C chain OS | 175.38 | 169 |
| P48036 | Anxa5 PE | Annexin A5 OS | 8.06 | 5 |
| P57780 | Actn4 PE | Alpha-actinin-4 OS | 14.11 | 17 |
| O88569 | Hnrnpa2b1 PE | Heterogeneous nuclear ribonucleoproteins A2/B1 OS | 37.29 | 47 |
| P11365 | gag PE | Retrovirus-related Gag polyprotein OS | 38.3 | 60 |
| Q91YQ5 | Rpn1 PE | Dolichyl-diphosphooligosaccharide--protein glycosyltransferase subunit 1 OS | 8.06 | 13 |
| P49312 | Hnrnpa1 PE | Heterogeneous nuclear ribonucleoprotein A1 OS | 57.45 | 50 |
| P16858 | Gapdh PE | Glyceraldehyde-3-phosphate dehydrogenase OS | 47.37 | 74 |
| Q6ZQ38 | Cand1 PE | Cullin-associated NEDD8-dissociated protein 1 OS | 17.13 | 12 |
| Q8K310 | Matr3 PE | Matrin-3 OS | 16.13 | 11 |
| Q8BG05 | Hnrnpa3 PE | Heterogeneous nuclear ribonucleoprotein A3 OS | 26.21 | 22 |
| Q61699 | Hsph1 PE | Heat shock protein 105 kDa OS | 17.13 | 9 |
| Q6P5F9 | Xpo1 PE | Exportin-1 OS | 15.12 | 8 |
| Q3V3R1 | Mthfd1l PE | Monofunctional C1-tetrahydrofolate synthase, mitochondrial OS | 19.15 | 4 |
| P61979 | Hnrnpk PE | Heterogeneous nuclear ribonucleoprotein K OS | 28.22 | 32 |
| P62806 | Hist1h4a PE | Histone H4 OS | 245.93 | 188 |
| O08553 | Dpysl2 PE | Dihydropyrimidinase-related protein 2 OS | 15.12 | 14 |
| P30416 | Fkbp4 PE | FK506-binding protein 4 OS | 11.09 | 11 |
| P08003 | Pdia4 PE | Protein disulfide-isomerase A4 OS | 11.09 | 9 |
| Q99PL5 | Rrbp1 PE | Ribosome-binding protein 1 OS | 13.1 | 12 |
| Q61937 | Npm1 PE | Nucleophosmin OS | 36.29 | 36 |
| Q61598 | Gdi2 PE | Rab GDP dissociation inhibitor beta OS | 26.21 | 22 |
| Q9DBG6 | Rpn2 PE | Dolichyl-diphosphooligosaccharide--protein glycosyltransferase subunit 2 OS | 7.06 | 12 |
| Q9QXS1 | Plec1 PE | Plectin-1 OS | 0 | 1 |
| P97310 | Mcm2 PE | DNA replication licensing factor MCM2 OS | 14.11 | 7 |
| Q62167 | Ddx3x PE | ATP-dependent RNA helicase DDX3X OS | 23.18 | 16 |
| P16381 | D1Pas1 PE | Putative ATP-dependent RNA helicase Pl10 OS | 19.15 | 13 |
| Q9CZD3 | Gars PE | Glycyl-tRNA synthetase OS | 18.14 | 17 |
| Q60864 | Stip1 PE | Stress-induced-phosphoprotein 1 OS | 19.15 | 24 |
| Q8CAQ8 | Immt PE | Mitochondrial inner membrane protein OS | 16.13 | 7 |
| Q8R1B4 | Eif3c PE | Eukaryotic translation initiation factor 3 subunit C OS | 14.11 | 13 |
| Q62351 | Tfrc PE | Transferrin receptor protein 1 OS | 15.12 | 14 |
| Q61656 | Ddx5 PE | Probable ATP-dependent RNA helicase DDX5 OS | 26.21 | 32 |
| Q8VIJ6 | Sfpq PE | Splicing factor, proline- and glutamine-rich OS | 17.13 | 12 |
| P05202 | Got2 PE | Aspartate aminotransferase, mitochondrial OS | 54.43 | 42 |
| P97807 | Fh PE | Fumarate hydratase, mitochondrial OS | 15.12 | 9 |
| P63101 | Ywhaz PE | 14-3-3 protein zeta/delta OS | 34.27 | 51 |
| Q8BKC5 | Ipo5 PE | Importin-5 OS | 19.15 | 13 |
| Q9Z110 | Aldh18a1 PE | Delta-1-pyrroline-5-carboxylate synthetase OS | 16.13 | 4 |
| Q61553 | Fscn1 PE | Fascin OS | 15.12 | 11 |
| Q9WTQ5 | Akap12 PE | A-kinase anchor protein 12 OS | 3.02 | 10 |
| Q922R8 | Pdia6 PE | Protein disulfide-isomerase A6 OS | 18.14 | 13 |
| Q9DCN2 | Cyb5r3 PE | NADH-cytochrome b5 reductase 3 OS | 15.12 | 12 |
| P62908 | Rps3 PE | 40S ribosomal protein S3 OS | 26.21 | 31 |
| P26039 | Tln1 PE | Talin-1 OS | 2.02 | 2 |
| Q9WVA4 | Tagln2 PE | Transgelin-2 OS | 26.21 | 24 |
| P80316 | Cct5 PE | T-complex protein 1 subunit epsilon OS | 27.21 | 20 |
| O35643 | Ap1b1 PE | AP-1 complex subunit beta-1 OS | 11.09 | 7 |
| P80313 | Cct7 PE | T-complex protein 1 subunit eta OS | 31.25 | 29 |
| P14733 | Lmnb1 PE | Lamin-B1 OS | 9.07 | 5 |
| P50580 | Pa2g4 PE | Proliferation-associated protein 2G4 OS | 14.11 | 22 |
| Q9CWJ9 | Atic PE | Bifunctional purine biosynthesis protein PURH OS | 11.09 | 12 |
| Q8VDM4 | Psmd2 PE | 26S proteasome non-ATPase regulatory subunit 2 OS | 14.11 | 6 |
| P08249 | Mdh2 PE | Malate dehydrogenase, mitochondrial OS | 44.35 | 35 |
| Q62318 | Trim28 PE | Transcription intermediary factor 1-beta OS | 22.17 | 9 |
| Q61024 | Asns PE | Asparagine synthetase [glutamine-hydrolyzing] OS | 10.08 | 20 |
| Q99K85 | Psat1 PE | Phosphoserine aminotransferase OS | 20.16 | 22 |
| Q9D0I9 | Rars PE | Arginyl-tRNA synthetase, cytoplasmic OS | 10.08 | 12 |
| P14211 | Calr PE | Calreticulin OS | 8.06 | 12 |
| Q7TMK9 | Syncrip PE | Heterogeneous nuclear ribonucleoprotein Q OS | 10.08 | 9 |
| P10126 | Eef1a1 PE | Elongation factor 1-alpha 1 OS | 281.21 | 169 |
| A2AGT5 | Ckap5 PE | Cytoskeleton-associated protein 5 OS | 7.06 | 14 |
| Q6DFW4 | Nop58 PE | Nucleolar protein 58 OS | 13.1 | 11 |
| Q8BGD9 | Eif4b PE | Eukaryotic translation initiation factor 4B OS | 8.06 | 4 |
| Q9CW03 | Smc3 PE | Structural maintenance of chromosomes protein 3 OS | 5.04 | 9 |
| Q9D8E6 | Rpl4 PE | 60S ribosomal protein L4 OS | 26.21 | 10 |
| P62259 | Ywhae PE | 14-3-3 protein epsilon OS | 18.14 | 23 |
| O35129 | Phb2 PE | Prohibitin-2 OS | 21.17 | 15 |
| Q60932 | Vdac1 PE | Voltage-dependent anion-selective channel protein 1 OS | 29.23 | 23 |
| Q9WTM5 | Ruvbl2 PE | RuvB-like 2 OS | 11.09 | 6 |
| Q8VDN2 | Atp1a1 PE | Sodium/potassium-transporting ATPase subunit alpha-1 OS | 9.07 | 21 |
| Q9DB77 | Uqcrc2 PE | Cytochrome b-c1 complex subunit 2, mitochondrial OS | 13.1 | 10 |
| Q76MZ3 | Ppp2r1a PE | Serine/threonine-protein phosphatase 2A 65 kDa regulatory subunit A alpha isoform OS | 11.09 | 11 |
| Q62188 | Dpysl3 PE | Dihydropyrimidinase-related protein 3 OS | 26.21 | 24 |
| Q64514 | Tpp2 PE | Tripeptidyl-peptidase 2 OS | 8.06 | 12 |
| P47738 | Aldh2 PE | Aldehyde dehydrogenase, mitochondrial OS | 1.01 | 0 |
| P56399 | Usp5 PE | Ubiquitin carboxyl-terminal hydrolase 5 OS | 10.08 | 0 |
| P54775 | Psmc4 PE | 26S protease regulatory subunit 6B OS | 7.06 | 8 |
| Q9CY58 | Serbp1 PE | Plasminogen activator inhibitor 1 RNA-binding protein OS | 11.09 | 7 |
| Q9CQN1 | Trap1 PE | Heat shock protein 75 kDa, mitochondrial OS | 10.08 | 8 |
| P26043 | Rdx PE | Radixin OS | 8.06 | 6 |
| Q9DBG3 | Ap2b1 PE | AP-2 complex subunit beta-1 OS | 10.08 | 8 |
| Q922B2 | Dars PE | Aspartyl-tRNA synthetase, cytoplasmic OS | 11.09 | 9 |
| Q11011 | Npepps PE | Puromycin-sensitive aminopeptidase OS | 3.02 | 3 |
| Q9EQH3 | Vps35 PE | Vacuolar protein sorting-associated protein 35 OS | 9.07 | 1 |
| O35887 | Calu PE | Calumenin OS | 2.02 | 5 |
| Q80X90 | Flnb PE | Filamin-B OS | 4.03 | 6 |
| Q99LC5 | Etfa PE | Electron transfer flavoprotein subunit alpha, mitochondrial OS | 11.09 | 7 |
| P80318 | Cct3 PE | T-complex protein 1 subunit gamma OS | 20.16 | 21 |
| P50516 | Atp6v1a PE | V-type proton ATPase catalytic subunit A OS | 11.09 | 9 |
| P05064 | Aldoa PE | Fructose-bisphosphate aldolase A OS | 32.25 | 28 |
| P46471 | Psmc2 PE | 26S protease regulatory subunit 7 OS | 10.08 | 10 |
| P11440 | Cdc2 PE | Cell division control protein 2 homolog OS | 14.11 | 7 |
| P06745 | Gpi PE | Glucose-6-phosphate isomerase OS | 15.12 | 16 |
| P10649 | Gstm1 PE | Glutathione S-transferase Mu 1 OS | 0 | 1 |
| O08709 | Prdx6 PE | Peroxiredoxin-6 OS | 8.06 | 11 |
| P99027 | Rplp2 PE | 60S acidic ribosomal protein P2 OS | 32.25 | 19 |
| O35737 | Hnrnph1 PE | Heterogeneous nuclear ribonucleoprotein H OS | 30.24 | 15 |
| P67778 | Phb PE | Prohibitin OS | 14.11 | 18 |
| Q91WQ3 | Yars PE | Tyrosyl-tRNA synthetase, cytoplasmic OS | 7.06 | 14 |
| P70372 | Elavl1 PE | ELAV-like protein 1 OS | 23.18 | 20 |
| Q64511 | Top2b PE | DNA topoisomerase 2-beta OS | 4.03 | 7 |
| Q91VD9 | Ndufs1 PE | NADH-ubiquinone oxidoreductase 75 kDa subunit, mitochondrial OS | 2.02 | 0 |
| Q68FL6 | Mars PE | Methionyl-tRNA synthetase, cytoplasmic OS | 12.1 | 6 |
| Q9D6Z1 | Nop56 PE | Nucleolar protein 56 OS | 10.08 | 8 |
| Q91WK2 | Eif3h PE | Eukaryotic translation initiation factor 3 subunit H OS | 7.06 | 6 |
| Q99K48 | Nono PE | Non-POU domain-containing octamer-binding protein OS | 10.08 | 8 |
| Q9DCX2 | Atp5h PE | ATP synthase subunit d, mitochondrial OS | 11.09 | 7 |
| Q99020 | Hnrnpab PE | Heterogeneous nuclear ribonucleoprotein A/B OS | 24.19 | 21 |
| P26443 | Glud1 PE | Glutamate dehydrogenase 1, mitochondrial OS | 4.03 | 0 |
| Q99KJ8 | Dctn2 PE | Dynactin subunit 2 OS | 11.09 | 4 |
| P05201 | Got1 PE | Aspartate aminotransferase, cytoplasmic OS | 13.1 | 15 |
| P17751 | Tpi1 PE | Triosephosphate isomerase OS | 15.12 | 33 |
| Q91ZW3 | Smarca5 PE | SWI/SNF-related matrix-associated actin-dependent regulator of chromatin subfamily A member 5 OS | 8.06 | 7 |
| P48678 | Lmna PE | Lamin-A/C OS | 6.05 | 0 |
| Q9D8N0 | Eef1g PE | Elongation factor 1-gamma OS | 23.18 | 19 |
| Q9Z1N5 | Bat1 PE | Spliceosome RNA helicase Bat1 OS | 16.13 | 8 |
| P43274 | Hist1h1e PE | Histone H1.4 OS | 60.48 | 56 |
| Q9QZM0 | Ubqln2 PE | Ubiquilin-2 OS | 6.05 | 6 |
| Q8VDJ3 | Hdlbp PE | Vigilin OS | 6.05 | 2 |
| P02088 | Hbb-b1 PE | Hemoglobin subunit beta-1 OS | 2.02 | 1 |
| P17225 | Ptbp1 PE | Polypyrimidine tract-binding protein 1 OS | 9.07 | 14 |
| Q9ERK4 | Cse1l PE | Exportin-2 OS | 10.08 | 6 |
| Q6NZJ6 | Eif4g1 PE | Eukaryotic translation initiation factor 4 gamma 1 OS | 8.06 | 11 |
| P52293 | Kpna2 PE | Importin subunit alpha-2 OS | 7.06 | 8 |
| P26638 | Sars PE | Seryl-tRNA synthetase, cytoplasmic OS | 5.04 | 10 |
| P14869 | Rplp0 PE | 60S acidic ribosomal protein P0 OS | 25.2 | 24 |
| P97427 | Crmp1 PE | Dihydropyrimidinase-related protein 1 OS | 10.08 | 10 |
| P97351 | Rps3a PE | 40S ribosomal protein S3a OS | 18.14 | 21 |
| P62702 | Rps4x PE | 40S ribosomal protein S4, X isoform OS | 22.17 | 22 |
| P14148 | Rpl7 PE | 60S ribosomal protein L7 OS | 32.25 | 18 |
| Q3U7R1 | Fam62a PE | Extended synaptotagmin-1 OS | 5.04 | 7 |
| Q99P72 | Rtn4 PE | Reticulon-4 OS | 2.02 | 6 |
| P68040 | Gnb2l1 PE | Guanine nucleotide-binding protein subunit beta-2-like 1 OS | 24.19 | 25 |
| Q9CZ30 | Ola1 PE | Obg-like ATPase 1 OS | 9.07 | 9 |
| P47911 | Rpl6 PE | 60S ribosomal protein L6 OS | 26.21 | 17 |
| Q9DBJ1 | Pgam1 PE | Phosphoglycerate mutase 1 OS | 14.11 | 18 |
| P17710 | Hk1 PE | Hexokinase-1 OS | 10.08 | 5 |
| Q9DCD0 | Pgd PE | 6-phosphogluconate dehydrogenase, decarboxylating OS | 14.11 | 16 |
| P39447 | Tjp1 PE | Tight junction protein ZO-1 OS | 0 | 2 |
| P62270 | Rps18 PE | 40S ribosomal protein S18 OS | 40.32 | 46 |
| Q9DCH4 | Eif3f PE | Eukaryotic translation initiation factor 3 subunit F OS | 11.09 | 8 |
| Q6PIE5 | Atp1a2 PE | Sodium/potassium-transporting ATPase subunit alpha-2 OS | 3.02 | 7 |
| P70296 | Pebp1 PE | Phosphatidylethanolamine-binding protein 1 OS | 16.13 | 16 |
| P28656 | Nap1l1 PE | Nucleosome assembly protein 1-like 1 OS | 25.2 | 22 |
| Q8CG48 | Smc2 PE | Structural maintenance of chromosomes protein 2 OS | 6.05 | 8 |
| Q9EPU0 | Upf1 PE | Regulator of nonsense transcripts 1 OS | 7.06 | 7 |
| Q9CZX8 | Rps19 PE | 40S ribosomal protein S19 OS | 26.21 | 21 |
| Q9CQV8 | Ywhab PE | 14-3-3 protein beta/alpha OS | 16.13 | 19 |
| P62960 | Ybx1 PE | Nuclease-sensitive element-binding protein 1 OS | 7.06 | 8 |
| P10852 | Slc3a2 PE | 4F2 cell-surface antigen heavy chain OS | 11.09 | 10 |
| P27659 | Rpl3 PE | 60S ribosomal protein L3 OS | 14.11 | 8 |
| Q8QZY1 | Eif3l PE | Eukaryotic translation initiation factor 3 subunit L OS | 7.06 | 8 |
| O08788 | Dctn1 PE | Dynactin subunit 1 OS | 6.05 | 3 |
| Q9DBE9 | Ftsj3 PE | Putative rRNA methyltransferase 3 OS | 5.04 | 4 |
| P43275 | Hist1h1a PE | Histone H1.1 OS | 42.33 | 26 |
| Q9CZU6 | Cs PE | Citrate synthase, mitochondrial OS | 9.07 | 9 |
| Q9EPL8 | Ipo7 PE | Importin-7 OS | 11.09 | 5 |
| Q8BP47 | Nars PE | Asparaginyl-tRNA synthetase, cytoplasmic OS | 6.05 | 9 |
| P24369 | Ppib PE | Peptidyl-prolyl cis-trans isomerase B OS | 12.1 | 13 |
| Q99JY0 | Hadhb PE | Trifunctional enzyme subunit beta, mitochondrial OS | 3.02 | 1 |
| Q91YI0 | Asl PE | Argininosuccinate lyase OS | 4.03 | 10 |
| P61222 | Abce1 PE | ATP-binding cassette sub-family E member 1 OS | 9.07 | 13 |
| P25444 | Rps2 PE | 40S ribosomal protein S2 OS | 23.18 | 12 |
| P17426 | Ap2a1 PE | AP-2 complex subunit alpha-1 OS | 7.06 | 2 |
| P97311 | Mcm6 PE | DNA replication licensing factor MCM6 OS | 9.07 | 3 |
| Q8K1M6 | Dnm1l PE | Dynamin-1-like protein OS | 8.06 | 3 |
| P43276 | Hist1h1b PE | Histone H1.5 OS | 40.32 | 29 |
| Q99PT1 | Arhgdia PE | Rho GDP-dissociation inhibitor 1 OS | 7.06 | 12 |
| Q921M3 | Sf3b3 PE | Splicing factor 3B subunit 3 OS | 7.06 | 15 |
| O88685 | Psmc3 PE | 26S protease regulatory subunit 6A OS | 5.04 | 4 |
| P14685 | Psmd3 PE | 26S proteasome non-ATPase regulatory subunit 3 OS | 8.06 | 6 |
| Q91WJ8 | Fubp1 PE | Far upstream element-binding protein 1 OS | 7.06 | 6 |
| Q9JIF0 | Prmt1 PE | Protein arginine N-methyltransferase 1 OS | 12.1 | 6 |
| Q61768 | Kif5b PE | Kinesin-1 heavy chain OS | 7.06 | 5 |
| P50396 | Gdi1 PE | Rab GDP dissociation inhibitor alpha OS | 12.1 | 21 |
| Q8R081 | Hnrnpl PE | Heterogeneous nuclear ribonucleoprotein L OS | 6.05 | 5 |
| P50544 | Acadvl PE | Very long-chain specific acyl-CoA dehydrogenase, mitochondrial OS | 1.01 | 1 |
| P68510 | Ywhah PE | 14-3-3 protein eta OS | 13.1 | 20 |
| Q8CIE6 | Copa PE | Coatomer subunit alpha OS | 7.06 | 13 |
| Q99MN1 | Kars PE | Lysyl-tRNA synthetase OS | 7.06 | 6 |
| P35700 | Prdx1 PE | Peroxiredoxin-1 OS | 17.13 | 25 |
| P62631 | Eef1a2 PE | Elongation factor 1-alpha 2 OS | 254 | 141 |
| Q9Z204 | Hnrnpc PE | Heterogeneous nuclear ribonucleoproteins C1/C2 OS | 14.11 | 11 |
| P02089 | Hbb-b2 PE | Hemoglobin subunit beta-2 OS | 2.02 | 1 |
| Q8VDP4 | 1 SV | Protein KIAA1967 homolog OS | 2.02 | 5 |
| Q61171 | Prdx2 PE | Peroxiredoxin-2 OS | 24.19 | 21 |
| P12970 | Rpl7a PE | 60S ribosomal protein L7a OS | 19.15 | 27 |
| O55234 | Psmb5 PE | Proteasome subunit beta type-5 OS | 17.13 | 11 |
| P51150 | Rab7a PE | Ras-related protein Rab-7a OS | 13.1 | 13 |
| Q8BG32 | Psmd11 PE | 26S proteasome non-ATPase regulatory subunit 11 OS | 4.03 | 5 |
| P32067 | Ssb PE | Lupus La protein homolog OS | 11.09 | 6 |
| P61982 | Ywhag PE | 14-3-3 protein gamma OS | 13.1 | 20 |
| P17427 | Ap2a2 PE | AP-2 complex subunit alpha-2 OS | 9.07 | 3 |
| P14206 | Rpsa PE | 40S ribosomal protein SA OS | 21.17 | 15 |
| Q6ZWN5 | Rps9 PE | 40S ribosomal protein S9 OS | 24.19 | 25 |
| O08795 | Prkcsh PE | Glucosidase 2 subunit beta OS | 7.06 | 1 |
| P37913 | Lig1 PE | DNA ligase 1 OS | 6.05 | 6 |
| Q91YE6 | Ipo9 PE | Importin-9 OS | 4.03 | 6 |
| P49722 | Psma2 PE | Proteasome subunit alpha type-2 OS | 1.01 | 5 |
| P68254 | Ywhaq PE | 14-3-3 protein theta OS | 11.09 | 16 |
| Q9CZ13 | Uqcrc1 PE | Cytochrome b-c1 complex subunit 1, mitochondrial OS | 6.05 | 2 |
| P57722 | Pcbp3 PE | Poly(rC)-binding protein 3 OS | 13.1 | 7 |
| Q91VC3 | Eif4a3 PE | Eukaryotic initiation factor 4A-III OS | 9.07 | 5 |
| Q3TXS7 | Psmd1 PE | 26S proteasome non-ATPase regulatory subunit 1 OS | 4.03 | 3 |
| Q8CI94 | Pygb PE | Glycogen phosphorylase, brain form OS | 4.03 | 0 |
| P18760 | Cfl1 PE | Cofilin-1 OS | 30.24 | 32 |
| Q9R0P9 | Uchl1 PE | Ubiquitin carboxyl-terminal hydrolase isozyme L1 OS | 8.06 | 16 |
| Q8VDW0 | Ddx39 PE | ATP-dependent RNA helicase DDX39 OS | 15.12 | 7 |
| P17742 | Ppia PE | Peptidyl-prolyl cis-trans isomerase A OS | 37.29 | 39 |
| Q8K224 | Nat10 PE | N-acetyltransferase 10 OS | 7.06 | 1 |
| Q8BJ71 | Nup93 PE | Nuclear pore complex protein Nup93 OS | 8.06 | 1 |
| O88544 | Cops4 PE | COP9 signalosome complex subunit 4 OS | 8.06 | 10 |
| P97496 | Smarcc1 PE | SWI/SNF complex subunit SMARCC1 OS | 4.03 | 7 |
| Q8C1A5 | Thop1 PE | Thimet oligopeptidase OS | 7.06 | 3 |
| P49718 | Mcm5 PE | DNA replication licensing factor MCM5 OS | 7.06 | 4 |
| P54227 | Stmn1 PE | Stathmin OS | 13.1 | 17 |
| P70333 | Hnrnph2 PE | Heterogeneous nuclear ribonucleoprotein H2 OS | 17.13 | 10 |
| P42208 | Sept2 PE | Septin-2 OS | 4.03 | 3 |
| Q6PDQ2 | Chd4 PE | Chromodomain-helicase-DNA-binding protein 4 OS | 5.04 | 7 |
| Q99MR6 | Srrt PE | Serrate RNA effector molecule homolog OS | 4.03 | 8 |
| P46061 | Rangap1 PE | Ran GTPase-activating protein 1 OS | 7.06 | 8 |
| Q8CGK3 | Lonp1 PE | Lon protease homolog, mitochondrial OS | 6.05 | 3 |
| Q6ZWX6 | Eif2s1 PE | Eukaryotic translation initiation factor 2 subunit 1 OS | 8.06 | 5 |
| P60229 | Eif3e PE | Eukaryotic translation initiation factor 3 subunit E OS | 10.08 | 7 |
| P19157 | Gstp1 PE | Glutathione S-transferase P 1 OS | 8.06 | 15 |
| P18155 | Mthfd2 PE | Bifunctional methylenetetrahydrofolate dehydrogenase/cyclohydrolase, mitochondrial OS | 5.04 | 4 |
| Q6PDM2 | Sfrs1 PE | Splicing factor, arginine/serine-rich 1 OS | 12.1 | 10 |
| P62196 | Psmc5 PE | 26S protease regulatory subunit 8 OS | 8.06 | 7 |
| Q9CZ44 | Nsfl1c PE | NSFL1 cofactor p47 OS | 5.04 | 7 |
| Q3THK7 | Gmps PE | GMP synthase [glutamine-hydrolyzing] OS | 7.06 | 5 |
| Q6PDG5 | Smarcc2 PE | SWI/SNF complex subunit SMARCC2 OS | 5.04 | 7 |
| Q60931 | Vdac3 PE | Voltage-dependent anion-selective channel protein 3 OS | 15.12 | 12 |
| Q05D44 | Eif5b PE | Eukaryotic translation initiation factor 5B OS | 5.04 | 7 |
| Q61753 | Phgdh PE | D-3-phosphoglycerate dehydrogenase OS | 11.09 | 11 |
| O35286 | Dhx15 PE | Putative pre-mRNA-splicing factor ATP-dependent RNA helicase DHX15 OS | 9.07 | 4 |
| P26040 | Ezr PE | Ezrin OS | 7.06 | 7 |
| Q9CXW3 | Cacybp PE | Calcyclin-binding protein OS | 12.1 | 10 |
| Q62446 | Fkbp3 PE | FK506-binding protein 3 OS | 7.06 | 8 |
| Q62465 | Vat1 PE | Synaptic vesicle membrane protein VAT-1 homolog OS | 5.04 | 4 |
| Q8CG47 | Smc4 PE | Structural maintenance of chromosomes protein 4 OS | 3.02 | 7 |
| P29391 | Ftl1 PE | Ferritin light chain 1 OS | 14.11 | 1 |
| Q61033 | Tmpo PE | Lamina-associated polypeptide 2, isoforms alpha/zeta OS | 8.06 | 3 |
| Q920B9 | Supt16h PE | FACT complex subunit SPT16 OS | 2.02 | 8 |
| P07742 | Rrm1 PE | Ribonucleoside-diphosphate reductase large subunit OS | 6.05 | 1 |
| P28738 | Kif5c PE | Kinesin heavy chain isoform 5C OS | 7.06 | 7 |
| Q7TQI3 | Otub1 PE | Ubiquitin thioesterase OTUB1 OS | 8.06 | 3 |
| Q60865 | Caprin1 PE | Caprin-1 OS | 8.06 | 4 |
| Q8BMK4 | Ckap4 PE | Cytoskeleton-associated protein 4 OS | 6.05 | 1 |
| O70194 | Eif3d PE | Eukaryotic translation initiation factor 3 subunit D OS | 8.06 | 6 |
| Q9D6R2 | Idh3a PE | Isocitrate dehydrogenase [NAD] subunit alpha, mitochondrial OS | 10.08 | 9 |
| P35278 | Rab5c PE | Ras-related protein Rab-5C OS | 4.03 | 8 |
| P62242 | Rps8 PE | 40S ribosomal protein S8 OS | 31.25 | 32 |
| Q9QZE5 | Copg PE | Coatomer subunit gamma OS | 5.04 | 3 |
| Q8C1B7 | Sept11 PE | Septin-11 OS | 8.06 | 2 |
| P14152 | Mdh1 PE | Malate dehydrogenase, cytoplasmic OS | 11.09 | 20 |
| Q64674 | Srm PE | Spermidine synthase OS | 10.08 | 9 |
| Q9JHR7 | Ide PE | Insulin-degrading enzyme OS | 3.02 | 4 |
| Q9DCW4 | Etfb PE | Electron transfer flavoprotein subunit beta OS | 5.04 | 9 |
| Q6IRU2 | Tpm4 PE | Tropomyosin alpha-4 chain OS | 8.06 | 7 |
| Q9Z2U0 | Psma7 PE | Proteasome subunit alpha type-7 OS | 7.06 | 9 |
| Q9D0R2 | Tars PE | Threonyl-tRNA synthetase, cytoplasmic OS | 5.04 | 4 |
| P97822 | Anp32e PE | Acidic leucine-rich nuclear phosphoprotein 32 family member E OS | 7.06 | 8 |
| P35980 | Rpl18 PE | 60S ribosomal protein L18 OS | 34.27 | 26 |
| O55131 | Sept7 PE | Septin-7 OS | 3.02 | 1 |
| P26645 | Marcks PE | Myristoylated alanine-rich C-kinase substrate OS | 3.02 | 3 |
| P30681 | Hmgb2 PE | High mobility group protein B2 OS | 9.07 | 13 |
| P62751 | Rpl23a PE | 60S ribosomal protein L23a OS | 13.1 | 12 |
| P97855 | G3bp1 PE | Ras GTPase-activating protein-binding protein 1 OS | 6.05 | 5 |
| P63085 | Mapk1 PE | Mitogen-activated protein kinase 1 OS | 2.02 | 5 |
| P60122 | Ruvbl1 PE | RuvB-like 1 OS | 10.08 | 4 |
| P46660 | Ina PE | Alpha-internexin OS | 4.03 | 3 |
| Q60597 | Ogdh PE | 2-oxoglutarate dehydrogenase E1 component, mitochondrial OS | 7.06 | 5 |
| P46638 | Rab11b PE | Ras-related protein Rab-11B OS | 5.04 | 9 |
| Q9Z2U1 | Psma5 PE | Proteasome subunit alpha type-5 OS | 7.06 | 9 |
| Q3UPL0 | Sec31a PE | Protein transport protein Sec31A OS | 3.02 | 3 |
| Q9D880 | Timm50 PE | Mitochondrial import inner membrane translocase subunit TIM50 OS | 6.05 | 6 |
| P51410 | Rpl9 PE | 60S ribosomal protein L9 OS | 14.11 | 13 |
| P51174 | Acadl PE | Long-chain specific acyl-CoA dehydrogenase, mitochondrial OS | 1.01 | 1 |
| Q9CQ65 | Mtap PE | S-methyl-5'-thioadenosine phosphorylase OS | 3.02 | 6 |
| Q60737 | Csnk2a1 PE | Casein kinase II subunit alpha OS | 4.03 | 3 |
| Q99NB9 | Sf3b1 PE | Splicing factor 3B subunit 1 OS | 6.05 | 6 |
| Q9WTP6 | Ak2 PE | Adenylate kinase 2, mitochondrial OS | 6.05 | 4 |
| P16014 | Chgb PE | Secretogranin-1 OS | 1.01 | 7 |
| Q9DB20 | Atp5o PE | ATP synthase subunit O, mitochondrial OS | 12.1 | 13 |
| P62204 | Calm1 PE | Calmodulin OS | 8.06 | 9 |
| Q9Z2X1 | Hnrnpf PE | Heterogeneous nuclear ribonucleoprotein F OS | 24.19 | 13 |
| Q93092 | Taldo1 PE | Transaldolase OS | 8.06 | 9 |
| P23198 | Cbx3 PE | Chromobox protein homolog 3 OS | 11.09 | 12 |
| Q99LF4 | D10Wsu52e PE | UPF0027 protein C22orf28 homolog OS | 0 | 7 |
| P99026 | Psmb4 PE | Proteasome subunit beta type-4 OS | 3.02 | 6 |
| O55143 | Atp2a2 PE | Sarcoplasmic/endoplasmic reticulum calcium ATPase 2 OS | 1.01 | 15 |
| P63158 | Hmgb1 PE | High mobility group protein B1 OS | 10.08 | 7 |
| Q8BH59 | Slc25a12 PE | Calcium-binding mitochondrial carrier protein Aralar1 OS | 5.04 | 5 |
| Q91VR5 | Ddx1 PE | ATP-dependent RNA helicase DDX1 OS | 7.06 | 1 |
| P62137 | Ppp1ca PE | Serine/threonine-protein phosphatase PP1-alpha catalytic subunit OS | 8.06 | 6 |
| P62301 | Rps13 PE | 40S ribosomal protein S13 OS | 16.13 | 14 |
| Q9DC69 | Ndufa9 PE | NADH dehydrogenase [ubiquinone] 1 alpha subcomplex subunit 9, mitochondrial OS | 2.02 | 1 |
| Q99JI6 | Rap1b PE | Ras-related protein Rap-1b OS | 5.04 | 3 |
| P26350 | Ptma PE | Prothymosin alpha OS | 19.15 | 16 |
| P26516 | Psmd7 PE | 26S proteasome non-ATPase regulatory subunit 7 OS | 5.04 | 6 |
| Q9Z2I8 | Suclg2 PE | Succinyl-CoA ligase [GDP-forming] subunit beta, mitochondrial OS | 2.02 | 6 |
| P56959 | Fus PE | RNA-binding protein FUS OS | 7.06 | 2 |
| P62082 | Rps7 PE | 40S ribosomal protein S7 OS | 11.09 | 9 |
| Q3U0V1 | Khsrp PE | Far upstream element-binding protein 2 OS | 7.06 | 2 |
| Q91XV3 | Basp1 PE | Brain acid soluble protein 1 OS | 11.09 | 6 |
| Q99JY9 | Actr3 PE | Actin-related protein 3 OS | 4.03 | 2 |
| O54734 | Ddost PE | Dolichyl-diphosphooligosaccharide--protein glycosyltransferase 48 kDa subunit OS | 6.05 | 5 |
| Q91V12 | Acot7 PE | Cytosolic acyl coenzyme A thioester hydrolase OS | 12.1 | 14 |
| Q8BYA0 | Tbcd PE | Tubulin-specific chaperone D OS | 5.04 | 2 |
| Q8BSL7 | Arf2 PE | ADP-ribosylation factor 2 OS | 5.04 | 6 |
| P67984 | Rpl22 PE | 60S ribosomal protein L22 OS | 16.13 | 14 |
| Q920E5 | Fdps PE | Farnesyl pyrophosphate synthetase OS | 9.07 | 8 |
| Q9CQE8 | 2 SV | UPF0568 protein C14orf166 homolog OS | 3.02 | 4 |
| Q9WUA3 | Pfkp PE | 6-phosphofructokinase type C OS | 7.06 | 1 |
| Q3TEA8 | Hp1bp3 PE | Heterochromatin protein 1-binding protein 3 OS | 3.02 | 1 |
| P57776 | Eef1d PE | Elongation factor 1-delta OS | 12.1 | 10 |
| Q91VR2 | Atp5c1 PE | ATP synthase subunit gamma, mitochondrial OS | 13.1 | 13 |
| Q60668 | Hnrnpd PE | Heterogeneous nuclear ribonucleoprotein D0 OS | 8.06 | 8 |
| P62192 | Psmc1 PE | 26S protease regulatory subunit 4 OS | 6.05 | 5 |
| Q921F2 | Tardbp PE | TAR DNA-binding protein 43 OS | 16.13 | 11 |
| P62754 | Rps6 PE | 40S ribosomal protein S6 OS | 13.1 | 7 |
| P70670 | Naca PE | Nascent polypeptide-associated complex subunit alpha, muscle-specific form OS | 17.13 | 13 |
| P48962 | Slc25a4 PE | ADP/ATP translocase 1 OS | 36.29 | 49 |
| P62962 | Pfn1 PE | Profilin-1 OS | 14.11 | 14 |
| Q9R0Y5 | Ak1 PE | Adenylate kinase isoenzyme 1 OS | 4.03 | 4 |
| Q922U2 | Krt5 PE | Keratin, type II cytoskeletal 5 OS | 20.16 | 36 |
| P15532 | Nme1 PE | Nucleoside diphosphate kinase A OS | 14.11 | 14 |
| Q9DCT2 | Ndufs3 PE | NADH dehydrogenase [ubiquinone] iron-sulfur protein 3, mitochondrial OS | 6.05 | 0 |
| Q9D0I8 | Mrto4 PE | mRNA turnover protein 4 homolog OS | 11.09 | 9 |
| Q8K2B3 | Sdha PE | Succinate dehydrogenase [ubiquinone] flavoprotein subunit, mitochondrial OS | 2.02 | 5 |
| P53026 | Rpl10a PE | 60S ribosomal protein L10a OS | 14.11 | 11 |
| O70251 | Eef1b PE | Elongation factor 1-beta OS | 22.17 | 26 |
| Q9Z1R2 | Bat3 PE | Large proline-rich protein BAT3 OS | 4.03 | 7 |
| Q9D1J3 | Hcc1 PE | Nuclear protein Hcc-1 OS | 7.06 | 7 |
| P20357 | Map2 PE | Microtubule-associated protein 2 OS | 0 | 1 |
| Q99JI4 | Psmd6 PE | 26S proteasome non-ATPase regulatory subunit 6 OS | 6.05 | 3 |
| Q9D819 | Ppa1 PE | Inorganic pyrophosphatase OS | 5.04 | 7 |
| P61255 | Rpl26 PE | 60S ribosomal protein L26 OS | 11.09 | 8 |
| Q8JZK9 | Hmgcs1 PE | Hydroxymethylglutaryl-CoA synthase, cytoplasmic OS | 0 | 3 |
| Q8VI75 | Ipo4 PE | Importin-4 OS | 2.02 | 3 |
| Q80TB8 | Vat1l PE | Synaptic vesicle membrane protein VAT-1 homolog-like OS | 7.06 | 4 |
| O70435 | Psma3 PE | Proteasome subunit alpha type-3 OS | 6.05 | 8 |
| P49717 | Mcm4 PE | DNA replication licensing factor MCM4 OS | 6.05 | 1 |
| Q8QZT1 | Acat1 PE | Acetyl-CoA acetyltransferase, mitochondrial OS | 6.05 | 6 |
| Q9EQU5 | Set PE | Protein SET OS | 10.08 | 10 |
| Q61881 | Mcm7 PE | DNA replication licensing factor MCM7 OS | 8.06 | 4 |
| O08807 | Prdx4 PE | Peroxiredoxin-4 OS | 8.06 | 8 |
| Q9CXW4 | Rpl11 PE | 60S ribosomal protein L11 OS | 13.1 | 12 |
| Q3UEB3 | Puf60 PE | Poly(U)-binding-splicing factor PUF60 OS | 5.04 | 3 |
| P47962 | Rpl5 PE | 60S ribosomal protein L5 OS | 10.08 | 7 |
| Q60605 | Myl6 PE | Myosin light polypeptide 6 OS | 7.06 | 15 |
| Q8BH04 | Pck2 PE | Phosphoenolpyruvate carboxykinase [GTP], mitochondrial OS | 3.02 | 6 |
| Q99KK7 | Dpp3 PE | Dipeptidyl-peptidase 3 OS | 5.04 | 1 |
| Q14C51 | Ptcd3 PE | Pentatricopeptide repeat-containing protein 3, mitochondrial OS | 6.05 | 0 |
| P70699 | Gaa PE | Lysosomal alpha-glucosidase OS | 3.02 | 4 |
| Q60930 | Vdac2 PE | Voltage-dependent anion-selective channel protein 2 OS | 7.06 | 8 |
| P06837 | Gap43 PE | Neuromodulin OS | 4.03 | 4 |
| Q8BHC4 | Dcakd PE | Dephospho-CoA kinase domain-containing protein OS | 9.07 | 8 |
| P61290 | Psme3 PE | Proteasome activator complex subunit 3 OS | 6.05 | 7 |
| Q8BFY9 | Tnpo1 PE | Transportin-1 OS | 7.06 | 5 |
| Q8R1M2 | H2afj PE | Histone H2A.J OS | 195.54 | 121 |
| Q9WV55 | Vapa PE | Vesicle-associated membrane protein-associated protein A OS | 7.06 | 8 |
| P18242 | Ctsd PE | Cathepsin D OS | 0 | 1 |
| Q9WUP7 | Uchl5 PE | Ubiquitin carboxyl-terminal hydrolase isozyme L5 OS | 2.02 | 6 |
| P63242 | Eif5a PE | Eukaryotic translation initiation factor 5A-1 OS | 17.13 | 13 |
| Q80X50 | Ubap2l PE | Ubiquitin-associated protein 2-like OS | 4.03 | 4 |
| Q8BMF4 | Dlat PE | Dihydrolipoyllysine-residue acetyltransferase component of pyruvate dehydrogenase complex, mitochondrial OS | 3.02 | 7 |
| P13595 | Ncam1 PE | Neural cell adhesion molecule 1 OS | 7.06 | 2 |
| P07724 | Alb PE | Serum albumin OS | 5.04 | 39 |
| Q5SWD9 | Tsr1 PE | Pre-rRNA-processing protein TSR1 homolog OS | 4.03 | 4 |
| P62141 | Ppp1cb PE | Serine/threonine-protein phosphatase PP1-beta catalytic subunit OS | 8.06 | 6 |
| Q78ZA7 | Nap1l4 PE | Nucleosome assembly protein 1-like 4 OS | 8.06 | 8 |
| Q3THE2 | Myl12b PE | Myosin regulatory light chain 12B OS | 11.09 | 8 |
| Q5XG71 | Utp20 PE | Small subunit processome component 20 homolog OS | 4.03 | 1 |
| P35550 | Fbl PE | rRNA 2'-O-methyltransferase fibrillarin OS | 7.06 | 3 |
| P62821 | Rab1A PE | Ras-related protein Rab-1A OS | 16.13 | 10 |
| Q9QZQ8 | H2afy PE | Core histone macro-H2A.1 OS | 6.05 | 1 |
| Q9CQQ7 | Atp5f1 PE | ATP synthase subunit b, mitochondrial OS | 3.02 | 3 |
| Q8K4Z5 | Sf3a1 PE | Splicing factor 3 subunit 1 OS | 7.06 | 1 |
| Q99L47 | St13 PE | Hsc70-interacting protein OS | 8.06 | 7 |
| O89086 | Rbm3 PE | Putative RNA-binding protein 3 OS | 7.06 | 5 |
| P06801 | Me1 PE | NADP-dependent malic enzyme OS | 1.01 | 3 |
| P29758 | Oat PE | Ornithine aminotransferase, mitochondrial OS | 6.05 | 1 |
| P62827 | Ran PE | GTP-binding nuclear protein Ran OS | 15.12 | 19 |
| Q9CZM2 | Rpl15 PE | 60S ribosomal protein L15 OS | 5.04 | 8 |
| P25206 | Mcm3 PE | DNA replication licensing factor MCM3 OS | 5.04 | 2 |
| Q922K7 | Nop2 PE | Putative ribosomal RNA methyltransferase NOP2 OS | 4.03 | 2 |
| P27546 | Map4 PE | Microtubule-associated protein 4 OS | 2.02 | 0 |
| P60335 | Pcbp1 PE | Poly(rC)-binding protein 1 OS | 10.08 | 6 |
| P13864 | Dnmt1 PE | DNA (cytosine-5)-methyltransferase 1 OS | 4.03 | 3 |
| Q8R326 | Pspc1 PE | Paraspeckle component 1 OS | 4.03 | 1 |
| P53994 | Rab2a PE | Ras-related protein Rab-2A OS | 6.05 | 7 |
| P51881 | Slc25a5 PE | ADP/ATP translocase 2 OS | 36.29 | 46 |
| P18872 | Gnao1 PE | Guanine nucleotide-binding protein G(o) subunit alpha OS | 3.02 | 4 |
| P62814 | Atp6v1b2 PE | V-type proton ATPase subunit B, brain isoform OS | 5.04 | 4 |
| Q922Q8 | Lrrc59 PE | Leucine-rich repeat-containing protein 59 OS | 9.07 | 6 |
| Q9CPU0 | Glo1 PE | Lactoylglutathione lyase OS | 4.03 | 4 |
| P51660 | Hsd17b4 PE | Peroxisomal multifunctional enzyme type 2 OS | 3.02 | 2 |
| Q9WVJ2 | Psmd13 PE | 26S proteasome non-ATPase regulatory subunit 13 OS | 3.02 | 4 |
| Q9QZD9 | Eif3i PE | Eukaryotic translation initiation factor 3 subunit I OS | 5.04 | 4 |
| Q9CQD1 | Rab5a PE | Ras-related protein Rab-5A OS | 2.02 | 5 |
| Q9QYB1 | Clic4 PE | Chloride intracellular channel protein 4 OS | 2.02 | 7 |
| Q9D8W5 | Psmd12 PE | 26S proteasome non-ATPase regulatory subunit 12 OS | 7.06 | 2 |
| P34022 | Ranbp1 PE | Ran-specific GTPase-activating protein OS | 7.06 | 11 |
| P54116 | Stom PE | Erythrocyte band 7 integral membrane protein OS | 0 | 6 |
| P62991 | Rps27a PE | Ubiquitin OS | 36.29 | 58 |
| Q8BU30 | Iars PE | Isoleucyl-tRNA synthetase, cytoplasmic OS | 3.02 | 4 |
| Q9JKB3 | Csda PE | DNA-binding protein A OS | 5.04 | 5 |
| Q9R1P1 | Psmb3 PE | Proteasome subunit beta type-3 OS | 6.05 | 6 |
| Q8K363 | Ddx18 PE | ATP-dependent RNA helicase DDX18 OS | 5.04 | 0 |
| Q9Z1Z0 | Uso1 PE | General vesicular transport factor p115 OS | 2.02 | 3 |
| P02535 | Krt10 PE | Keratin, type I cytoskeletal 10 OS | 11.09 | 35 |
| Q8K0D5 | Gfm1 PE | Elongation factor G 1, mitochondrial OS | 6.05 | 1 |
| P17918 | Pcna PE | Proliferating cell nuclear antigen OS | 7.06 | 4 |
| P97371 | Psme1 PE | Proteasome activator complex subunit 1 OS | 6.05 | 7 |
| Q8BK64 | Ahsa1 PE | Activator of 90 kDa heat shock protein ATPase homolog 1 OS | 3.02 | 3 |
| Q05920 | Pc PE | Pyruvate carboxylase, mitochondrial OS | 0 | 5 |
| Q61029 | Tmpo PE | Lamina-associated polypeptide 2, isoforms beta/delta/epsilon/gamma OS | 7.06 | 5 |
| Q9R0N0 | Galk1 PE | Galactokinase OS | 3.02 | 2 |
| O35381 | Anp32a PE | Acidic leucine-rich nuclear phosphoprotein 32 family member A OS | 3.02 | 9 |
| Q9WVA3 | Bub3 PE | Mitotic checkpoint protein BUB3 OS | 4.03 | 1 |
| Q5SUR0 | Pfas PE | Phosphoribosylformylglycinamidine synthase OS | 4.03 | 3 |
| Q99JX4 | Eif3m PE | Eukaryotic translation initiation factor 3 subunit M OS | 3.02 | 5 |
| Q8BK67 | Rcc2 PE | Protein RCC2 OS | 6.05 | 3 |
| P61161 | Actr2 PE | Actin-related protein 2 OS | 5.04 | 6 |
| Q9CPY7 | Lap3 PE | Cytosol aminopeptidase OS | 3.02 | 4 |
| Q8BI84 | Mia3 PE | Melanoma inhibitory activity protein 3 OS | 0 | 6 |
| P12382 | Pfkl PE | 6-phosphofructokinase, liver type OS | 4.03 | 2 |
| Q9D0K2 | Oxct1 PE | Succinyl-CoA:3-ketoacid-coenzyme A transferase 1, mitochondrial OS | 4.03 | 8 |
| P99029 | Prdx5 PE | Peroxiredoxin-5, mitochondrial OS | 0 | 1 |
| P50247 | Ahcy PE | Adenosylhomocysteinase OS | 5.04 | 8 |
| P26369 | U2af2 PE | Splicing factor U2AF 65 kDa subunit OS | 4.03 | 3 |
| Q64012 | Raly PE | RNA-binding protein Raly OS | 5.04 | 4 |
| P47963 | Rpl13 PE | 60S ribosomal protein L13 OS | 13.1 | 14 |
| Q8VDF2 | Uhrf1 PE | E3 ubiquitin-protein ligase UHRF1 OS | 5.04 | 0 |
| Q01405 | Sec23a PE | Protein transport protein Sec23A OS | 5.04 | 1 |
| Q9CU62 | Smc1a PE | Structural maintenance of chromosomes protein 1A OS | 1.01 | 4 |
| Q9R1P4 | Psma1 PE | Proteasome subunit alpha type-1 OS | 5.04 | 4 |
| Q08943 | Ssrp1 PE | FACT complex subunit SSRP1 OS | 2.02 | 1 |
| P57716 | Ncstn PE | Nicastrin OS | 4.03 | 3 |
| Q9CRB9 | Chchd3 PE | Coiled-coil-helix-coiled-coil-helix domain-containing protein 3, mitochondrial OS | 4.03 | 3 |
| P54071 | Idh2 PE | Isocitrate dehydrogenase [NADP], mitochondrial OS | 2.02 | 2 |
| P63325 | Rps10 PE | 40S ribosomal protein S10 OS | 13.1 | 11 |
| Q8R317 | Ubqln1 PE | Ubiquilin-1 OS | 2.02 | 1 |
| Q9CR16 | Ppid PE | 40 kDa peptidyl-prolyl cis-trans isomerase OS | 2.02 | 3 |
| Q99ME9 | Gtpbp4 PE | Nucleolar GTP-binding protein 1 OS | 4.03 | 3 |
| P21107 | Tpm3 PE | Tropomyosin alpha-3 chain OS | 5.04 | 5 |
| P14131 | Rps16 PE | 40S ribosomal protein S16 OS | 15.12 | 14 |
| O35691 | Pnn PE | Pinin OS | 3.02 | 1 |
| Q6URW6 | Myh14 PE | Myosin-14 OS | 5.04 | 8 |
| Q9CXY6 | Ilf2 PE | Interleukin enhancer-binding factor 2 OS | 3.02 | 3 |
| Q9D0F9 | Pgm1 PE | Phosphoglucomutase-1 OS | 0 | 1 |
| P41105 | Rpl28 PE | 60S ribosomal protein L28 OS | 5.04 | 7 |
| Q1HFZ0 | Nsun2 PE | tRNA (cytosine-5-)-methyltransferase NSUN2 OS | 6.05 | 2 |
| Q99L45 | Eif2s2 PE | Eukaryotic translation initiation factor 2 subunit 2 OS | 7.06 | 0 |
| O35658 | C1qbp PE | Complement component 1 Q subcomponent-binding protein, mitochondrial OS | 6.05 | 5 |
| P61924 | Copz1 PE | Coatomer subunit zeta-1 OS | 5.04 | 4 |
| O54984 | Asna1 PE | Arsenical pump-driving ATPase OS | 3.02 | 4 |
| Q9CQA3 | Sdhb PE | Succinate dehydrogenase [ubiquinone] iron-sulfur subunit, mitochondrial OS | 1.01 | 2 |
| P62334 | Psmc6 PE | 26S protease regulatory subunit S10B OS | 5.04 | 4 |
| Q6ZQ08 | Cnot1 PE | CCR4-NOT transcription complex subunit 1 OS | 1.01 | 5 |
| Q9CQ60 | Pgls PE | 6-phosphogluconolactonase OS | 1.01 | 5 |
| Q60899 | Elavl2 PE | ELAV-like protein 2 OS | 3.02 | 5 |
| Q8VEM8 | Slc25a3 PE | Phosphate carrier protein, mitochondrial OS | 12.1 | 16 |
| P61164 | Actr1a PE | Alpha-centractin OS | 4.03 | 4 |
| Q9CZU3 | Skiv2l2 PE | Superkiller viralicidic activity 2-like 2 OS | 2.02 | 4 |
| Q8BFR5 | Tufm PE | Elongation factor Tu, mitochondrial OS | 3.02 | 2 |
| Q9JJI8 | Rpl38 PE | 60S ribosomal protein L38 OS | 2.02 | 4 |
| Q8VHX6 | Flnc PE | Filamin-C OS | 1.01 | 2 |
| Q61701 | Elavl4 PE | ELAV-like protein 4 OS | 5.04 | 5 |
| Q9R1P3 | Psmb2 PE | Proteasome subunit beta type-2 OS | 7.06 | 5 |
| Q9CR57 | Rpl14 PE | 60S ribosomal protein L14 OS | 19.15 | 25 |
| P63028 | Tpt1 PE | Translationally-controlled tumor protein OS | 10.08 | 9 |
| Q9D7G0 | Prps1 PE | Ribose-phosphate pyrophosphokinase 1 OS | 4.03 | 2 |
| P84104 | Sfrs3 PE | Splicing factor, arginine/serine-rich 3 OS | 14.11 | 8 |
| Q64433 | Hspe1 PE | 10 kDa heat shock protein, mitochondrial OS | 6.05 | 7 |
| P59999 | Arpc4 PE | Actin-related protein 2/3 complex subunit 4 OS | 8.06 | 10 |
| Q9ER72 | Cars PE | Cysteinyl-tRNA synthetase, cytoplasmic OS | 4.03 | 2 |
| Q9DBP5 | Cmpk1 PE | UMP-CMP kinase OS | 5.04 | 2 |
| O54774 | Ap3d1 PE | AP-3 complex subunit delta-1 OS | 1.01 | 3 |
| Q61686 | Cbx5 PE | Chromobox protein homolog 5 OS | 4.03 | 6 |
| P45376 | Akr1b1 PE | Aldose reductase OS | 7.06 | 9 |
| P01942 | Hba PE | Hemoglobin subunit alpha OS | 1.01 | 0 |
| Q9WV54 | Asah1 PE | Acid ceramidase OS | 0 | 1 |
| P11103 | Parp1 PE | Poly [ADP-ribose] polymerase 1 OS | 2.02 | 4 |
| O88844 | Idh1 PE | Isocitrate dehydrogenase [NADP] cytoplasmic OS | 2.02 | 3 |
| Q8VBV7 | Cops8 PE | COP9 signalosome complex subunit 8 OS | 4.03 | 2 |
| Q99P88 | Nup155 PE | Nuclear pore complex protein Nup155 OS | 3.02 | 2 |
| Q8C854 | Myef2 PE | Myelin expression factor 2 OS | 3.02 | 4 |
| Q9QYI3 | Dnajc7 PE | DnaJ homolog subfamily C member 7 OS | 5.04 | 4 |
| O08749 | Dld PE | Dihydrolipoyl dehydrogenase, mitochondrial OS | 3.02 | 2 |
| Q9QZE7 | Tsnax PE | Translin-associated protein X OS | 3.02 | 1 |
| Q64152 | Btf3 PE | Transcription factor BTF3 OS | 7.06 | 3 |
| Q80UG5 | Sept9 PE | Septin-9 OS | 2.02 | 3 |
| Q01768 | Nme2 PE | Nucleoside diphosphate kinase B OS | 13.1 | 13 |
| P32921 | Wars PE | Tryptophanyl-tRNA synthetase, cytoplasmic OS | 5.04 | 0 |
| Q9CPR4 | Rpl17 PE | 60S ribosomal protein L17 OS | 7.06 | 7 |
| Q9CPW4 | Arpc5 PE | Actin-related protein 2/3 complex subunit 5 OS | 2.02 | 5 |
| O35841 | Api5 PE | Apoptosis inhibitor 5 OS | 4.03 | 2 |
| O35857 | Timm44 PE | Mitochondrial import inner membrane translocase subunit TIM44 OS | 4.03 | 3 |
| P08030 | Aprt PE | Adenine phosphoribosyltransferase OS | 13.1 | 12 |
| Q3THS6 | Mat2a PE | S-adenosylmethionine synthetase isoform type-2 OS | 6.05 | 3 |
| P08752 | Gnai2 PE | Guanine nucleotide-binding protein G(i), alpha-2 subunit OS | 3.02 | 2 |
| Q9DBC7 | Prkar1a PE | cAMP-dependent protein kinase type I-alpha regulatory subunit OS | 4.03 | 1 |
| Q9R257 | Hebp1 PE | Heme-binding protein 1 OS | 3.02 | 0 |
| Q9D358 | Acp1 PE | Low molecular weight phosphotyrosine protein phosphatase OS | 6.05 | 6 |
| P08228 | Sod1 PE | Superoxide dismutase [Cu-Zn] OS | 14.11 | 13 |
| P19783 | Cox4i1 PE | Cytochrome c oxidase subunit 4 isoform 1, mitochondrial OS | 2.02 | 1 |
| P63276 | Rps17 PE | 40S ribosomal protein S17 OS | 3.02 | 7 |
| P24527 | Lta4h PE | Leukotriene A-4 hydrolase OS | 0 | 2 |
| P62264 | Rps14 PE | 40S ribosomal protein S14 OS | 11.09 | 13 |
| Q6ZWV3 | Rpl10 PE | 60S ribosomal protein L10 OS | 6.05 | 8 |
| P84084 | Arf5 PE | ADP-ribosylation factor 5 OS | 8.06 | 10 |
| P48722 | Hspa4l PE | Heat shock 70 kDa protein 4L OS | 4.03 | 1 |
| Q9JIF7 | Copb1 PE | Coatomer subunit beta OS | 3.02 | 2 |
| Q9JJU8 | Sh3bgrl PE | SH3 domain-binding glutamic acid-rich-like protein OS | 2.02 | 1 |
| Q9DB15 | Mrpl12 PE | 39S ribosomal protein L12, mitochondrial OS | 8.06 | 7 |
| Q8R5C5 | Actr1b PE | Beta-centractin OS | 4.03 | 3 |
| P54728 | Rad23b PE | UV excision repair protein RAD23 homolog B OS | 1.01 | 2 |
| Q61081 | Cdc37 PE | Hsp90 co-chaperone Cdc37 OS | 5.04 | 2 |
| Q9JK81 | Myg1 PE | UPF0160 protein MYG1, mitochondrial OS | 4.03 | 1 |
| Q99LE6 | Abcf2 PE | ATP-binding cassette sub-family F member 2 OS | 5.04 | 0 |
| Q924C1 | Xpo5 PE | Exportin-5 OS | 2.02 | 3 |
| Q64522 | Hist2h2ab PE | Histone H2A type 2-B OS | 123.98 | 62 |
| Q9CVB6 | Arpc2 PE | Actin-related protein 2/3 complex subunit 2 OS | 5.04 | 3 |
| O70311 | Nmt2 PE | Glycylpeptide N-tetradecanoyltransferase 2 OS | 3.02 | 2 |
| Q501J6 | Ddx17 PE | Probable ATP-dependent RNA helicase DDX17 OS | 13.1 | 15 |
| P24288 | Bcat1 PE | Branched-chain-amino-acid aminotransferase, cytosolic OS | 4.03 | 5 |
| Q8CAY6 | Acat2 PE | Acetyl-CoA acetyltransferase, cytosolic OS | 2.02 | 4 |
| Q9EST5 | Anp32b PE | Acidic leucine-rich nuclear phosphoprotein 32 family member B OS | 2.02 | 7 |
| Q9DAR7 | Dcps PE | Scavenger mRNA-decapping enzyme DcpS OS | 3.02 | 2 |
| Q9Z130 | Hnrpdl PE | Heterogeneous nuclear ribonucleoprotein D-like OS | 5.04 | 5 |
| Q8BIJ6 | Iars2 PE | Isoleucyl-tRNA synthetase, mitochondrial OS | 1.01 | 0 |
| P62245 | Rps15a PE | 40S ribosomal protein S15a OS | 6.05 | 4 |
| P62717 | Rpl18a PE | 60S ribosomal protein L18a OS | 8.06 | 7 |
| Q6A068 | Cdc5l PE | Cell division cycle 5-related protein OS | 4.03 | 1 |
| Q99JB2 | Stoml2 PE | Stomatin-like protein 2 OS | 5.04 | 2 |
| O55201 | Supt5h PE | Transcription elongation factor SPT5 OS | 3.02 | 1 |
| P47857 | Pfkm PE | 6-phosphofructokinase, muscle type OS | 4.03 | 0 |
| Q99KQ4 | Nampt PE | Nicotinamide phosphoribosyltransferase OS | 2.02 | 4 |
| P10639 | Txn PE | Thioredoxin OS | 8.06 | 14 |
| Q7TNV0 | Dek PE | Protein DEK OS | 4.03 | 0 |
| Q99MD9 | Nasp PE | Nuclear autoantigenic sperm protein OS | 1.01 | 4 |
| Q8C7X2 | Kiaa0090 PE | Uncharacterized protein KIAA0090 OS | 3.02 | 1 |
| Q9R0P5 | Dstn PE | Destrin OS | 4.03 | 7 |
| Q91V41 | Rab14 PE | Ras-related protein Rab-14 OS | 3.02 | 4 |
| Q9CQM9 | Glrx3 PE | Glutaredoxin-3 OS | 4.03 | 5 |
| Q9WUM4 | Coro1c PE | Coronin-1C OS | 3.02 | 5 |
| P35293 | Rab18 PE | Ras-related protein Rab-18 OS | 5.04 | 3 |
| Q6PHZ2 | Camk2d PE | Calcium/calmodulin-dependent protein kinase type II delta chain OS | 0 | 1 |
| O70492 | Snx3 PE | Sorting nexin-3 OS | 7.06 | 3 |
| Q91W50 | Csde1 PE | Cold shock domain-containing protein E1 OS | 3.02 | 0 |
| Q8VH51 | Rbm39 PE | RNA-binding protein 39 OS | 0 | 4 |
| Q9QUM9 | Psma6 PE | Proteasome subunit alpha type-6 OS | 6.05 | 7 |
| Q99JR1 | Sfxn1 PE | Sideroflexin-1 OS | 2.02 | 5 |
| Q9QZQ1 | Mllt4 PE | Afadin OS | 1.01 | 3 |
| P39054 | Dnm2 PE | Dynamin-2 OS | 2.02 | 0 |
| Q9CQ80 | Vps25 PE | Vacuolar protein-sorting-associated protein 25 OS | 5.04 | 5 |
| Q9JJ80 | Bxdc1 PE | Brix domain-containing protein 1 OS | 4.03 | 2 |
| Q7M6Y3 | Picalm PE | Phosphatidylinositol-binding clathrin assembly protein OS | 1.01 | 3 |
| Q9R0U0 | Fusip1 PE | FUS-interacting serine-arginine-rich protein 1 OS | 11.09 | 4 |
| P32020 | Scp2 PE | Non-specific lipid-transfer protein OS | 0 | 1 |
| P62843 | Rps15 PE | 40S ribosomal protein S15 OS | 2.02 | 4 |
| Q9Z1D1 | Eif3g PE | Eukaryotic translation initiation factor 3 subunit G OS | 5.04 | 3 |
| Q9QYJ0 | Dnaja2 PE | DnaJ homolog subfamily A member 2 OS | 4.03 | 0 |
| Q9Z1Z2 | Strap PE | Serine-threonine kinase receptor-associated protein OS | 3.02 | 1 |
| Q62383 | Supt6h PE | Transcription elongation factor SPT6 OS | 1.01 | 2 |
| P62317 | Snrpd2 PE | Small nuclear ribonucleoprotein Sm D2 OS | 4.03 | 3 |
| Q9D2G2 | Dlst PE | Dihydrolipoyllysine-residue succinyltransferase component of 2-oxoglutarate dehydrogenase complex, mitochondrial OS | 3.02 | 2 |
| Q62186 | Ssr4 PE | Translocon-associated protein subunit delta OS | 6.05 | 1 |
| O09106 | Hdac1 PE | Histone deacetylase 1 OS | 4.03 | 1 |
| Q9DCS9 | Ndufb10 PE | NADH dehydrogenase [ubiquinone] 1 beta subcomplex subunit 10 OS | 5.04 | 0 |
| Q60972 | Rbbp4 PE | Histone-binding protein RBBP4 OS | 4.03 | 3 |
| Q6P1B1 | Xpnpep1 PE | Xaa-Pro aminopeptidase 1 OS | 1.01 | 3 |
| P62900 | Rpl31 PE | 60S ribosomal protein L31 OS | 9.07 | 11 |
| Q9Z0W3 | Nup160 PE | Nuclear pore complex protein Nup160 OS | 1.01 | 3 |
| Q9D1D4 | Tmed10 PE | Transmembrane emp24 domain-containing protein 10 OS | 4.03 | 2 |
| Q99LP6 | Grpel1 PE | GrpE protein homolog 1, mitochondrial OS | 3.02 | 4 |
| P62315 | Snrpd1 PE | Small nuclear ribonucleoprotein Sm D1 OS | 7.06 | 5 |
| Q9JLJ2 | Aldh9a1 PE | 4-trimethylaminobutyraldehyde dehydrogenase OS | 1.01 | 4 |
| P58389 | Ppp2r4 PE | Serine/threonine-protein phosphatase 2A regulatory subunit B' OS | 4.03 | 2 |
| Q9Z1F9 | Uba2 PE | SUMO-activating enzyme subunit 2 OS | 3.02 | 1 |
| Q6IFX2 | Krt42 PE | Keratin, type I cytoskeletal 42 OS | 0 | 6 |
| Q9Z1Q5 | Clic1 PE | Chloride intracellular channel protein 1 OS | 6.05 | 6 |
| O35226 | Psmd4 PE | 26S proteasome non-ATPase regulatory subunit 4 OS | 1.01 | 2 |
| Q8BL97 | Sfrs7 PE | Splicing factor, arginine/serine-rich 7 OS | 13.1 | 9 |
| P60867 | Rps20 PE | 40S ribosomal protein S20 OS | 5.04 | 10 |
| Q9R1C7 | Prpf40a PE | Pre-mRNA-processing factor 40 homolog A OS | 0 | 5 |
| Q80UM7 | Mogs PE | Mannosyl-oligosaccharide glucosidase OS | 2.02 | 1 |
| Q62348 | Tsn PE | Translin OS | 1.01 | 2 |
| Q9ESZ8 | Gtf2i PE | General transcription factor II-I OS | 0 | 3 |
| P60824 | Cirbp PE | Cold-inducible RNA-binding protein OS | 3.02 | 2 |
| Q61239 | Fnta PE | Protein farnesyltransferase/geranylgeranyltransferase type-1 subunit alpha OS | 2.02 | 3 |
| P05063 | Aldoc PE | Fructose-bisphosphate aldolase C OS | 1.01 | 0 |
| Q04750 | Top1 PE | DNA topoisomerase 1 OS | 4.03 | 0 |
| Q9JKX4 | Aatf PE | Protein AATF OS | 0 | 3 |
| O09172 | Gclm PE | Glutamate--cysteine ligase regulatory subunit OS | 1.01 | 1 |
| Q9QXB9 | Drg2 PE | Developmentally-regulated GTP-binding protein 2 OS | 3.02 | 2 |
| P58771 | Tpm1 PE | Tropomyosin alpha-1 chain OS | 4.03 | 4 |
| Q8CGF7 | Tcerg1 PE | Transcription elongation regulator 1 OS | 2.02 | 1 |
| P32233 | Drg1 PE | Developmentally-regulated GTP-binding protein 1 OS | 3.02 | 3 |
| Q61990 | Pcbp2 PE | Poly(rC)-binding protein 2 OS | 8.06 | 9 |
| P35486 | Pdha1 PE | Pyruvate dehydrogenase E1 component subunit alpha, somatic form, mitochondrial OS | 3.02 | 0 |
| Q9D051 | Pdhb PE | Pyruvate dehydrogenase E1 component subunit beta, mitochondrial OS | 0 | 2 |
| Q8K019 | Bclaf1 PE | Bcl-2-associated transcription factor 1 OS | 3.02 | 2 |
| P35979 | Rpl12 PE | 60S ribosomal protein L12 OS | 16.13 | 6 |
| Q9JMH6 | Txnrd1 PE | Thioredoxin reductase 1, cytoplasmic OS | 3.02 | 1 |
| P62849 | Rps24 PE | 40S ribosomal protein S24 OS | 6.05 | 8 |
| Q4VA53 | Pds5b PE | Sister chromatid cohesion protein PDS5 homolog B OS | 3.02 | 1 |
| Q9WUA2 | Farsb PE | Phenylalanyl-tRNA synthetase beta chain OS | 3.02 | 2 |
| P56812 | Pdcd5 PE | Programmed cell death protein 5 OS | 6.05 | 2 |
| Q6ZQ73 | Cand2 PE | Cullin-associated NEDD8-dissociated protein 2 OS | 1.01 | 3 |
| P16045 | Lgals1 PE | Galectin-1 OS | 1.01 | 3 |
| Q9CZR8 | Tsfm PE | Elongation factor Ts, mitochondrial OS | 1.01 | 2 |
| Q3U9G9 | Lbr PE | Lamin-B receptor OS | 0 | 4 |
| P84099 | Rpl19 PE | 60S ribosomal protein L19 OS | 5.04 | 6 |
| Q91YR1 | Twf1 PE | Twinfilin-1 OS | 3.02 | 2 |
| Q569Z6 | Thrap3 PE | Thyroid hormone receptor-associated protein 3 OS | 2.02 | 1 |
| Q9Z0S1 | Bpnt1 PE | 3'(2'),5'-bisphosphate nucleotidase 1 OS | 3.02 | 4 |
| Q8BJU0 | Sgta PE | Small glutamine-rich tetratricopeptide repeat-containing protein alpha OS | 2.02 | 2 |
| Q62418 | Dbnl PE | Drebrin-like protein OS | 2.02 | 1 |
| P28352 | Apex1 PE | DNA-(apurinic or apyrimidinic site) lyase OS | 2.02 | 4 |
| Q9WUL7 | Arl3 PE | ADP-ribosylation factor-like protein 3 OS | 3.02 | 5 |
| Q9CZ04 | Cops7a PE | COP9 signalosome complex subunit 7a OS | 4.03 | 2 |
| P47758 | Srprb PE | Signal recognition particle receptor subunit beta OS | 3.02 | 0 |
| Q61414 | Krt15 PE | Keratin, type I cytoskeletal 15 OS | 1.01 | 9 |
| Q9JKX6 | Nudt5 PE | ADP-sugar pyrophosphatase OS | 5.04 | 4 |
| Q9CZY3 | Ube2v1 PE | Ubiquitin-conjugating enzyme E2 variant 1 OS | 4.03 | 7 |
| Q8R0G9 | Nup133 PE | Nuclear pore complex protein Nup133 OS | 4.03 | 2 |
| Q3U1J4 | Ddb1 PE | DNA damage-binding protein 1 OS | 0 | 4 |
| P70288 | Hdac2 PE | Histone deacetylase 2 OS | 4.03 | 1 |
| Q9WU78 | Pdcd6ip PE | Programmed cell death 6-interacting protein OS | 2.02 | 1 |
| Q8BVI4 | Qdpr PE | Dihydropteridine reductase OS | 0 | 3 |
| Q9JKB1 | Uchl3 PE | Ubiquitin carboxyl-terminal hydrolase isozyme L3 OS | 3.02 | 4 |
| Q7TMY8 | Huwe1 PE | E3 ubiquitin-protein ligase HUWE1 OS | 1.01 | 3 |
| Q6NVF9 | Cpsf6 PE | Cleavage and polyadenylation specificity factor subunit 6 OS | 3.02 | 2 |
| P21550 | Eno3 PE | Beta-enolase OS | 18.14 | 18 |
| Q99LD8 | Ddah2 PE | N(G),N(G)-dimethylarginine dimethylaminohydrolase 2 OS | 1.01 | 1 |
| Q9Z1G3 | Atp6v1c1 PE | V-type proton ATPase subunit C 1 OS | 2.02 | 3 |
| Q9WUK2 | Eif4h PE | Eukaryotic translation initiation factor 4H OS | 3.02 | 3 |
| P46460 | Nsf PE | Vesicle-fusing ATPase OS | 3.02 | 2 |
| O09167 | Rpl21 PE | 60S ribosomal protein L21 OS | 8.06 | 8 |
| Q91ZJ5 | Ugp2 PE | UTP--glucose-1-phosphate uridylyltransferase OS | 2.02 | 0 |
| P46664 | Adss PE | Adenylosuccinate synthetase isozyme 2 OS | 1.01 | 2 |
| Q9QY76 | Vapb PE | Vesicle-associated membrane protein-associated protein B OS | 4.03 | 2 |
| P04104 | Krt1 PE | Keratin, type II cytoskeletal 1 OS | 17.13 | 35 |
| Q91YR7 | Prpf6 PE | Pre-mRNA-processing factor 6 OS | 3.02 | 0 |
| Q9CS42 | Prps2 PE | Ribose-phosphate pyrophosphokinase 2 OS | 3.02 | 2 |
| P61087 | Ube2k PE | Ubiquitin-conjugating enzyme E2 K OS | 3.02 | 5 |
| Q8CDN6 | Txnl1 PE | Thioredoxin-like protein 1 OS | 2.02 | 1 |
| P46978 | Stt3a PE | Dolichyl-diphosphooligosaccharide--protein glycosyltransferase subunit STT3A OS | 0 | 7 |
| Q9CSU0 | Rprd1b PE | Regulation of nuclear pre-mRNA domain-containing protein 1B OS | 2.02 | 2 |
| P50431 | Shmt1 PE | Serine hydroxymethyltransferase, cytosolic OS | 3.02 | 2 |
| Q3TCN2 | Plbd2 PE | Putative phospholipase B-like 2 OS | 0 | 1 |
| Q9DAW9 | Cnn3 PE | Calponin-3 OS | 2.02 | 3 |
| O08547 | Sec22b PE | Vesicle-trafficking protein SEC22b OS | 2.02 | 1 |
| Q9D662 | Sec23b PE | Protein transport protein Sec23B OS | 3.02 | 1 |
| Q6PDI5 | Ecm29 PE | Proteasome-associated protein ECM29 homolog OS | 2.02 | 6 |
| Q9JJV2 | Pfn2 PE | Profilin-2 OS | 1.01 | 0 |
| P61358 | Rpl27 PE | 60S ribosomal protein L27 OS | 4.03 | 5 |
| P62869 | Tceb2 PE | Transcription elongation factor B polypeptide 2 OS | 4.03 | 9 |
| P20108 | Prdx3 PE | Thioredoxin-dependent peroxide reductase, mitochondrial OS | 6.05 | 5 |
| Q07813 | Bax PE | Apoptosis regulator BAX OS | 3.02 | 3 |
| Q99KV1 | Dnajb11 PE | DnaJ homolog subfamily B member 11 OS | 1.01 | 2 |
| P62918 | Rpl8 PE | 60S ribosomal protein L8 OS | 9.07 | 12 |
| P31786 | Dbi PE | Acyl-CoA-binding protein OS | 0 | 2 |
| Q9JLI8 | Sart3 PE | Squamous cell carcinoma antigen recognized by T-cells 3 OS | 1.01 | 3 |
| Q8BWY3 | Etf1 PE | Eukaryotic peptide chain release factor subunit 1 OS | 5.04 | 1 |
| P47754 | Capza2 PE | F-actin-capping protein subunit alpha-2 OS | 2.02 | 4 |
| P24547 | Impdh2 PE | Inosine-5'-monophosphate dehydrogenase 2 OS | 2.02 | 4 |
| P70122 | Sbds PE | Ribosome maturation protein SBDS OS | 3.02 | 0 |
| P62267 | Rps23 PE | 40S ribosomal protein S23 OS | 7.06 | 11 |
| Q505F5 | Lrrc47 PE | Leucine-rich repeat-containing protein 47 OS | 4.03 | 4 |
| Q07646 | Mest PE | Mesoderm-specific transcript protein OS | 3.02 | 2 |
| P10922 | H1f0 PE | Histone H1.0 OS | 3.02 | 10 |
| O70591 | Pfdn2 PE | Prefoldin subunit 2 OS | 2.02 | 4 |
| P70398 | Usp9x PE | Probable ubiquitin carboxyl-terminal hydrolase FAF-X OS | 0 | 2 |
| Q9DBG7 | Srpr PE | Signal recognition particle receptor subunit alpha OS | 3.02 | 1 |
| O89017 | Lgmn PE | Legumain OS | 3.02 | 1 |
| P62852 | Rps25 PE | 40S ribosomal protein S25 OS | 14.11 | 14 |
| O70318 | Epb41l2 PE | Band 4.1-like protein 2 OS | 0 | 1 |
| Q61425 | Hadh PE | Hydroxyacyl-coenzyme A dehydrogenase, mitochondrial OS | 3.02 | 2 |
| P62897 | Cycs PE | Cytochrome c, somatic OS | 1.01 | 1 |
| Q925I1 | Atad3 PE | ATPase family AAA domain-containing protein 3 OS | 3.02 | 0 |
| P61750 | Arf4 PE | ADP-ribosylation factor 4 OS | 3.02 | 3 |
| Q61035 | Hars PE | Histidyl-tRNA synthetase, cytoplasmic OS | 3.02 | 0 |
| P54822 | Adsl PE | Adenylosuccinate lyase OS | 1.01 | 2 |
| O35134 | Polr1a PE | DNA-directed RNA polymerase I subunit RPA1 OS | 0 | 3 |
| P97461 | Rps5 PE | 40S ribosomal protein S5 OS | 4.03 | 0 |
| Q9D1C1 | Ube2c PE | Ubiquitin-conjugating enzyme E2 C OS | 4.03 | 1 |
| Q6ZQL4 | Wdr43 PE | WD repeat-containing protein 43 OS | 3.02 | 0 |
| Q9JMA1 | Usp14 PE | Ubiquitin carboxyl-terminal hydrolase 14 OS | 4.03 | 3 |
| P00405 | Mtco2 PE | Cytochrome c oxidase subunit 2 OS | 0 | 1 |
| Q9DCL9 | Paics PE | Multifunctional protein ADE2 OS | 4.03 | 4 |
| P63024 | Vamp3 PE | Vesicle-associated membrane protein 3 OS | 2.02 | 5 |
| P70698 | Ctps PE | CTP synthase 1 OS | 0 | 4 |
| P17439 | Gba PE | Glucosylceramidase OS | 1.01 | 1 |
| P70677 | Casp3 PE | Caspase-3 OS | 2.02 | 8 |
| P62889 | Rpl30 PE | 60S ribosomal protein L30 OS | 5.04 | 3 |
| P63280 | Ube2i PE | SUMO-conjugating enzyme UBC9 OS | 2.02 | 3 |
| Q9JM76 | Arpc3 PE | Actin-related protein 2/3 complex subunit 3 OS | 7.06 | 4 |
| P48758 | Cbr1 PE | Carbonyl reductase [NADPH] 1 OS | 1.01 | 3 |
| Q791V5 | Mtch2 PE | Mitochondrial carrier homolog 2 OS | 4.03 | 0 |
| Q9CPQ8 | Atp5l PE | ATP synthase subunit g, mitochondrial OS | 1.01 | 3 |
| Q9D1G1 | Rab1b PE | Ras-related protein Rab-1B OS | 9.07 | 4 |
| Q9R0E1 | Plod3 PE | Procollagen-lysine,2-oxoglutarate 5-dioxygenase 3 OS | 3.02 | 1 |
| Q99LG2 | Tnpo2 PE | Transportin-2 OS | 3.02 | 2 |
| P00493 | Hprt1 PE | Hypoxanthine-guanine phosphoribosyltransferase OS | 1.01 | 6 |
| Q6ZWV7 | Rpl35 PE | 60S ribosomal protein L35 OS | 8.06 | 8 |
| Q6PD26 | Pigs PE | GPI transamidase component PIG-S OS | 2.02 | 2 |
| Q62093 | Sfrs2 PE | Splicing factor, arginine/serine-rich 2 OS | 2.02 | 5 |
| Q9D0J4 | Arl2 PE | ADP-ribosylation factor-like protein 2 OS | 3.02 | 3 |
| O88487 | Dync1i2 PE | Cytoplasmic dynein 1 intermediate chain 2 OS | 2.02 | 0 |
| Q60749 | Khdrbs1 PE | KH domain-containing, RNA-binding, signal transduction-associated protein 1 OS | 0 | 2 |
| P45591 | Cfl2 PE | Cofilin-2 OS | 12.1 | 11 |
| Q8K0C4 | Cyp51a1 PE | Lanosterol 14-alpha demethylase OS | 2.02 | 3 |
| Q9QYR6 | Map1a PE | Microtubule-associated protein 1A OS | 2.02 | 2 |
| P12787 | Cox5a PE | Cytochrome c oxidase subunit 5A, mitochondrial OS | 7.06 | 5 |
| Q9CRB6 | Tppp3 PE | Tubulin polymerization-promoting protein family member 3 OS | 7.06 | 2 |
| Q9CZW5 | Tomm70a PE | Mitochondrial import receptor subunit TOM70 OS | 1.01 | 1 |
| Q61792 | Lasp1 PE | LIM and SH3 domain protein 1 OS | 0 | 1 |
| Q921M7 | Fam49b PE | Protein FAM49B OS | 0 | 5 |
| Q9R1Q8 | Tagln3 PE | Transgelin-3 OS | 2.02 | 1 |
| Q9ERU9 | Ranbp2 PE | E3 SUMO-protein ligase RanBP2 OS | 1.01 | 4 |
| Q9QYF1 | Rdh11 PE | Retinol dehydrogenase 11 OS | 1.01 | 6 |
| P28740 | Kif2a PE | Kinesin-like protein KIF2A OS | 3.02 | 0 |
| P57759 | Erp29 PE | Endoplasmic reticulum protein ERp29 OS | 2.02 | 5 |
| P35279 | Rab6a PE | Ras-related protein Rab-6A OS | 3.02 | 2 |
| P11031 | Sub1 PE | Activated RNA polymerase II transcriptional coactivator p15 OS | 3.02 | 2 |
| P21995 | Emb PE | Embigin OS | 3.02 | 4 |
| P21278 | Gna11 PE | Guanine nucleotide-binding protein subunit alpha-11 OS | 0 | 1 |
| P10630 | Eif4a2 PE | Eukaryotic initiation factor 4A-II OS | 2.02 | 3 |
| Q6ZWU9 | Rps27 PE | 40S ribosomal protein S27 OS | 3.02 | 4 |
| P61089 | Ube2n PE | Ubiquitin-conjugating enzyme E2 N OS | 7.06 | 8 |
| O08599 | Stxbp1 PE | Syntaxin-binding protein 1 OS | 3.02 | 1 |
| P62774 | Mtpn PE | Myotrophin OS | 2.02 | 3 |
| Q3UHX2 | Pdap1 PE | 28 kDa heat- and acid-stable phosphoprotein OS | 1.01 | 3 |
| Q6P1F6 | Ppp2r2a PE | Serine/threonine-protein phosphatase 2A 55 kDa regulatory subunit B alpha isoform OS | 3.02 | 1 |
| Q5XJY5 | Arcn1 PE | Coatomer subunit delta OS | 1.01 | 1 |
| O09061 | Psmb1 PE | Proteasome subunit beta type-1 OS | 3.02 | 6 |
| Q9QUI0 | Rhoa PE | Transforming protein RhoA OS | 2.02 | 4 |
| Q9DBR1 | Xrn2 PE | 5'-3' exoribonuclease 2 OS | 1.01 | 0 |
| Q9D0M3 | Cyc1 PE | Cytochrome c1, heme protein, mitochondrial OS | 4.03 | 1 |
| Q6NS46 | Pdcd11 PE | Protein RRP5 homolog OS | 2.02 | 3 |
| O70503 | Hsd17b12 PE | Estradiol 17-beta-dehydrogenase 12 OS | 1.01 | 3 |
| P11679 | Krt8 PE | Keratin, type II cytoskeletal 8 OS | 4.03 | 1 |
| Q99LI7 | Cstf3 PE | Cleavage stimulation factor 77 kDa subunit OS | 3.02 | 0 |
| Q8C0C7 | Farsa PE | Phenylalanyl-tRNA synthetase alpha chain OS | 1.01 | 3 |
| O35114 | Scarb2 PE | Lysosome membrane protein 2 OS | 1.01 | 0 |
| A2APV2 | Fmnl2 PE | Formin-like protein 2 OS | 0 | 2 |
| O35864 | Cops5 PE | COP9 signalosome complex subunit 5 OS | 2.02 | 1 |
| Q05816 | Fabp5 PE | Fatty acid-binding protein, epidermal OS | 4.03 | 4 |
| P47753 | Capza1 PE | F-actin-capping protein subunit alpha-1 OS | 2.02 | 4 |
| Q6NZB0 | Dnajc8 PE | DnaJ homolog subfamily C member 8 OS | 2.02 | 1 |
| Q99KP6 | Prpf19 PE | Pre-mRNA-processing factor 19 OS | 2.02 | 3 |
| O35900 | Lsm2 PE | U6 snRNA-associated Sm-like protein LSm2 OS | 1.01 | 2 |
| Q9CX56 | Psmd8 PE | 26S proteasome non-ATPase regulatory subunit 8 OS | 0 | 3 |
| Q3UM45 | Ppp1r7 PE | Protein phosphatase 1 regulatory subunit 7 OS | 2.02 | 4 |
| Q8VE97 | Sfrs4 PE | Splicing factor, arginine/serine-rich 4 OS | 3.02 | 0 |
| O08756 | Hsd17b10 PE | 3-hydroxyacyl-CoA dehydrogenase type-2 OS | 2.02 | 2 |
| Q9Z0N1 | Eif2s3x PE | Eukaryotic translation initiation factor 2 subunit 3, X-linked OS | 3.02 | 2 |
| Q9DBD5 | Pelp1 PE | Proline-, glutamic acid- and leucine-rich protein 1 OS | 2.02 | 3 |
| Q6NZC7 | Sec23ip PE | SEC23-interacting protein OS | 2.02 | 1 |
| P08553 | Nefm PE | Neurofilament medium polypeptide OS | 1.01 | 0 |
| P47757 | Capzb PE | F-actin-capping protein subunit beta OS | 2.02 | 5 |
| Q66JS6 | Eif3j PE | Eukaryotic translation initiation factor 3 subunit J OS | 3.02 | 2 |
| O08583 | Thoc4 PE | THO complex subunit 4 OS | 5.04 | 7 |
| Q64520 | Guk1 PE | Guanylate kinase OS | 1.01 | 2 |
| Q9DC61 | Pmpca PE | Mitochondrial-processing peptidase subunit alpha OS | 0 | 3 |
| Q8BKS9 | Kiaa0020 PE | Pumilio domain-containing protein KIAA0020 OS | 3.02 | 0 |
| P57784 | Snrpa1 PE | U2 small nuclear ribonucleoprotein A' OS | 3.02 | 3 |
| P61082 | Ube2m PE | NEDD8-conjugating enzyme Ubc12 OS | 0 | 5 |
| Q5SW19 | Kiaa0664 PE | Protein KIAA0664 OS | 2.02 | 2 |
| O35479 | Rbmx PE | Heterogeneous nuclear ribonucleoprotein G OS | 3.02 | 4 |
| P22892 | Ap1g1 PE | AP-1 complex subunit gamma-1 OS | 2.02 | 1 |
| Q9R1T4 | Sept6 PE | Septin-6 OS | 3.02 | 0 |
| O35972 | Mrpl23 PE | 39S ribosomal protein L23, mitochondrial OS | 1.01 | 1 |
| Q8WTY4 | Ciapin1 PE | Anamorsin OS | 3.02 | 1 |
| Q62425 | Ndufa4 PE | NADH dehydrogenase [ubiquinone] 1 alpha subcomplex subunit 4 OS | 1.01 | 2 |
| Q9D7X3 | Dusp3 PE | Dual specificity protein phosphatase 3 OS | 1.01 | 2 |
| P09055 | Itgb1 PE | Integrin beta-1 OS | 1.01 | 1 |
| Q91YP2 | Nln PE | Neurolysin, mitochondrial OS | 2.02 | 0 |
| Q9CQR2 | Rps21 PE | 40S ribosomal protein S21 OS | 2.02 | 1 |
| P62281 | Rps11 PE | 40S ribosomal protein S11 OS | 5.04 | 6 |
| Q61166 | Mapre1 PE | Microtubule-associated protein RP/EB family member 1 OS | 0 | 2 |
| P63001 | Rac1 PE | Ras-related C3 botulinum toxin substrate 1 OS | 3.02 | 3 |
| Q9Z2K1 | Krt16 PE | Keratin, type I cytoskeletal 16 OS | 1.01 | 6 |
| Q9WTX5 | Skp1 PE | S-phase kinase-associated protein 1 OS | 3.02 | 6 |
| P57746 | Atp6v1d PE | V-type proton ATPase subunit D OS | 2.02 | 2 |
| Q8N9S3 | Ahsa2 PE | Activator of 90 kDa heat shock protein ATPase homolog 2 OS | 2.02 | 2 |
| Q05CL8 | Larp7 PE | La-related protein 7 OS | 3.02 | 0 |
| P40124 | Cap1 PE | Adenylyl cyclase-associated protein 1 OS | 2.02 | 1 |
| Q9Z0H1 | Wdr46 PE | WD repeat-containing protein 46 OS | 2.02 | 0 |
| Q91VK1 | Bzw2 PE | Basic leucine zipper and W2 domain-containing protein 2 OS | 1.01 | 2 |
| O88545 | Cops6 PE | COP9 signalosome complex subunit 6 OS | 2.02 | 2 |
| O35593 | Psmd14 PE | 26S proteasome non-ATPase regulatory subunit 14 OS | 0 | 3 |
| Q8K4Z3 | Apoa1bp PE | Apolipoprotein A-I-binding protein OS | 2.02 | 1 |
| Q8BY87 | Usp47 PE | Ubiquitin carboxyl-terminal hydrolase 47 OS | 0 | 2 |
| Q3UDE2 | Ttll12 PE | Tubulin--tyrosine ligase-like protein 12 OS | 2.02 | 2 |
| Q922J9 | Far1 PE | Fatty acyl-CoA reductase 1 OS | 2.02 | 0 |
| Q9WV92 | Epb41l3 PE | Band 4.1-like protein 3 OS | 1.01 | 3 |
| Q80WJ7 | Mtdh PE | Protein LYRIC OS | 1.01 | 0 |
| Q8C2Q3 | Rbm14 PE | RNA-binding protein 14 OS | 3.02 | 1 |
| P58044 | Idi1 PE | Isopentenyl-diphosphate Delta-isomerase 1 OS | 0 | 2 |
| P23591 | Tsta3 PE | GDP-L-fucose synthetase OS | 2.02 | 3 |
| P09671 | Sod2 PE | Superoxide dismutase [Mn], mitochondrial OS | 3.02 | 2 |
| Q60900 | Elavl3 PE | ELAV-like protein 3 OS | 1.01 | 2 |
| Q9CYG7 | Tomm34 PE | Mitochondrial import receptor subunit TOM34 OS | 2.02 | 1 |
| P47915 | Rpl29 PE | 60S ribosomal protein L29 OS | 6.05 | 10 |
| Q8BH95 | Echs1 PE | Enoyl-CoA hydratase, mitochondrial OS | 2.02 | 2 |
| O55029 | Copb2 PE | Coatomer subunit beta' OS | 1.01 | 2 |
| Q80Y14 | Glrx5 PE | Glutaredoxin-related protein 5 OS | 4.03 | 4 |
| P01900 | H2-D1 PE | H-2 class I histocompatibility antigen, D-D alpha chain OS | 1.01 | 2 |
| Q62448 | Eif4g2 PE | Eukaryotic translation initiation factor 4 gamma 2 OS | 2.02 | 0 |
| Q59J78 | Ndufaf2 PE | Mimitin, mitochondrial OS | 2.02 | 1 |
| Q3UGR5 | Hdhd2 PE | Haloacid dehalogenase-like hydrolase domain-containing protein 2 OS | 3.02 | 1 |
| Q80X82 | Sympk PE | Symplekin OS | 1.01 | 2 |
| P61202 | Cops2 PE | COP9 signalosome complex subunit 2 OS | 3.02 | 0 |
| P50518 | Atp6v1e1 PE | V-type proton ATPase subunit E 1 OS | 3.02 | 4 |
| Q9CY50 | Ssr1 PE | Translocon-associated protein subunit alpha OS | 3.02 | 2 |
| Q9CQ71 | Rpa3 PE | Replication protein A 14 kDa subunit OS | 3.02 | 2 |
| Q8BQM4 | Heatr3 PE | HEAT repeat-containing protein 3 OS | 3.02 | 1 |
| Q8BWZ3 | 1 SV | TPR repeat-containing protein C12orf30 homolog OS | 2.02 | 1 |
| Q9QYJ3 | Dnajb1 PE | DnaJ homolog subfamily B member 1 OS | 1.01 | 2 |
| Q7TT37 | Ikbkap PE | Elongator complex protein 1 OS | 1.01 | 2 |
| Q9JMG7 | Hdgfrp3 PE | Hepatoma-derived growth factor-related protein 3 OS | 0 | 2 |
| P62855 | Rps26 PE | 40S ribosomal protein S26 OS | 7.06 | 5 |
| Q9R0Q7 | Ptges3 PE | Prostaglandin E synthase 3 OS | 6.05 | 3 |
| Q9ER00 | Stx12 PE | Syntaxin-12 OS | 2.02 | 1 |
| P14115 | Rpl27a PE | 60S ribosomal protein L27a OS | 6.05 | 4 |
| Q9CXS4 | Cenpv PE | Centromere protein V OS | 1.01 | 3 |
| P61027 | Rab10 PE | Ras-related protein Rab-10 OS | 3.02 | 2 |
| O35326 | Sfrs5 PE | Splicing factor, arginine/serine-rich 5 OS | 1.01 | 2 |
| Q91WU5 | As3mt PE | Arsenite methyltransferase OS | 1.01 | 2 |
| Q9WVL0 | Gstz1 PE | Maleylacetoacetate isomerase OS | 0 | 1 |
| O54950 | Prkag1 PE | 5'-AMP-activated protein kinase subunit gamma-1 OS | 2.02 | 0 |
| Q9QYA2 | Tomm40 PE | Mitochondrial import receptor subunit TOM40 homolog OS | 2.02 | 1 |
| P10518 | Alad PE | Delta-aminolevulinic acid dehydratase OS | 2.02 | 1 |
| Q8VBT0 | Tmx1 PE | Thioredoxin-related transmembrane protein 1 OS | 2.02 | 2 |
| Q8BP67 | Rpl24 PE | 60S ribosomal protein L24 OS | 5.04 | 9 |
| P70195 | Psmb7 PE | Proteasome subunit beta type-7 OS | 2.02 | 1 |
| Q9D3D9 | Atp5d PE | ATP synthase subunit delta, mitochondrial OS | 4.03 | 5 |
| P70336 | Rock2 PE | Rho-associated protein kinase 2 OS | 0 | 2 |
| Q9CWX9 | Ddx47 PE | Probable ATP-dependent RNA helicase DDX47 OS | 2.02 | 0 |
| Q9DC51 | Gnai3 PE | Guanine nucleotide-binding protein G(k) subunit alpha OS | 3.02 | 1 |
| P31230 | Scye1 PE | Multisynthetase complex auxiliary component p43 OS | 2.02 | 2 |
| Q9QXT0 | Cnpy2 PE | Protein canopy homolog 2 OS | 2.02 | 1 |
| Q91VW3 | Sh3bgrl3 PE | SH3 domain-binding glutamic acid-rich-like protein 3 OS | 0 | 2 |
| Q64105 | Spr PE | Sepiapterin reductase OS | 0 | 1 |
| P47802 | Mtx1 PE | Metaxin-1 OS | 1.01 | 2 |
| P62830 | Rpl23 PE | 60S ribosomal protein L23 OS | 7.06 | 7 |
| Q6P8I4 | Pcnp PE | PEST proteolytic signal-containing nuclear protein OS | 2.02 | 1 |
| Q9CR67 | Tmem33 PE | Transmembrane protein 33 OS | 3.02 | 3 |
| O35343 | Kpna4 PE | Importin subunit alpha-4 OS | 2.02 | 0 |
| Q52KI8 | Srrm1 PE | Serine/arginine repetitive matrix protein 1 OS | 2.02 | 0 |
| Q9DB05 | Napa PE | Alpha-soluble NSF attachment protein OS | 0 | 2 |
| Q8BGW1 | Fto PE | Protein fto OS | 2.02 | 0 |
| Q810A7 | Ddx42 PE | ATP-dependent RNA helicase DDX42 OS | 1.01 | 2 |
| Q8K297 | Glt25d1 PE | Glycosyltransferase 25 family member 1 OS | 2.02 | 0 |
| Q8VED5 | Krt79 PE | Keratin, type II cytoskeletal 79 OS | 8.06 | 15 |
| Q9QZ23 | Nfu1 PE | NFU1 iron-sulfur cluster scaffold homolog, mitochondrial OS | 0 | 2 |
| Q8CG76 | Akr7a2 PE | Aflatoxin B1 aldehyde reductase member 2 OS | 2.02 | 1 |
| Q61074 | Ppm1g PE | Protein phosphatase 1G OS | 3.02 | 2 |
| Q99M31 | Hspa14 PE | Heat shock 70 kDa protein 14 OS | 1.01 | 2 |
| Q8BRF7 | Scfd1 PE | Sec1 family domain-containing protein 1 OS | 1.01 | 2 |
| Q9Z1T1 | Ap3b1 PE | AP-3 complex subunit beta-1 OS | 1.01 | 2 |
| Q60692 | Psmb6 PE | Proteasome subunit beta type-6 OS | 4.03 | 7 |
| Q01730 | Rsu1 PE | Ras suppressor protein 1 OS | 3.02 | 2 |
| Q9CRT8 | Xpot PE | Exportin-T OS | 2.02 | 0 |
| Q9CPQ3 | Tomm22 PE | Mitochondrial import receptor subunit TOM22 homolog OS | 1.01 | 2 |
| Q80ZS3 | Mrps26 PE | 28S ribosomal protein S26, mitochondrial OS | 3.02 | 0 |
| Q9ES00 | Ube4b PE | Ubiquitin conjugation factor E4 B OS | 1.01 | 2 |
| Q99J62 | Rfc4 PE | Replication factor C subunit 4 OS | 2.02 | 0 |
| Q8CCF0 | Prpf31 PE | U4/U6 small nuclear ribonucleoprotein Prp31 OS | 0 | 2 |
| P59708 | Sf3b14 PE | Pre-mRNA branch site protein p14 OS | 1.01 | 3 |
| O35143 | Atpif1 PE | ATPase inhibitor, mitochondrial OS | 1.01 | 1 |
| P61957 | Sumo2 PE | Small ubiquitin-related modifier 2 OS | 6.05 | 5 |
| Q63918 | Sdpr PE | Serum deprivation-response protein OS | 0 | 2 |
| Q9CQ92 | Fis1 PE | Mitochondrial fission 1 protein OS | 0 | 1 |
| Q3TTY5 | Krt2 PE | Keratin, type II cytoskeletal 2 epidermal OS | 1.01 | 3 |
| Q9R1P0 | Psma4 PE | Proteasome subunit alpha type-4 OS | 3.02 | 6 |
| Q9WV80 | Snx1 PE | Sorting nexin-1 OS | 0 | 2 |
| Q9Z2Y8 | Prosc PE | Proline synthetase co-transcribed bacterial homolog protein OS | 1.01 | 4 |
| Q3UVK0 | Ermp1 PE | Endoplasmic reticulum metallopeptidase 1 OS | 0 | 4 |
| Q9D0J8 | Ptms PE | Parathymosin OS | 3.02 | 0 |
| Q9ERR7 | Sep15 PE | 15 kDa selenoprotein OS | 0 | 3 |
| Q9DCA5 | Bxdc2 PE | Brix domain-containing protein 2 OS | 2.02 | 0 |
| Q9CQF3 | Nudt21 PE | Cleavage and polyadenylation specificity factor subunit 5 OS | 2.02 | 3 |
| Q6ZQ58 | Larp1 PE | La-related protein 1 OS | 2.02 | 2 |
| P27601 | Gna13 PE | Guanine nucleotide-binding protein subunit alpha-13 OS | 2.02 | 2 |
| B2RY56 | Rbm25 PE | RNA-binding protein 25 OS | 2.02 | 0 |
| Q99LS3 | Psph PE | Phosphoserine phosphatase OS | 2.02 | 0 |
| O35215 | Ddt PE | D-dopachrome decarboxylase OS | 2.02 | 4 |
| P28667 | Marcksl1 PE | MARCKS-related protein OS | 2.02 | 4 |
| Q80YP0 | Cdk3 PE | Cell division protein kinase 3 OS | 1.01 | 3 |
| Q9DBZ5 | Eif3k PE | Eukaryotic translation initiation factor 3 subunit K OS | 3.02 | 1 |
| P63073 | Eif4e PE | Eukaryotic translation initiation factor 4E OS | 2.02 | 1 |
| Q9D898 | Arpc5l PE | Actin-related protein 2/3 complex subunit 5-like protein OS | 2.02 | 1 |
| Q11136 | Pepd PE | Xaa-Pro dipeptidase OS | 2.02 | 0 |
| P18654 | Rps6ka3 PE | Ribosomal protein S6 kinase alpha-3 OS | 2.02 | 2 |
| O70378 | Cox4nb PE | Neighbor of COX4 OS | 2.02 | 1 |
| P07356 | Anxa2 PE | Annexin A2 OS | 0 | 1 |
| Q9R0E2 | Plod1 PE | Procollagen-lysine,2-oxoglutarate 5-dioxygenase 1 OS | 2.02 | 1 |
| P62858 | Rps28 PE | 40S ribosomal protein S28 OS | 2.02 | 4 |
| Q9CQC9 | Sar1b PE | GTP-binding protein SAR1b OS | 4.03 | 4 |
| Q6PFR5 | Tra2a PE | Transformer-2 protein homolog alpha OS | 3.02 | 1 |
| Q6ZWY3 | Rps27l PE | 40S ribosomal protein S27-like protein OS | 2.02 | 3 |
| Q61550 | Rad21 PE | Double-strand-break repair protein rad21 homolog OS | 2.02 | 0 |
| Q9CQ40 | Mrpl49 PE | 39S ribosomal protein L49, mitochondrial OS | 1.01 | 2 |
| Q8R5H1 | Usp15 PE | Ubiquitin carboxyl-terminal hydrolase 15 OS | 0 | 2 |
| Q9CQX2 | Cyb5b PE | Cytochrome b5 type B OS | 4.03 | 4 |
| Q9CR62 | Slc25a11 PE | Mitochondrial 2-oxoglutarate/malate carrier protein OS | 2.02 | 2 |
| P14576 | Srp54 PE | Signal recognition particle 54 kDa protein OS | 0 | 2 |
| P97315 | Csrp1 PE | Cysteine and glycine-rich protein 1 OS | 2.02 | 2 |
| Q9CWM4 | Pfdn1 PE | Prefoldin subunit 1 OS | 3.02 | 4 |
| Q8K1J6 | Trnt1 PE | tRNA-nucleotidyltransferase 1, mitochondrial OS | 2.02 | 1 |
| P37040 | Por PE | NADPH--cytochrome P450 reductase OS | 2.02 | 0 |
| Q9DCZ4 | Apoo PE | Apolipoprotein O OS | 2.02 | 0 |
| P52479 | Usp10 PE | Ubiquitin carboxyl-terminal hydrolase 10 OS | 0 | 2 |
| Q61335 | Bcap31 PE | B-cell receptor-associated protein 31 OS | 2.02 | 1 |
| Q9CY66 | Gar1 PE | H/ACA ribonucleoprotein complex subunit 1 OS | 2.02 | 0 |
| Q5U458 | Dnajc11 PE | DnaJ homolog subfamily C member 11 OS | 1.01 | 3 |
| P19246 | Nefh PE | Neurofilament heavy polypeptide OS | 2.02 | 0 |
| P35585 | Ap1m1 PE | AP-1 complex subunit mu-1 OS | 2.02 | 1 |
| P28063 | Psmb8 PE | Proteasome subunit beta type-8 OS | 1.01 | 2 |
| Q9CWZ3 | Rbm8a PE | RNA-binding protein 8A OS | 3.02 | 1 |
| Q9JIY5 | Htra2 PE | Serine protease HTRA2, mitochondrial OS | 2.02 | 1 |
| Q9R190 | Mta2 PE | Metastasis-associated protein MTA2 OS | 2.02 | 0 |
| P12815 | Pdcd6 PE | Programmed cell death protein 6 OS | 3.02 | 2 |
| Q9JKV1 | Adrm1 PE | Proteasomal ubiquitin receptor ADRM1 OS | 0 | 1 |
| Q8BRT1 | Clasp2 PE | CLIP-associating protein 2 OS | 0 | 1 |
| P61804 | Dad1 PE | Dolichyl-diphosphooligosaccharide--protein glycosyltransferase subunit DAD1 OS | 0 | 3 |
| P59325 | Eif5 PE | Eukaryotic translation initiation factor 5 OS | 2.02 | 2 |
| Q8R016 | Blmh PE | Bleomycin hydrolase OS | 2.02 | 1 |
| Q9D365 | Spcs3 PE | Signal peptidase complex subunit 3 OS | 1.01 | 0 |
| Q9CQK7 | Rwdd1 PE | RWD domain-containing protein 1 OS | 2.02 | 0 |
| P62305 | Snrpe PE | Small nuclear ribonucleoprotein E OS | 0 | 3 |
| Q6P542 | Abcf1 PE | ATP-binding cassette sub-family F member 1 OS | 1.01 | 0 |
| Q62189 | Snrpa PE | U1 small nuclear ribonucleoprotein A OS | 2.02 | 0 |
| P51863 | Atp6v0d1 PE | V-type proton ATPase subunit d 1 OS | 1.01 | 2 |
| P00375 | Dhfr PE | Dihydrofolate reductase OS | 3.02 | 3 |
| P08556 | Nras PE | GTPase NRas OS | 1.01 | 3 |
| Q9CWL8 | Ctnnbl1 PE | Beta-catenin-like protein 1 OS | 1.01 | 2 |
| Q9D8B3 | Chmp4b PE | Charged multivesicular body protein 4b OS | 1.01 | 1 |
| Q9CWK8 | Snx2 PE | Sorting nexin-2 OS | 0 | 2 |
| Q9D1B9 | Mrpl28 PE | 39S ribosomal protein L28, mitochondrial OS | 2.02 | 0 |
| Q91WD5 | Ndufs2 PE | NADH dehydrogenase [ubiquinone] iron-sulfur protein 2, mitochondrial OS | 2.02 | 0 |
| Q9CQI7 | Snrpb2 PE | U2 small nuclear ribonucleoprotein B'' OS | 1.01 | 2 |
| Q9D0T1 | Nhp2l1 PE | NHP2-like protein 1 OS | 4.03 | 4 |
| P62862 | Fau PE | 40S ribosomal protein S30 OS | 3.02 | 2 |
| Q99KG3 | Rbm10 PE | RNA-binding protein 10 OS | 2.02 | 0 |
| Q9D735 | 2 SV | Uncharacterized protein C19orf43 homolog OS | 2.02 | 1 |
| P18572 | Bsg PE | Basigin OS | 1.01 | 4 |
| Q9D1M4 | Eef1e1 PE | Eukaryotic translation elongation factor 1 epsilon-1 OS | 2.02 | 1 |
| Q6PAR5 | Gapvd1 PE | GTPase-activating protein and VPS9 domain-containing protein 1 OS | 2.02 | 2 |
| Q9D855 | Uqcrb PE | Cytochrome b-c1 complex subunit 7 OS | 1.01 | 0 |
| Q9WV70 | Noc2l PE | Nucleolar complex protein 2 homolog OS | 2.02 | 0 |
| P23492 | Np PE | Purine nucleoside phosphorylase OS | 2.02 | 0 |
| Q8VHE0 | Sec63 PE | Translocation protein SEC63 homolog OS | 2.02 | 0 |
| Q9Z2N8 | Actl6a PE | Actin-like protein 6A OS | 1.01 | 1 |
| Q9Z2W0 | Dnpep PE | Aspartyl aminopeptidase OS | 2.02 | 0 |
| Q80U93 | Nup214 PE | Nuclear pore complex protein Nup214 OS | 0 | 2 |
| Q6NV83 | Sr140 PE | U2-associated protein SR140 OS | 2.02 | 0 |
| P53657 | Pklr PE | Pyruvate kinase isozymes R/L OS | 4.03 | 1 |
| Q8BLN5 | Lss PE | Lanosterol synthase OS | 1.01 | 2 |
| Q9DCC4 | Pycrl PE | Pyrroline-5-carboxylate reductase 3 OS | 1.01 | 2 |
| Q99LX0 | Park7 PE | Protein DJ-1 OS | 2.02 | 1 |
| O54941 | Smarce1 PE | SWI/SNF-related matrix-associated actin-dependent regulator chromatin subfamily E member 1 OS | 2.02 | 0 |
| Q00612 | G6pdx PE | Glucose-6-phosphate 1-dehydrogenase X OS | 2.02 | 0 |
| Q9WVE8 | Pacsin2 PE | Protein kinase C and casein kinase substrate in neurons protein 2 OS | 0 | 2 |
| P68037 | Ube2l3 PE | Ubiquitin-conjugating enzyme E2 L3 OS | 1.01 | 0 |
| Q08093 | Cnn2 PE | Calponin-2 OS | 1.01 | 2 |
| P48774 | Gstm5 PE | Glutathione S-transferase Mu 5 OS | 0 | 1 |
| Q6P5B0 | Rrp12 PE | RRP12-like protein OS | 2.02 | 0 |
| P40630 | Tfam PE | Transcription factor A, mitochondrial OS | 2.02 | 0 |
| P52431 | Pold1 PE | DNA polymerase delta catalytic subunit OS | 0 | 2 |
| Q9QZ88 | Vps29 PE | Vacuolar protein sorting-associated protein 29 OS | 2.02 | 0 |
| Q9D1R9 | Rpl34 PE | 60S ribosomal protein L34 OS | 8.06 | 7 |
| Q9D903 | Ebna1bp2 PE | Probable rRNA-processing protein EBP2 OS | 2.02 | 1 |
| Q99P31 | Hspbp1 PE | Hsp70-binding protein 1 OS | 2.02 | 0 |
| Q8C5L7 | Rbm34 PE | RNA-binding protein 34 OS | 2.02 | 0 |
| P61211 | Arl1 PE | ADP-ribosylation factor-like protein 1 OS | 3.02 | 2 |
| P83882 | Rpl36a PE | 60S ribosomal protein L36a OS | 3.02 | 1 |
| Q8VI84 | Noc3l PE | Nucleolar complex protein 3 homolog OS | 2.02 | 0 |
| Q9CYZ2 | Tpd52l2 PE | Tumor protein D54 OS | 0 | 2 |
| O35130 | Emg1 PE | Probable ribosome biogenesis protein NEP1 OS | 2.02 | 0 |
| Q91V64 | Isoc1 PE | Isochorismatase domain-containing protein 1 OS | 0 | 2 |
| O55142 | Rpl35a PE | 60S ribosomal protein L35a OS | 3.02 | 1 |
| P36916 | Gnl1 PE | Guanine nucleotide-binding protein-like 1 OS | 2.02 | 0 |
| Q8BLN6 | Unc80 PE | Protein unc-80 homolog OS | 2.02 | 0 |
